# African swine fever virus pEP364R acts as an important inflammatory-inducing factor to activate NLRP3 inflammasome-mediated pyroptosis by regulating DDX3X

**DOI:** 10.1371/journal.ppat.1013874

**Published:** 2026-02-25

**Authors:** Qian Li, XuSheng Ma, XiaoFen Shang, XiaoFeng Nian, Ruoqing Mao, ZhiKuan Luo, ZiXiang Zhu, Yi Ru, Fan Yang, WeiJun Cao, YanYan Chang, YiGang Xu, Asad Jahangir, Syed Mubash-Sher Tajmir, XiaoHong Ma, YanYan Zhang, YuGuang Fu, JingJing Pei, HaiXue Zheng

**Affiliations:** 1 State Key Laboratory of Veterinary Etiological Biology, College of Veterinary Medicine, Lanzhou University Lanzhou Veterinary Research Institute, Chinese Academy of Agricultural Sciences, Lanzhou, China; 2 Gansu Province Research Center for Basic Disciplines of Pathogen Biology, Lanzhou, China; 3 Changchun Veterinary Research Institute, Chinese Academy of Agricultural Sciences, Changchun, Jilin, China; 4 China-Malaysia National Joint Laboratory, Biomedical Research Center, Life Science and Engineering College, Northwest Minzu University, Lanzhou, China; 5 College of Veterinary Medicine, Northeast Agricultural University, Harbin, China; 6 Key Laboratory of Applied Technology on Green-Eco-Healthy Animal Husbandry of Zhejiang Province, College of Animal Science & Technology, Zhejiang A&F University, Hangzhou, P.R. China; 7 Key Laboratory of Applied Technology on Green-Eco-Healthy Animal Husbandry of Zhejiang Province, College of Veterinary Medicine, Zhejiang A&F University, Hangzhou, P.R. China; University of California at Los Angeles, UNITED STATES OF AMERICA

## Abstract

African swine fever virus (ASFV) infection causes a severe hemorrhagic disease in pigs, characterized by excessive inflammatory responses and tissue damage, posing substantial threats to the pig industry worldwide. Given the lack of vaccines and effective antiviral treatments, as well as the largely unknown functions of most ASFV-encoded proteins, it’s urgent to study the proteins that are crucial in triggering inflammatory responses and how they do so. This study demonstrated that ASFV exploited the NOD-, LRR- and pyrin domain-containing protein 3 (NLRP3) inflammasome to induce pyroptosis and inflammatory responses, effectively replacing the non-functional porcine AIM2 pseudogene. Screening over 150 proteins encoded by the ASFV genome, EP364R was identified as the viral factor responsible for driving NLRP3-mediated pyroptosis and high-level cytokine production. Ectopic expression of EP364R in mice elicited significant upregulation of serum pro-inflammatory cytokines and splenomegaly, while its expression in bone marrow-derived macrophages (BMDMs) from NLRP3-knockout mice abrogated pyroptosis and related effects. Mechanistic investigation revealed that the helicase DDX3X acted as a molecular bridge, enabling EP364R to interact with NLRP3 to promote the aggregation and activation of inflammasomes. Depletion of DDX3X abolished EP364R’s ability to induce NLRP3-dependent pyroptosis and pro-inflammatory cytokine production. We found that the NACHT domain of porcine NLRP3 interacted with DDX3X, and EP364R established a connection with the NACHT and LRR domains of NLRP3 through DDX3X. However, EP364R bound to all the domains of DDX3X. Molecular docking analysis revealed that DDX3X interacted with EP364R through a spatially defined interface, thereby exerting its function. Furthermore, a natural compound library was employed to screen functional compounds targeting EP364R, and HAMNO was identified as an inhibitor that bound to E256, K259, and D260 of EP364R, consequently suppressing ASFV replication. Our findings explain how ASFV triggers pyroptosis and excessive cytokine release, and identify a potent small-molecule inhibitor of ASFV, aiding the development of vaccines and therapies to prevent and control African swine fever (ASF) caused by ASFV infection.

## Author summary

ASF has caused huge economic losses to the global pig industry. Currently, there is no commercially available drug or vaccine. Excessive inflammatory responses triggered by ASFV infection in domestic pig correlate with the severe clinical manifestations of ASF. Virus-induced inflammatory cell death, including pyroptosis, is a major contributor to systemic hyperinflammation and tissue damage. This study revealed that ASFV infection induced high-level cytokine production in infected host cells, particularly in mixed immune cells. Given that the porcine AIM2 gene is a pseudogene, we demonstrated that ASFV exploited the NLRP3 inflammasome to induce pyroptosis and the production of pro-inflammatory cytokines. Genome-wide screening identified the ASFV-encoded EP364R protein as a candidate factor promoting NLRP3 inflammasome-mediated pyroptosis and inflammatory responses. Expression of EP364R in mice induced upregulation of pro-inflammatory cytokines and splenomegaly. Mechanistically, the EP364R protein binds to NLRP3 through DDX3X, aiding in the assembly of the NLRP3 inflammasome. This effect was eliminated after DDX3X depletion. Critically, EP364R interacts with DDX3X in a spatially organized manner that promotes pyroptotic cell death. Notably, a natural compound, HAMNO, was identified as an inhibitor targeting the EP364R protein to suppress ASFV replication. Therefore, this work elucidated that ASFV utilized the EP364R protein to promote NLRP3 inflammasome activation and assembly via DDX3X, driving pyroptosis and excessive inflammatory responses, which may be one of the reasons why ASFV infection causes tissue damage.

## Introduction

ASFV, the only member of the *Asfarviridae* family, causes destructive and highly contagious diseases in domestic swine. To date, there is still no effective vaccine and therapeutics for ASF, which allows it to spread globally. ASFV is a large, enveloped, double-stranded DNA virus with a genome size ranging from 170–193 kb and encoding more than 150 proteins [1–4]. The clinical signs in domestic pigs after ASFV infection include high fever, skin cyanosis, hemorrhage, diarrhea, and even sudden death. Additionally, ASFV infection triggers a strong inflammatory response in infected pigs, such as TNF-α and ILs of pro-inflammatory cytokines are significantly increased in the serum after infection. Some studies have reported that low-virulence ASFV strains induced higher levels of inflammatory factors than high-virulence strains [5,6]. However, sharply increased cytokines expression in the late stages of virulent ASFV strains infection was observed [7].

Pyroptosis is an inflammatory form of programmed cell death carried out by the gasdermin family (In humans, six paralogous genes have been identified: GSDMA-E, and DFNB59), characterized by rupture of the cytoplasmic membrane, leading to the production of pro-inflammatory cytokines and the release of cellular contents. This process activates a strong inflammatory response and is involved in many pathophysiological processes [8,9]. Both the canonical caspase‑1 (Casp-1)–dependent pathway and the noncanonical Casp‑4/5/11–dependent pathways are essential components of the pyroptosis signaling transduction. In the non‑canonical pathway, signals such as lipopolysaccharide (LPS) have been shown to activate Casp‑4 and Casp‑5 in humans and Casp‑11 in mice. Subsequently, these caspases cleave GSDMD, leading to pyroptosis [10–12]. Additionally, granzyme B and Casp-1/3/7 can also induce pyroptosis by cleaving GSDME [13]. Among the GSDMs, GSDMD has been extensively studied and is initially recognized as the key effector in inflammasome-triggered pyroptosis. In the canonical Casp‑1-dependent;pyroptosis pathway, cells’ pattern recognition receptors (PRRs) detect foreign pathogens and activate NF-κB, which then translocates into the nucleus and triggers the transcription of pro-IL-1β, NLRP3, and pro-Casp-1 [14]. Then, damage-associated molecular patterns (DAMPs) initiate the classical pyroptosis cascade, which involves inflammasomes (such as AIM2, NLRP3), interferon‑γ‑inducible protein‑16 (IFI16), and various NOD‑like receptors. For instance, activated NLRP3 oligomers recruit ASC to form ASC specks, on which pro-Casp-1 clusters to induce self-cleavage and maturation of pro-Casp-1 into Casp-1. In addition, Casp-1 cleaves inactive pro-IL-1β and -GSDMD, converting them into bioactive forms (IL-1β/N-GSDMD). N-GSDMD then translocates to the cell membrane and forms pores to trigger inflammatory responses and pyroptosis [12,15–17].

Previous studies reported that pathogen infection could induce pyroptosis and an inflammatory response. For instance, SARS-CoV-2 and influenza virus H5N1 are involved in pyroptosis and inflammation [18–24]; Parasitic Infection with Nippostrongylus triggers pyroptosis in mouse intestinal epithelial cells through GSDMC-mediated pathways [25]. The N protein and ORF3a of SARS-CoV-2 promote NLRP3 inflammasome activation and induce hyperinflammation [26,27], and the non-structural protein 6 targets ATP6AP1 to trigger NLRP3-dependent pyroptosis [28]. Recent studies indicated that a viroporin of ASFV pB169L altered Ca^2+^ homeostasis and induced proinflammatory responses [29]; Yang Chen et al., proposed that ASFV-induced pyroptosis via the TLR4/MyD88/MAPK/NF-κB signaling pathway and the ROS-NLRP3 inflammasome signaling cascade [30]. And Wu et al., also found that ASFV pI177L induced NF-κB activation through TRAF6-TAK1 axis and promoted NLRP3 inflammasome assembly [31]. However, most research focused on the inhibitory effect of ASFV protein on inflammation, such as ASFV pMGF505-7R interacted with IKKα and NLRP3 to suppress cGAS-STING signaling and NLRP3 inflammasome-mediated IL-1β production [32], and the ASFV pS273R protease specifically cleaved GSDMD to antagonize pyroptosis [33]. Infection with ASFV ultimately manifests as severe inflammatory pathology in pigs. Critically, the direct activation of pyroptosis and inflammatory responses by ASFV proteins are still limited. Therefore, understanding how ASFV causes pyroptosis and inflammation is essential for managing the acute mortality caused by ASFV infection.

In this study, we observed that ASFV infection of BMDMs, especially peripheral blood lymphocytes (PBMCs), triggered a significant increase in pro-inflammatory cytokines and cell death. Further, we demonstrated that ASFV induced NLRP3-mediated pyroptosis and the production of inflammatory cytokines. By screening all ASFV-encoded proteins, EP364R was identified as a candidate protein to induce the production of inflammatory cytokines and the occurrence of pyroptosis. Compared with the parental wild-type ASFV strain, ASFV with EP364R knockdown induced lower levels of cytokines and pyroptosis. Using NLRP3 knockdown cells or BMDMs from NLRP3 knockout mice, we found that EP364R-induced pyroptosis was dependent on the NLRP3 signaling pathway. Mechanistically, EP364R interacts with DDX3X to activate NLRP3-Casp-1-mediated pyroptosis. Knockdown or knockout of DDX3X reduces EP364R-induced cell pyroptosis. After DDX3X recovery, the functional effect of EP364R was restored. In addition, through co-immunoprecipitation (Co-IP) and AI model analysis, we found that the sites of EP364R interacted with all the domains of DDX3X, promoting NLRP3-ASC complex formation. In addition, EP364R established a connection with the NACHT and LRR domains of NLRP3 through DDX3X. Furthermore, through screening of the compound library, we found that the small molecule HAMNO, targeting EP364R, could inhibit ASFV replication. In conclusion, this study revealed the activation effects of ASFV EP364R on pyroptosis and inflammatory responses, which deepens our understanding of ASFV-induced inflammation and facilitates the design of attenuated vaccines or antiviral drugs to control ASF.

## Results

### ASFV infection induced inflammatory cytokines production

**Fig 1:**
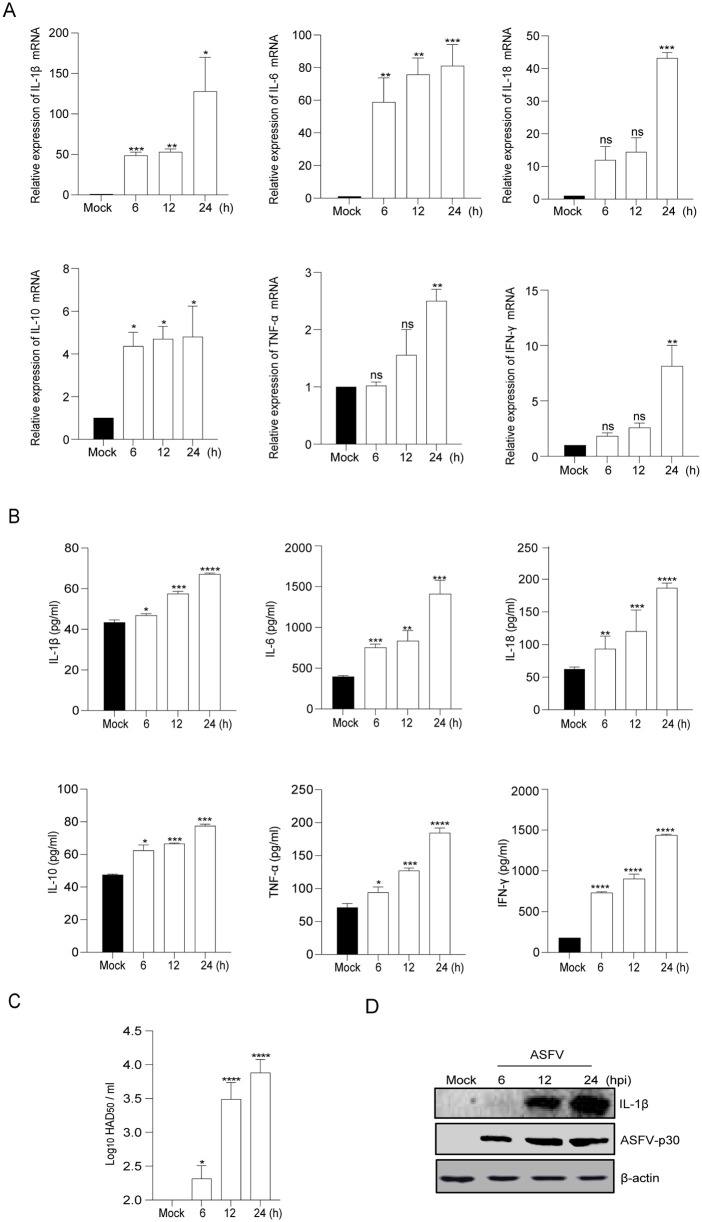
ASFV induced the production of proinflammatory cytokines. **(A and B)** Bone-marrow-derived macrophages (BMDMs) were infected with ASFV at an MOI of 1 and harvested at 6, 12, and 24 h post-infection (hpi). The transcriptional levels (**A**) and secretion levels (**B**) of IL-1β, IL-6, IL-18, IL-10, TNF-α, and IFN-γ in the cells and cell culture supernatant were measured by qPCR and ELISA, respectively. **(C)** ASFV infected with BMDMs for indicated time point. The ASFV HAD_50_ in BMDMs was determined. **(D)** ASFV infected with BMDMs for indicated time point. ASFV p30 protein expression in BMDMs was detected by Western blot (WB). Data are presented as mean ± SEM. *p < 0.05, **p < 0.01, ***p < 0.001; statistical significance was determined by one-way ANOVA.

Previous studies have demonstrated that pro-inflammatory cytokines were increased in the serum after ASFV infection [34]. To assess whether ASFV infection induced an immune response in infected macrophages, porcine BMDMs were infected with ASFV (strain GS/CN/2018) at the indicated times. The results showed that IL-1β, IL-6, IL-18, IL-10, TNF-α, and IFN-γ were upregulated at both the mRNA and protein levels post-ASFV infection (Fig 1A and 1B). These pro-inflammatory cytokines increase over time during ASFV infection. Absolute quantitative PCR was employed to assess ASFV loads in BMDMs post ASFV infection, revealing an increase in ASFV genome copies over the infection course (Fig 1C). Consistently, as shown in Fig 1D, the secretion of matured IL-1β (17KDa) in supernatant was increased during ASFV infection.

These results indicated that ASFV infection induced the production of pro-inflammatory cytokines.

### ASFV infection induced cell pyroptosis

**Fig 2:**
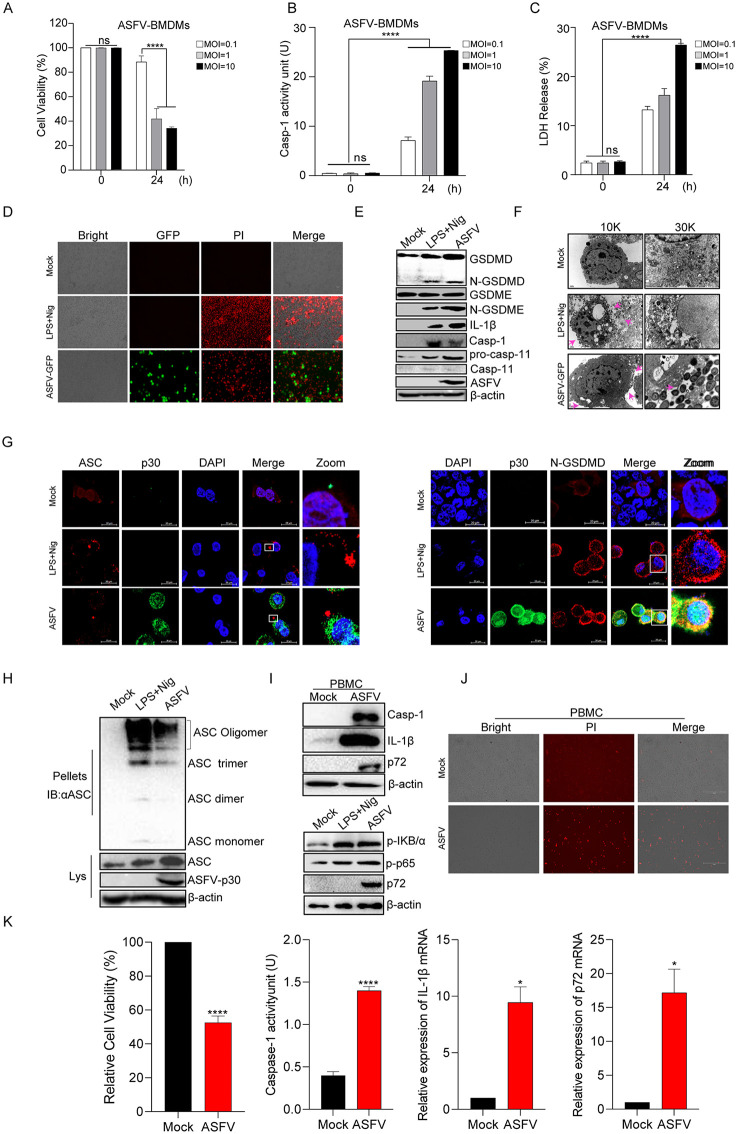
ASFV infection induced pyroptosis. **(A–C)** BMDMs were infected with ASFV (MOI = 0.1/1/10) for 24 **h.** Cell viability **(A)**, Casp-1 activity **(B)**, and LDH release (**C**) were measured using CCK-8 assay kit, Casp-1 activity assay kit, and LDH assay kit, respectively. **(D)** PAMs were infected with ASFV-GFP (MOI = 1) for 24 h, followed by propidium iodide (PI) staining to observe cell death. A positive control (LPS [60 ng/mL for 8 **h]** + nigericin [Nig, 2 μM for 2 **h]**) was included. **(E)** BMDMs were infected with ASFV (MOI = 1) for 24 **h.** Expression of pyroptosis marker proteins (Casp-1, IL-1β, and GSDMD-N) was analyzed by WB. **(F)** PAMs were infected with ASFV at an MOI of 1 for 24 h, fixed with 2% glutaraldehyde, and processed for transmission electron microscopy (TEM). Arrows indicate plasma membrane rupture. (**G**) iPAM cells were infected with ASFV at an MOI of 1. Formation of ASC specks and membrane localization of N-GSDMD were observed by immunofluorescence assay (IFA). (**H**) iPAM cells were infected with ASFV (MOI = 1) or positive control (LPS + Nig). ASC oligomerization was detected by WB using disuccinimidyl suberate (DSS) crosslinking. **(I)** PBMCs were infected with ASFV (MOI = 1) for 24 **h.** Expression of pyroptosis markers (Casp-1, IL-1β, N-GSDMD) and phosphorylation of IκBα and p65 in the NF-κB pathway were analyzed by WB. **(J)** PBMCs were infected with ASFV (MOI = 1) for 24 h, followed by PI staining to observe cell death. **(K)** PBMCs were infected with ASFV (MOI = 1) for 24 hours. Cell viability, Casp-1 activity, IL-1β transcription, and ASFV p72 mRNA level were assessed by CCK-8 assay, Casp-1 activity kit, qPCR, and qPCR, respectively. Data are presented as mean ± SEM. *p < 0.05, **p < 0.01, ***p < 0.001; statistical significance was determined by one-way ANOVA.

IL-1β secretion was increased after ASFV infection, and its maturation correlated with the occurrence of cell pyroptosis. We further evaluated whether ASFV infection led to cellular pyroptosis. Cell viability results indicated that cell death was increased following ASFV (MOI = 1) infection in BMDMs (Fig 2A). Consistently, damage-associated molecular patterns (DAMPs) markers and lactate dehydrogenase (LDH) release, and Casp-1 activity, were also elevated in cell culture medium after ASFV infection (Fig 2B and 2C). Further, as shown in Fig 2D, we observed that compared to the control, positive control (LPS+Nigercin) and ASFV-GFP (ASFV genome containing GFP tag) treatment obviously triggered cell death (dead cells could be stained with propidium Iodide (PI)). Moreover, ASFV infection induced N-GSDMD, IL-1β, and Casp-1 maturation (Fig 2E). Electron microscopy results showed that ASFV infection induced cell swelling and cell membrane damage (Fig 2F). The fluorescence results also showed that ASFV infection induced the formation of ASC specks and the enrichment of mature N-GSDMD on the cell membrane (Fig 2G). ASC oligomerization was increased after ASFV infection (Fig 2H).

To verify the ability of ASFV to induce pyroptosis in other cell types., PBMCs were isolated and infected with ASFV. We found that ASFV infection induced the maturation of IL-1β and Casp-1 in PBMCs (Fig 2I). The production of pro-IL-1β mRNA is associated with NF-κB activation; thus, we further examined the phosphorylation of p65. The results revealed that phosphorylated IκBα and p65 were enhanced by ASFV infection (Fig 2I). PI staining results indicated the induction of cell death following ASFV infection (Fig 2J). Similarly, ASFV infection in PBMCs caused reduced cell viability and elevated Casp-1 activity (Fig 2K). Additionally, we observed a significant increase in pro-IL-1β mRNA in PBMCs following ASFV infection (Fig 2K). In addition, previous study showed that ASFV active Casp-3/Casp-4 to cleave GSDMA to regulate pyroptosis [35], therefore, we used Casp-3 and Casp-4 inhibitors to assess the function of ASFV-induced Casp-1-mediated pyroptosis. The results demonstrated that without Casp-3 and Casp-4, ASFV also induced pyroptosis (S1A-S1C Fig).

Taken together, these results suggested that ASFV infection activated Casp-1-mediated pyroptosis and IL-1β production in BMDMs.

### ASFV infection induced cell pyroptosis via the NLRP3 inflammasome

**Fig 3:**
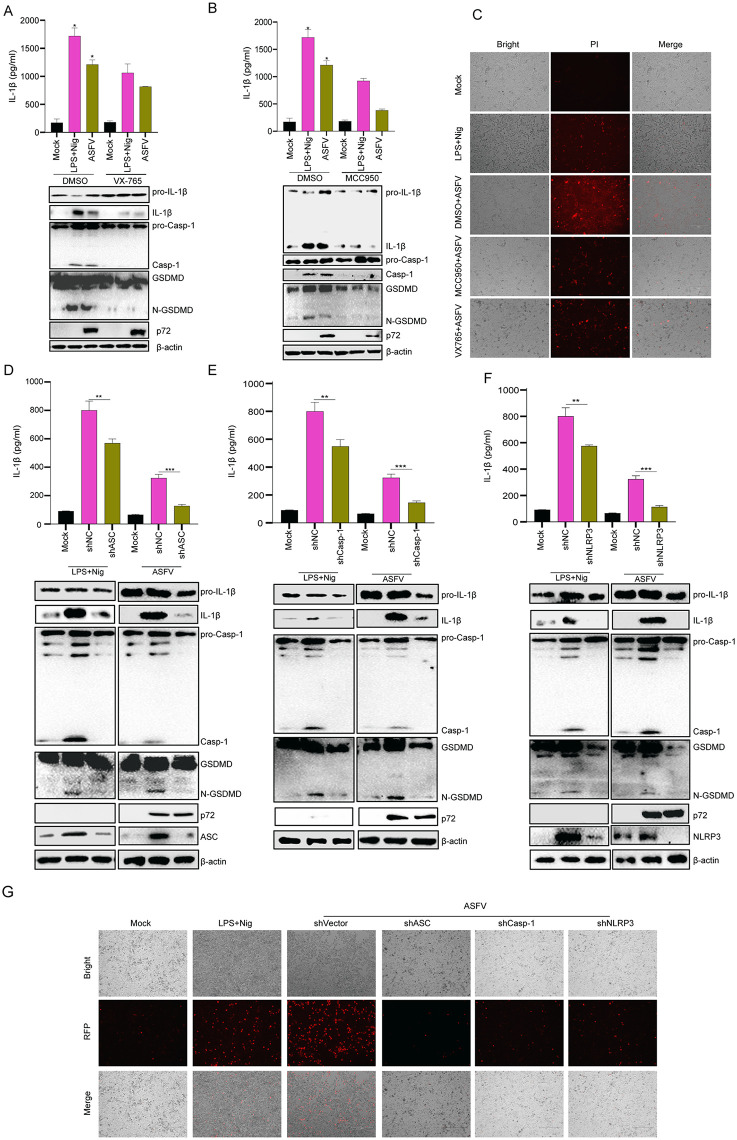
ASFV induced pyroptosis via the NLRP3 inflammasome. **(A and B)** PAMs were pretreated with the Casp-1 inhibitor VX765 (10 μM, 2 **h)** (**A**) or the NLRP3 inhibitor MCC950 (10 μM, 1 **h) (B)**, followed by ASFV infection (MOI = 1) for 24 **h.** DMSO was used as a control. IL-1β secretion was measured by ELISA, and pyroptosis biomarkers (Casp-1, IL-1β, N-GSDMD) expression were determined by WB. **(C)** PAMs were pretreated with VX765 or MCC950 as in **(A and B)**, infected with ASFV (MOI = 1) for 24 h, and subjected to PI staining. ASFV-induced cell death was observed. **(D–F)** PAMs were transfected with shASC **(D)**, shCasp-1 **(E)**, or shNLRP3 (**F**) for 72 h, followed by ASFV infection (MOI = 1) for 24 h. shNC was used as a negative control. ELISA used for IL-1β secretion detection, and pyroptosis biomarkers expression were determined by WB. **(G)** PAMs were transfected with shASC, shCasp-1, or shNLRP3 for 72 h, infected with ASFV (MOI = 1) for 24 h, and subjected to PI staining. LPS + Nig served as a positive control. Data are presented as mean ± SEM. *p < 0.05, **p < 0.01, ***p < 0.001; statistical significance was determined by one-way ANOVA.

AIM2 senses foreign pathogen DNA to activate inflammasome-mediated Casp-1 maturation, IL-1β production, and cell pyroptosis. Although ASFV is a DNA virus, it has been demonstrated that AIM2 is a pseudogene in pigs [36]. Therefore, we investigated whether ASFV-induced pyroptosis is associated with the NLRP3 inflammasome. ASFV-infected BMDMs were treated with VX765 (Casp-1 inhibitor) or MCC950 (NLRP3 inhibitor). As shown in Fig 3A and 3B, similar to LPS and Nigericin stimulation (LPS + Nig), ASFV-induced maturation and secretion of Casp-1, IL-1β, and GSDMD-N were significantly inhibited after treatment with VX765 or MCC950. Meanwhile, we observed that PI-stained dead cells were reduced after treatment with MCC950 or VX765 during ASFV infection (Fig 3C).

Furthermore, the short hairpin RNA (shRNA) of NLRP3 inflammasome components was used to assess cell pyroptosis and IL-1β production following ASFV infection. As shown in S2A Fig, the expression of ASC, NLRP3, and Casp-1 proteins was interfered by their specific shRNA. After specific shRNA transfection, we observed a significant decrease in IL-1β secretion during ASFV infection (Fig 3D–3F). Moreover, the levels of mature Casp-1, IL-1β, and N-GSDMD were decreased following ASFV infection after specific shRNA transfection. (Fig 3D–3F). PI staining results also showed that cell death was reduced after ASC, NLRP3, or Casp-1 shRNA transfection during ASFV infection (Fig 3G).

### ASFV EP364R induced IL-1β production and cell pyroptosis

**Fig 4:**
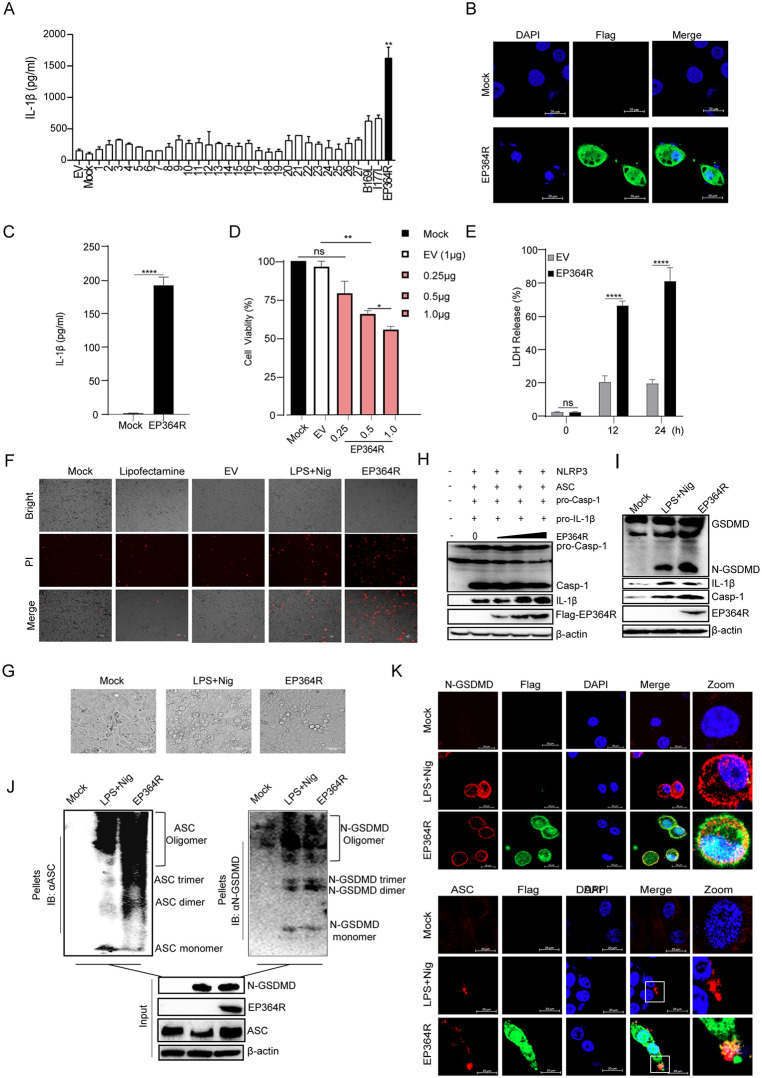
ASFV pEP364R induced IL-1β production and pyroptosis. **(A)** PK-15 cells, with activated NLRP3 inflammasome components, were transfected with 2 μg of each of the 185 plasmids encoding ASFV proteins. IL-1β secretion was screened by ELISA to identify proteins inducing high IL-1β levels. (**B**) iPAMs cells were transfected with 2 μg of EP364R plasmid. At 24 h post-transfection (h.p.t), cells were fixed and the subcellular localization of EP364R was observed by confocal microscopy. Scale bars, 20 μm. **(C)** BMDMs cells were transfected with 2 μg of EP364R plasmid. IL-1β secretion in the supernatant was measured by ELISA at 24 **h.** (**D**) iPAMs were transfected with increasing concentrations (0.25, 0.5, 1.0 μg) of EP364R plasmid, with 1 μg of empty vector (EV) plasmid as a control. At 24 h, 10 μl of CCK-8 reagent was added, followed by incubation at 37°C for 1 hour. Absorbance was measured at 450 nm. (**E**) iPAMs were transfected with 2 μg of EP364R plasmid. Lactate dehydrogenase (LDH) release was detected using an LDH assay kit at 0, 12, and 24 **h.** (**F**) iPAMs were transfected with 2 μg of EP364R plasmid, alongside blank control, positive control [LPS (60 ng/mL for 8 **h)** + Nigericin (Nig, 2 μM for 2 **h)**], liposome (4 μl) control, and negative control (EV, 2 μg). At 24 h, cells were stained with propidium iodide (PI) and cell death was observed by fluorescence microscopy. **(G)** Cell morphology was observed by microscopy 24 h after transfection of iPAMs with 2 μg of EP364R plasmid. (**H**) iPAMs were co-transfected with plasmids for NLRP3 (3 μg), pro-IL-1β (3 μg), ASC (1 μg), pro-Casp-1 (1 μg), and increasing amounts of EP364R (0, 2, 4, 6 μg). Cells were harvested and lysed 24 h p.t., and IL-1β secretion was analyzed by WB. (**I**) iPAMs were transfected with 2 μg of EP364R plasmid, with LPS + Nig treatment as a positive control. Cells were lysed 24 h p.t., and the expression of Casp-1, IL-1β, and GSDMD-N was analyzed by WB. (**J**) iPAMs were transfected with 2 μg of EP364R plasmid, with LPS + Nig as a positive control. At 24 h p.t., cell lysates were centrifuged. The supernatant was prepared as the ‘input’ sample. The cell pellet was resuspended in 100 μl PBS, cross-linked with 2 mM DSS at 37°C for 30 min, and then directly mixed with protein loading buffer to prepare the ‘pellet’ sample. ASC and GSDMD-N oligomerization were detected by WB. (**K**) iPAMs were transfected with 2 μg of EP364R plasmid. At 24 h p.t., cells were fixed, and ASC speck formation and GSDMD-N localization were observed by confocal microscopy. Scale bars, 20 μm. A P value less than 0.05 was considered statistically significant. *p < 0.05, **p < 0.01, ***p < 0.001.

To elucidate the underlying mechanism of NLRP3 inflammasome-mediated cell pyroptosis during ASFV infection, we constructed a database of 185 ASFV-encoded proteins. We assayed their capacity to enhance IL-1β secretion in PK15 cells activated by NLRP3-inflammasome components. Through this screening process, ASFV EP364R was identified as a candidate to increase IL-1β secretion during the expression of NLRP3-inflammasome components ASC, NLRP3, pro-Casp-1, and pro-IL-1β (Fig 4A). Additionally, we also found that EP364R was a late-stage expression protein during ASFV infection and is localized in both the cytoplasm and nucleus of BMDMs (S3A Figs and 4B).

Furthermore, to evaluate the capacity of EP364R alone to induce IL-1β production, EP364R was transfected into BMDMs, and the induction of IL-1β secretion was significantly increased after EP364R expression (Fig 4C). The expression of EP364R reduced cell viability in a dose-dependent manner (Fig 4D). Consistently, LDH release and Casp-1 activity also were increased in a dose-dependent manner after EP364R transfection (Figs 4E and S3B).

Moreover, PI staining results indicated that EP364R-expressed cells undergo significant cell death (Fig 4F). Further, some cells exhibited pyroptotic features, such as rounded swelling, following LPS + Nig or EP364R stimulation (Fig 4G). Electron microscope results also showed that EP364R induced typical morphological features of pyropotosis, such as cell swelling and vesicle formation at the membrane edge (S3C Fig). To further confirm the capacityof EP364R to induce pyroptosis, cells were co-transfected with different doses of EP364R and NLRP3 inflammasome components. We found that EP364R enhanced the maturation of Casp-1, and IL-1β protein expression (Fig 4H). EP364R alone also induced GSDMD-N, Casp-1, and IL-1β expression (Fig 4I). Additionally, we observed that N-GSDMD and ASC oligomerization occurred when EP364R was present (Fig 4J). Consistently, GSDMD-N and ASC specks formed during EP364R expression (Fig 4K).

The first signal of NLRP3 inflammasome activation requires NF-κB activation; therefore, we further evaluated the effect of EP364R on p65 phosphorylation. The results showed that EP364R induced the phosphorylation of IκBα and p65 (S3D Fig). Furthermore, the mRNA levels of pro-inflammatory cytokines, including IL-1β, IL-6, and TNF-α, as well as IFN-β, were increased after EP364R expression (S3E Fig). In addition, EP364R also induced DAMPs release, such as Casp-1, CRT, and ATP (S3F Fig)

Taken together, these results indicated that EP364R induced pyroptosis and IL-1β production.

### The EP364R protein induced inflammation in mice

**Fig 5:**
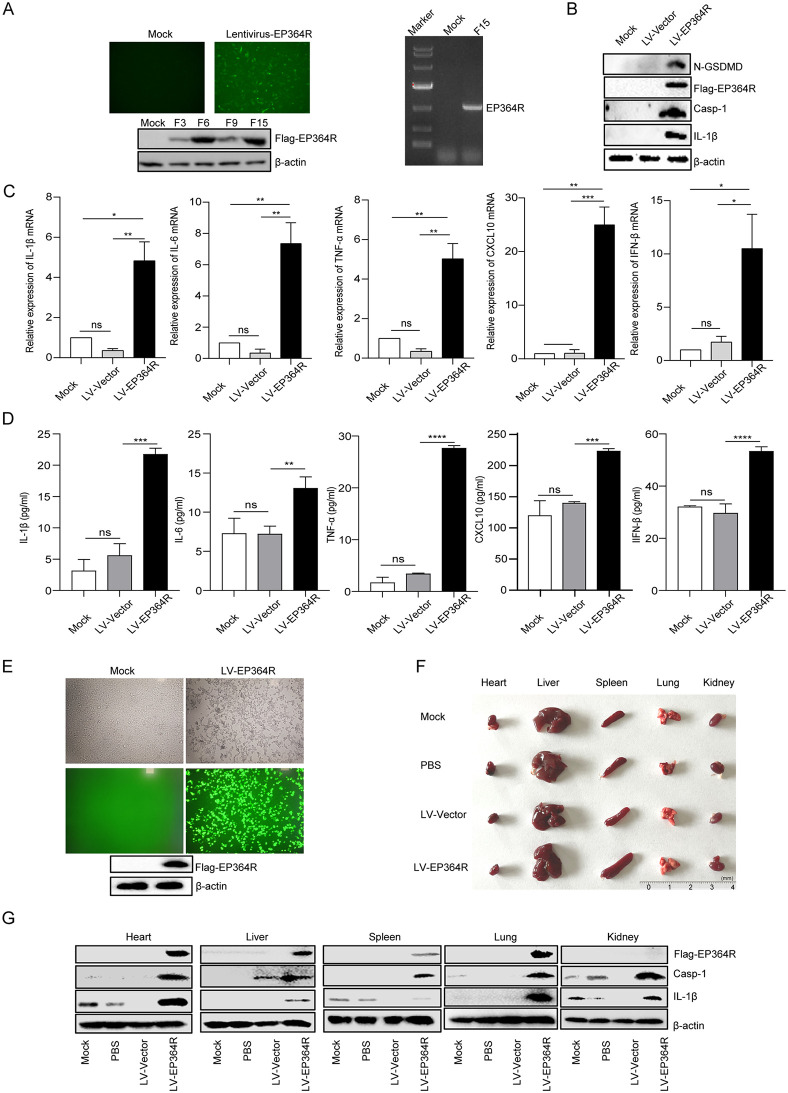
pEP364R triggered inflammation in mice. (**A**) The pHBLV-EP364R lentiviral overexpression plasmid (10 μg) was co-transfected with lentiviral packaging helper plasmids psPAX2 (10 μg) and pMD2.G (10 μg) into HEK293T cells. Supernatants containing lentivirus were collected at 48 and 72 h p.t., and purified, and used to infect iPAMs. Stable puromycin-resistant cells expressing green fluorescent protein (GFP) were selected. Stable EP364R expression was confirmed by WB and PCR. (**B**) Casp-1, IL-1β, and GSDMD-N in EP364R-overexpressed iPAMs (LV-EP364R) were detected by WB, iPAMs expressing only GFP and resistance genes served as negative controls (LV-Vector), and Mock cells were normal iPAMs. (**C-D**) Transcriptional and secretion levels of cytokines IL-1β, IL-6, TNF-α, CXCL10, and IFN-β were measured by qPCR and ELISA in EP364R-overexpressed iPAMs. (**E**) Successful EP364R overexpressed lentivirus was purified and determined by WB at 72 h p.t. (**F**) Mice were intravenously injected twice with 5 × 10^7^ TU of purified EP364R overexpression lentivirus, with a 48-h interval. Mice were randomly selected from each group and euthanized, and pathological changes in the heart, liver, spleen, lung, and kidney were examined. (**G**) Mice from the LV-EP364R and LV-Vector groups were randomly selected and euthanized. Tissues (heart, liver, spleen, lung, kidney; 1 g each) were harvested, homogenized, and proteins were extracted using tissue lysis buffer. Expression of IL-1β was detected by WB. And *p* value less than 0.05 was considered statistically significant. *p < 0.05, **p < 0.01, ***p < 0.001.

To better determine the effect of the EP364R protein on inflammation, a Flag-EP364R and GFP stable overexpression porcine iPAM cells were constructed. Puromycin was employed to select the transfected cells until the 15th generation. The fluorescence, PCR, and WB were used to determine the constructed cells, and the results showed that the EP364R gene was successfully integrated into the cell genome, and the EP364R was stably expressed (Fig 5A and 5B).

Additionally, we observed that GSDMD-N, Casp-1, and IL-1β maturation occurred in EP364R-expressing cells (Fig 5B). Furthermore, the mRNA transcription and secretion of pro-inflammatory cytokines of IL-1β, IL-6, TNF-α, CXCL10, and IFN-β were increased in EP364R-expressed cells (Fig 5C and 5D).

Moreover, mice were infected with the purified EP364R lentivirus (Fig 5E), and we found that EP364R induced spleen enlargement (Fig 5F). Additionally, maturation of Casp-1 and IL-1β was observed in heart, liver, spleen, lung, and kidney (Fig 5G). IL-1β, TNF-α, IL-6, and IFN-β secretion were increased in the heart, liver, spleen, lung, kidney, and serum during EP364R expression (S4A and S4B Fig). Taken together, the results indicated that EP364R expression induced inflammation in mice.

### ASFV EP364R deficiency impaired pyroptosis and IL-1β production

**Fig 6:**
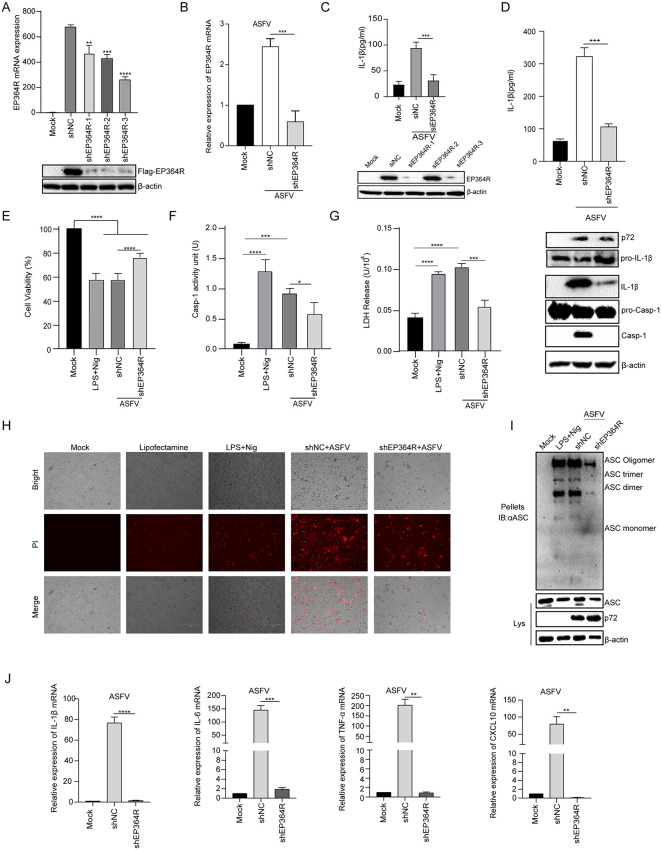
ASFV pEP364R deficiency impaired pyroptosis and IL-1β production. (**A**) iPAMs were transfected with 2 μg of small hairpin RNA (shEP364R-1, -2, or -3) plasmids. At 72 h p.t., cells were transfected with 2 μg of EP364R plasmid for 24 h. small hairpin RNA Nonspecific shRNA (shNC) as a negative control. RNA was extracted for qPCR, and cells were lysed for WB analysis to screen for the most effective shEP364R. (**B**) iPAMs were transfected with 2 μg of shEP364R-3 plasmid. At 72 h p.t., cells were infected with ASFV (MOI = 1). EP364R transcription levels were validated by qPCR 24 h p.i (hours post infection). (**C**) iPAMs were transfected with 50 nM of the selected siEP364R. At 72 hours post-transfection, cells were infected with ASFV at an MOI of 1. The culture supernatants were collected 24 hours post-infection, processed through repeated freeze-thaw cycles and lyophilization, and then analyzed by ELISA to quantify secreted IL-1β. (**D**) iPAMs were transfected with 2 μg of shEP364R-3 plasmid. At 72 h p.t., cells were infected with ASFV (MOI = 1) for 24 **h.** IL-1β secretion was measured by ELISA, and expression of Casp-1, IL-1β, and GSDMD-N was analyzed by WB. (**E**) iPAMs were transfected with 2 μg of shEP364R-3 plasmid. At 72 h p.t., cells were infected with ASFV at MOI = 1 for 24 **h.** CCK-8 reagent (10 μl) was added, followed by incubation at 37°C for 1 h, and absorbance was measured at 450 nm. LPS and Nigericin stimulation group as a positive control. (**F**) iPAMs were transfected with 2 μg of shEP364R-3 or shNC plasmid. At 72 h p.t., cells were infected with ASFV at MOI = 1 for 24 **h.** Intracellular Casp-1 activity was measured using a Caspase-1 activity assay kit. (**G**) iPAMs were transfected with 2 μg of shEP364R-3 plasmid. At 72 h p.t., cells were infected with ASFV at MOI = 1 for 24 **h.** LDH release was detected using an LDH assay kit. (**H**) iPAMs were transfected with 2 μg of shEP364R-3 plasmid. At 72 h p.t., cells were infected with ASFV at MOI = 1 for 24 **h.** Cells were stained with PI (10 μl) and cell death was observed by fluorescence microscopy. (**I**) iPAMs were transfected with 2 μg of shEP364R-3 plasmid. At 72 h p.t., cells were infected with ASFV at MOI = 1 for 24 **h.** Cell lysates were centrifuged; the supernatant was prepared as the ‘input’ sample. The pellet was resuspended in PBS, cross-linked with DSS, incubated at 37°C for 30 min, and mixed with loading buffer to prepare the ‘pellet’ sample. ASC oligomerization was detected by WB. (**J**) iPAMs were transfected with 2 μg of shEP364R-3 plasmid. At 72 h p.t., cells were infected with ASFV at MOI = 1 for 24 **h.** RNA was extracted, and transcriptional levels of IL-1β, IL-6, TNF-α, CXCL10, and EP364R were measured by qPCR. A P value less than 0.05 was considered statistically significant. *p < 0.05, **p < 0.01, ***p < 0.001.

We next investigated the effects of EP364R on ASFV-induced pyroptosis and inflammation. EP364R shRNAs were determined to interfere with EP364R expression, as illustrated in Fig 6A and 6B. shEP364R-3 exhibited the strongest capacity to interfere with EP364R expression. EP364R and shEP364R-3 were co-transfected into iPAMs, and IL-1β secretion was measured. As shown in Fig 6C, siEP364R impaired ASFV-induced IL-1β secretion compared to siRNA control (siNC). Then, ASFV-infected BMDMs were subjected to EP364R shRNA treatment. The inhibition of EP364R expression significantly reduced ASFV-induced IL-1β secretion and Casp-1 expression (Fig 6D). This also motivates us to further investigate the effect of EP364R inhibition on ASFV-induced NF-κB activation; the results showed that the phosphorylation of p65 and IκBα induced by ASFV was inhibited when EP364R expression was disrupted (S5A Fig).

Furthermore, cell viability results showed that inhibition of EP364R expression reduced ASFV-induced cell viability loss (Fig 6E). Consistently, Casp-1 activity and LDH release also decreased after ASFV infection when EP364R expression was interfered (Fig 6F and 6G). Additionally, the PI staining results showed that interference with EP364R expression reduced ASFV-induced cell death (Fig 6H). We also found that ASC oligomerization was suppressed during ASFV infection when interference EP364R expression (Fig 6I). Consistently, the mRNA levels of IL-1β, IL-6, TNF-α, and CXCL10 were also inhibited during ASFV infection when EP364R expression was knocked down (Fig 6J).

### EP364R induced pyroptosis and IL-1β production via NLRP3 inflammasome

**Fig 7:**
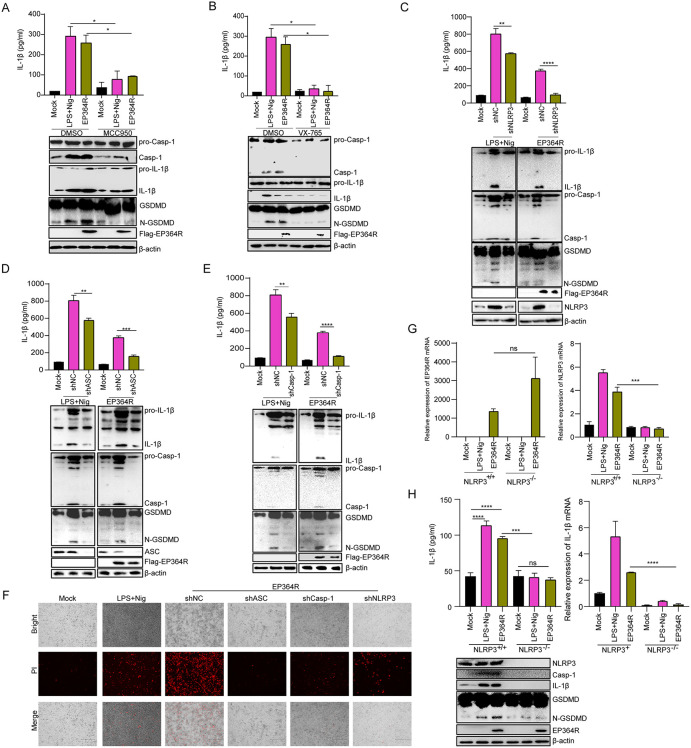
EP364R induced pyroptosis and IL-1β production via the NLRP3 inflammasome. (**A**) iPAMs were pretreated with the NLRP3 inhibitor MCC950 (10 μM) for 1 h, then transfected with 3 μg of EP364R plasmid. IL-1β secretion was measured by ELISA and expression of Casp-1, IL-1β, and GSDMD-N was analyzed by WB 24 h p.t. (**B**) iPAMs were pretreated with the Casp-1 inhibitor VX765 (10 μM) for 2 h, then transfected with 3 μg of EP364R plasmid. IL-1β secretion was measured by ELISA and expression of Casp-1, IL-1β, and GSDMD-N was analyzed by WB 24 h p.t. (**C-E**) iPAMs were transfected with 3 μg of shNLRP3 **(C)**, shASC **(D)**, or shCasp-1 (**E**) plasmids. At 72 h p.t., cells were transfected with 3 μg of EP364R plasmid. IL-1β secretion was measured by ELISA and expression of Casp-1, IL-1β, and GSDMD-N was analyzed by WB 24 h later. (**F**) iPAMs were transfected with 3 μg of shNLRP3, shASC, or shCasp-1 plasmids. At 72 h p.t., cells were transfected with 3 μg of EP364R plasmid. At 24 h p.t., cells were stained with PI (10 μl) and cell death was observed by microscopy. **(G)** Bone marrow cells were isolated from NLRP3-/- knockout mice and differentiated for 7 days in medium containing L929 cell-conditioned supernatant. Cells were then transfected with 3 μg of EP364R plasmid. Transcriptional levels of EP364R and NLRP3 were measured by qPCR 24 h p.t., showing no significant difference in EP364R transcription but confirmed absence of NLRP3. **(H)** Bone marrow-derived macrophages (BMDMs) from NLRP3-/- mice (prepared as in G) were transfected with 3 μg of EP364R plasmid. IL-1β secretion was measured by ELISA and expression of Casp-1, IL-1β, and GSDMD-N was analyzed by WB 24 h p.t. A P value less than 0.05 was considered statistically significant. *p < 0.05, **p < 0.01, ***p < 0.001.

We further determined whether EP364R induced pyroptosis through NLRP3 signaling. EP364R-expressed cells were treated with MCC950 or VX765, as shown in Fig.7A and 7B, EP364R-induced IL-1β secretion, Casp-1, and GSDMD-N maturation were suppressed during NLRP3 and Casp-1 inhibitor treatment. PI staining results showed that cell death was reduced in EP364R-expressed cells with MCC950 or VX765 treatment compared to DMSO treatment (S6A Fig).

Furthermore, IL-1β secretion, Casp-1, and GSDMD-N maturation levels were significantly reduced after knockdown of Casp-1, ASC, or NLRP3 (Fig 7C-7E). Additionally, PI staining results showed that EP364R-induced cell death decreased following disruption of ASC, NLRP3, or Casp-1 expression (Fig 7F).

We next assessed the EP364R function in NLRP3 knockout cells and found that NLRP3 mRNA expression was decreased in NLRP3 knockout BMDMs after EP364R transfection (Fig 7G). Importantly, IL-1β secretion, Casp-1, and GSDMD-N maturation were significantly suppressed during EP364R expression in NLRP3 knockout cells (Fig 7H). PI staining results also indicated that EP364R-induced cell death was decreased after NLRP3 knockout (S6B Fig).

### EP364R targets DDX3X to induce NLRP3 inflammasome activation

**Fig 8:**
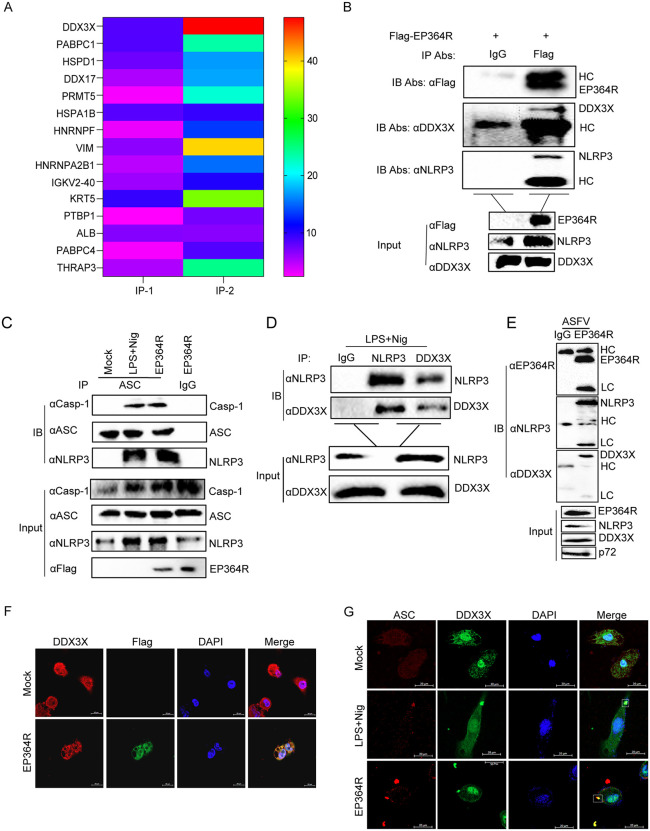
EP364R induced NLRP3 inflammasome activation by targeting DDX3X. (**A**) iPAMs and HEK293T cells were transfected with 5 μg of EP364R plasmid. Cells were lysed 24 h p.t., and immunoprecipitation (IP) samples were prepared. After protein electrophoresis and Coomassie Blue staining, target bands were excised and sent for mass spectrometry analysis. (**B**) iPAMs were transfected with 5 μg of EP364R plasmid. Cells were lysed 24 h p.t. A 100 μl aliquot of the supernatant was saved as ‘input’. The remaining supernatant was incubated with anti-Flag mouse monoclonal antibody or control IgM at 4°C for 8 h, followed by incubation with pre-washed beads for another 8 h at 4°C. Beads were washed with PBS and resuspended to prepare IP or control samples. Interaction between EP364R and DDX3X or NLRP3 was detected by WB. (**C**) iPAMs were transfected with 5 μg of EP364R plasmid. Cells were lysed 24 h p.t. A 100 μl aliquot was saved as ‘input’. The remaining lysate was incubated with anti-ASC rabbit antibody or control IgG at 4°C for 8 h, followed by incubation with beads for another 8 h at 4°C. Beads were washed and resuspended to prepare IP and control samples. NLRP3 inflammasome assembly was detected by WB. **(D)** In iPAMs lysates from LPS + Nig-induced inflammatory cells, a 100 μl aliquot was saved as ‘input’. The remaining supernatant was incubated with anti-NLRP3 rabbit antibody, anti-DDX3X mouse antibody, or control IgG at 4°C for 8 h, followed by incubation with beads for another 8 h at 4°C. Beads were washed and resuspended. Interaction between porcine DDX3X and NLRP3 was detected by WB. **(E)** PAMs were infected with ASFV, then cells were lysed 48 h post infection. A 100 μl aliquot of the supernatant was saved as ‘input’. The remaining supernatant was incubated with EP364R polyclonal antibody or control IgM at 4°C for 8 h, followed by incubation with pre-washed beads for another 8 h at 4°C. Beads were washed with PBS and resuspended to prepare IP or control samples. Interaction between EP364R and DDX3X or NLRP3 was detected by WB. **(F)** HEK293T cells were transfected with 2 μg of Flag-EP364R plasmid. At 24 h p.t., cells were fixed, and co-localization of Flag-EP364R and DDX3X was assessed by immunofluorescence assay (IFA). **(F)** HEK-293T cells were transfected with 2 μg of Flag-EP364R plasmid. At 24 h p.t., cells were fixed, and co-localization of DDX3X with ASC specks was assessed by IFA.

To analyze the mechanism of EP364R-induced cell pyroptosis, EP364R was expressed in porcine iPAMs and HEK293T and subjected to affinity purification mass spectrometry. The results showed that DDX3X interacted with EP364R (Fig 8A). The previous study reported that DDX3X was associated with NLRP3. Co-IP results showed that EP364R interacted with both porcine NLRP3 and porcine DDX3X (Fig 8B). Additionally, ASC interacted with Casp-1, NLRP3 with LPS + Nig or EP364R treatment was observed (Fig 8C), which suggested that EP364R activated NLRP3 inflammasome formation. We also found that porcine DDX3X was associated with porcine NLRP3 (Fig 8D). In addition, we also found that EP364R expressed by ASFV also interacts with DDX3X and NLRP3 (Fig 8E). Furthermore, EP364R co-localized with DDX3X was confirmed (Fig 8F). Importantly, DDX3X and ASC colocalized and formed specks after EP364R expression was observed (Fig 8G).

These results indicated that EP364R interacted with DDX3X to trigger NLRP3 inflammasome activation.

### DDX3X deficiency impaired cell pyroptosis induced by ASFV and EP364R

**Fig 9:**
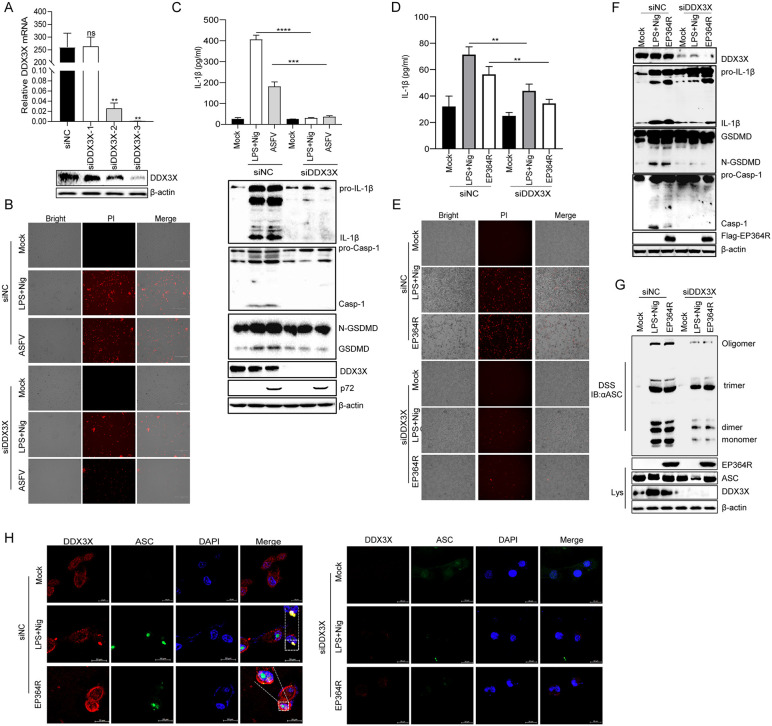
DDX3X deficiency impaired EP364R-induced pyroptosis. **(A)** HEK293T cells were transfected with small interfering RNA (siDDX3X-1, -2, or -3). At 48 h p.t., cells were transfected with 2 μg of EP364R plasmid for 24 **h.** RNA was extracted for qPCR and cells were lysed for WB analysis to screen for the most effective siDDX3X. **(B)** Porcine alveolar macrophages (PAMs) were transfected with siDDX3X-3. At 48 h p.t., cells were infected with ASFV at MOI = 1 for 24 **h.** IL-1β secretion was measured by ELISA, and expression of IL-1β and GSDMD-N was detected by WB. **(C)** PAMs were transfected with siDDX3X-3. At 48 h p.t., cells were infected with ASFV at MOI = 1 for 24 **h.** Cells were stained with PI (10 μl) and cell death was observed by fluorescence microscopy. **(D)** HEK293T cells were transfected with siDDX3X-3. At 48 h p.t., cells were transfected with 2 μg of EP364R plasmid. IL-1β secretion was measured by ELISA 24 h later. **(E)** HEK293T cells were transfected with siDDX3X-3. At 48 h p.t., cells were transfected with 2 μg of EP364R plasmid. At 24 h p.t., cells were stained with PI (10 μl) and cell death was observed by fluorescence microscopy. **(F)** HEK293T cells were transfected with siDDX3X-3. At 48 h p.t., cells were transfected with 5 μg of EP364R plasmid. Expression of IL-1β and GSDMD-N was analyzed by WB 24 h later. **(G)** HEK-293T cells were transfected with siDDX3X-3. At 48 h p.t., cells were transfected with 5 μg of EP364R plasmid. At 24 h p.t., cell lysates were centrifuged; the supernatant was the ‘input’. The pellet was resuspended in PBS, cross-linked with DSS at 37°C for 30 min, and mixed with loading buffer to prepare the ‘pellet’ sample. ASC oligomerization was detected by WB. **(H)** HEK293T cells were transfected with siDDX3X-3. At 48 h p.t., cells were transfected with 2 μg of EP364R plasmid. ASC speck formation was assessed by IFA 24 h later. And *p* value less than 0.05 was considered statistically significant. *p < 0.05, **p < 0.01, ***p < 0.001.

To investigate the role of DDX3X protein in ASFV-induced cell pyroptosis, DDX3X and EP364R were knocked down using siRNA or shRNA (Fig 9A). PI staining results showed that disrupting DDX3X expression also decreased ASFV-induced cell death (Fig 9B) We found that ASFV-induced IL-1β secretion, Casp-1, and GSDMD-N maturation were decreased after DDX3X knockdown (Fig 9C). Furthermore, as shown in. S7A and S7B Fig, co-transfection of siDDX3X and shEP364R reduced ASFV-induced IL-1β secretion, Casp-1 and GSDMD-N maturation, and cell death.

To determine the function of DDX3X on EP364R-induced cell pyroptosis, DDX3X knockdown experiments were performed. As shown in Fig 9D, DDX3X expression inhibition reduced positive control (LPS+Nigericine)- and EP364R-induced IL-1β secretion compared to the negative control siRNA (siNC). Additionally, PI staining results also showed that DDX3X expression disruption impaired EP364R-induced cell death compared to siNC (Fig 9E). GSDMD-N and Casp-1 maturation also reduced after EP364R expression in DDX3X knockdown cells (Fig 9F). Similarly, DDX3X knockdown decreased EP364R-induced ASC oligomerization (Fig 9G). Importantly, EP364R-induced ASC specks were obviously suppressed after DDX3X knockdown (Fig 9H).

These results suggested that DDX3X was involved in EP364R-induced pyroptosis through NLRP3 inflammasome activation.

### DDX3X acted as a bridge for EP364R-induced NLRP3-mediated pyroptosis

**Fig 10:**
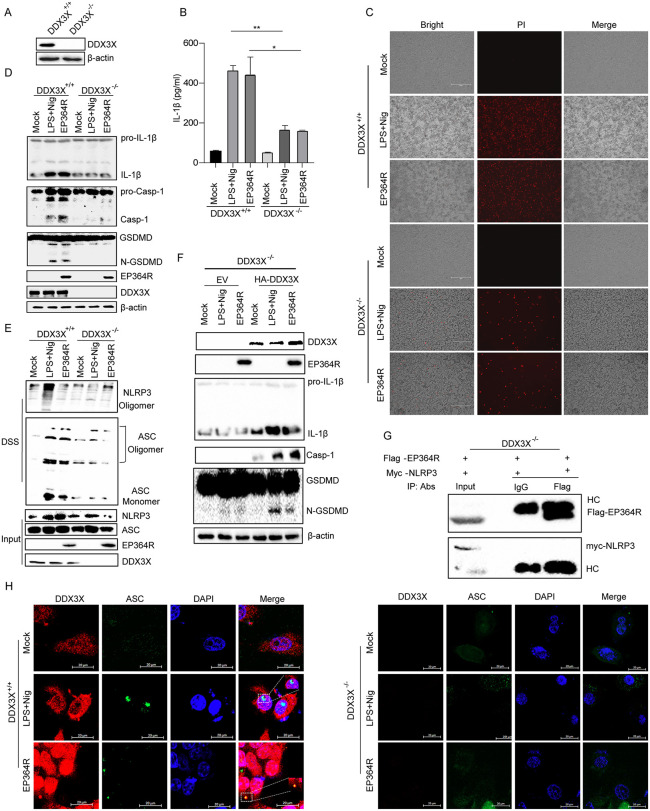
DDX3X acted as a bridge for EP364R-induced NLRP3-mediated pyroptosis. **(A)** WB analysis of lysates from DDX3X knockout cells. **(B)** DDX3X knockout cells were transfected with 2 μg of EP364R plasmid. IL-1β secretion was measured by ELISA 24 h p.t. **(C)** DDX3X knockout cells were transfected with 5 μg of EP364R plasmid. At 24 h p.t., cells were stained with PI (10 μl) and cell death was observed by fluorescence microscopy. **(D)** DDX3X knockout cells were transfected with 5 μg of EP364R plasmid. Expression of IL-1β and GSDMD-N was detected by WB 24 h p.t. **(E)** DDX3X knockout cells were transfected with 5 μg of EP364R plasmid. At 24 h p.t., cell lysates were centrifuged; the supernatant was the ‘input’. The pellet was resuspended in PBS, cross-linked with DSS at 37°C for 30 min, and mixed with loading buffer to prepare the ‘pellet’ sample. ASC and NLRP3 oligomerization were detected by WB. **(F)** DDX3X knockout cells were co-transfected with 5 μg each of EP364R and DDX3X plasmids. Expression of IL-1β and GSDMD-N was detected by WB 24 h p.t. **(G)** DDX3X knockout cells were co-transfected with 5 μg each of EP364R and NLRP3 plasmids. At 24 h p.t., cells were lysed. A 100 μl aliquot was saved as ‘input’. The remaining lysate was incubated with anti-Flag mouse antibody or control IgM at 4°C for 8 h, followed by incubation with beads for another 8 h at 4°C. Beads were washed and resuspended. Interaction between NLRP3 and EP364R was detected by WB. **(H)** DDX3X knockout cells were transfected with 2 μg of EP364R plasmid. ASC speck formation was observed by IFA 24 h p.t. And *p* value less than 0.05 was considered statistically significant. *p < 0.05, **p < 0.01, ***p < 0.001.

Next, the effect of DDX3X knockout on EP364R-induced cell pyroptosis was assessed. As shown in Fig 10A, DDX3X was successfully knocked out in HEK293T cells. IL-1β secretion was reduced in DDX3X knockout cells when EP364R expression (Fig 10B). PI staining results showed that EP364R-induced cell death was decreased in DDX3X knockout cells (Fig. 10C). Casp-1 and GSDMD-N maturation were reduced without DDX3X expression (Fig 10D). ASC oligomerization was reduced in DDX3X knockout cells with EP364R expression (Fig 10E). Furthermore, DDX3X expression was recovered in knockout cells, and EP364R-induced maturation of Casp-1 and GSDMD-N was restored (Fig 10F). In addition, IP staining results showed that the interaction of EP364R and NLRP3 was absent in DDX3X knockout cells (Fig 10G). Meanwhile, EP364R-induced ASC speck formation was abolished after DDX3X knockout (Fig 10H).

Taken together, these results suggested that DDX3X served as a bridge for EP364R-induced pyroptosis.

### EP364R-induced cell pyroptosis without utilizing DDX3X helicase activity

Based on the results of the aggregation of DDX3X with NLRP3 and forming speckles were modulated by EP364R. Additionally, DDX3X possesses RNA helicase activity. We investigated whether the helicase activity of DDX3X was involved in EP364R-induced cell pyroptosis. We found that RK-33 (an inhibitor of helicase activity) did not affect EP364R-induced IL-1β secretion (S8A-S8B Fig). Furthermore, RK-33 failed to suppress EP364R-induced Casp-1 and GSDMD-N maturation (S8C Fig). The formation of EP364R-induced ASC oligomerization also remained unchanged after RK-33 treatment (S8D Fig). The fluorescence results showed that EP364R-induced ASC speckles formation remained unchanged with RK-33 treatment (S8E Fig). Moreover, EP364R-induced cell death caused by EP364R did not decrease with RK-33 treatment (S8F Fig). These results suggested that EP364R-triggered pyroptosis was independent of DDX3X helicase activity.

### The mode of EP364R-DDX3X interaction

**Fig 11:**
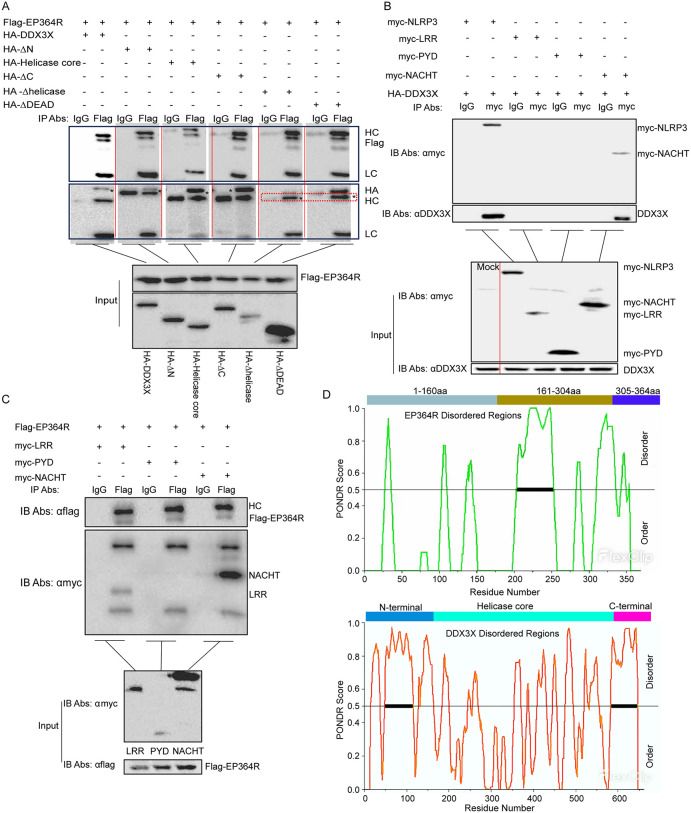
Interaction mode between EP364R and DDX3X. **(A)** HEK293T cells were co-transfected with 5 μg of EP364R plasmid and 5 μg of plasmids encoding different HA-tagged DDX3X domains (HA-DDX3X, HA-ΔN, HA-Helicase core, HA-ΔC, HA-ΔDEAD, HA-ΔHelicase). Cells were lysed 24 h p.t. A 100 μl lysed samples were detected as ‘input’. The remaining lysate was incubated with anti-Flag mouse antibody or control IgM at 4°C for 8 h, followed by incubation with beads for another 8 h at 4°C. Beads were washed and subjected to WB. **(B)** HEK293T cells were co-transfected with 5 μg of HA-DDX3X plasmid and 5 μg of plasmids encoding different myc-tagged NLRP3 domains (myc-LRR, myc-PYD, myc-NACHT). Cells were lysed 24 h p.t. A 100 μl lysed samples were detected as ‘input’. The remaining lysate was incubated with anti-Flag antibody (presumably targeting the tag on DDX3X or co-precipitating partner) or control IgM at 4°C for 8 h, followed by incubation with beads. Beads were washed and resuspended. Interaction between NLRP3 domains and DDX3X was detected by WB. **(C)** HEK293T cells were co-transfected with 5 μg of EP364R plasmid and 5 μg of plasmids encoding different myc-tagged NLRP3 domains (myc-LRR, myc-PYD, myc-NACHT). Cells were lysed 24 h p.t. A 100 μl lysed samples were detected as ‘input’. The remaining lysate was incubated with anti-Flag mouse antibody or control IgM at 4°C for 8 h, followed by incubation with beads. Beads were washed and resuspended. Interaction between NLRP3 domains and EP364R was detected by WB. **(D)** Intrinsically disordered regions in porcine DDX3X and porcine NLRP3 proteins were predicted using the PONDR software.

To identify the critical sites of EP364R-DDX3X interaction, Co-IP assays and protein molecular docking model were employed. Co-IP of the truncated DDX3X domain with EP364R was performed to identify the functional domains of DDX3X, the locations of the different domains of DDX3X: N-terminal (1–159aa), DEAD domain (160–410aa), Helicase (414–575aa), C-terminal (576–662aa), Helicase core (160–575aa) (S9A Fig). As shown in Fig 11A, EP364R interacted with all the DDX3X domain deletion mutants. We then found that the NACHT domain of porcine NLRP3 interacted with porcine DDX3X, which was similar to the results of the binding domains of human NLRP3 and human DDX3X [37] (Fig 11B). In addition, Co-IP results showed that EP364R interacted with the LRR and NACHT domains of NLRP3(Fig 11C).

To further define the binding interface, we conducted computational protein-protein docking using AlphaFold 3.0 for homology modeling. Neither EP364R nor porcine DDX3X has experimentally resolved crystal structures, so we generated AlphaFold-predicted models with confidence scores (S9B Fig). Due to low confidence scores in EP364R’s 305–366aa region, this segment was omitted from analysis. Docking simulations focused on EP364R’s nuclease domain (1–160aa), DNA-binding domain (161–304aa), and full-length structure in complex with DDX3X (S9B Fig). Key interaction patterns emerged from these simulations (S9C Fig):

Pose 1: Hydrogen bonds formed between EP364R residues Lys174, Met125, Val126, and Lys252 with DDX3X residues Asp165, Val561, Lys418, Asp426, and Tyr200, respectively.

Pose 2: Specific ionic interactions involved EP364R residues Lys259, Arg226, and Lys252 binding to DDX3X residues Asp433, Asp558, and Asp426, respectively.

Pose 3: Multimodal interactions were observed between EP364R residues Gln30, Lys154, Lys50, and Met156 and DDX3X residues Glu388, Asp521, Glu523, Glu449, Pro519, and Arg528.

The protein docking results showed that the binding sites of EP364R and DDX3X were located in different regions. We speculated that this was related to their spatial conformation. The previous study reported that DDX3X has disordered regions [37]. Therefore, we predicted the disordered regions of EP364R and porcine DDX3X and found that many sites where EP364R and DDX3X molecules dock were located within these disordered regions (Fig 11D).

These findings showed that both domains of EP364R mediated interactions with the spatial structure of DDX3X.

### Natural product HAMNO targets EP364R to inhibit ASFV replication

Currently, there are no commercial vaccines or drugs available to prevent or treat ASF. We speculated that drugs may inhibit ASFV replication. Given the findings of the EP364R mode, we sought to screen natural products targeting EP364R. The EP364R mode was analyzed using MOE software. Inhibitor binding active site 1 including Phe3, Val5, Lys39, Gln40, Leu41, Phe42, Thr43, Leu47, Cys49, Ser53, Ile55, and Leu85, and site 2 including Glu256, Ile257, Leu258, Lys259, Asp260, His261, Ile277, Gln278, Ile279, and Val280, were found on the surface of EP364R (S10A Fig). Then, using Schrödinger software to perform compound docking on each compound in the chemical library (consisting of 23619 small molecules). Ultimately, 73 and 61 small molecules that bind to site 1 and site 2, respectively, were retained through affinity and structural diversity analysis, as well as visual binding mode analysis.

Further, cells treated with these retained small molecules separately were infected with ASFV-GFP. A natural product, HAMNO, was found to strike a balance between inhibiting ASFV replication and producing less cytotoxicity (S10B-S10D Fig). The binding location of HAMNO in the predicted structure of EP364R was analyzed to bind site 2 (S10E Fig). Partial views of the 3D and 2D conformations of HAMNO binding to EP364R were performed (S10F and S10G Fig), and the amino acids within 4Å surrounding the HAMNO were annotated. For example, HAMNO forms hydrogen bonds with E256, K259, and D260 in the structure of EP364R (S10G Fig). Meanwhile, the inhibition effect of HAMNO on ASFV replication was dose-dependent (S10H and S10I Fig). Additionally, cells treated with HAMNO at indicated times were infected with ASFV, and we found that it was more effective when cells were treated with HAMNO before ASFV infection (S10J and S10K Fig.).

To investigate HAMNO has the function on ASFV or EP364R-induced pyroptosis and inflammation response, HAMNO was added to the cells accompany with ASFV or EP364R treatment. The results demonstrated that HAMNO decreases the IL-1β secretion and pyroptosis biomarkers (N-GSDMD, Casp-1, IL-1β) expression which induced by ASFV and EP364R (S10L and S10M Fig.). Moreover, cytokines of IL-1β, IL-18, and TNF-α transcription were suppressed during ASFV infection or EP364R transfection with HAMNO treatment (S10N Fig). Importantly, HAMNO treatment also inhibited the interaction of EP364R, NLRP3, and DDX3X (S10O Fig). Taken together, these findings suggested that HAMNO was a potential therapeutic agent that effectively inhibited ASFV replication via EP364R.

## Discussion

ASF causes substantial economic losses in the global pig industry. To date, the effective vaccines or antiviral drugs are not available to control ASF. While acute inflammation represents a crucial host defense against injury or infection, excessive inflammation can lead to cell death, tissue damage, and mortality. ASFV infection causes a hemorrhagic fever characterized by high fever, cutaneous hemorrhages, cyanosis of extremities, and acute death. Tissue damage and organ dysfunction in pigs infected with ASFV may be correlated with a cytokines storm. Although previous studies reported that rapid elevation of cytokines levels occurred in ASFV-infected swine, the underlying mechanisms remain poorly defined. Therefore, elucidating the mechanistic link between ASFV infection and inflammation is crucial to understanding why ASFV causes tissue damage and acute mortality.

This study demonstrated that ASFV infected BMDMs or PBMCs and triggered robust expression of cytokines and chemokines, concomitant with the induction of pyroptosis. The ASFV-encoded proteins were examined and EP364R was identified as a driver of cytokines production and pyroptosis by activating the NLRP3 inflammasome. When NLRP3 was knocked down or deleted, the pyroptosis-inducing capacity of EP364R was notably diminished. EP364R expression induced splenomegaly in mice and exacerbated systemic cytokines release. Mechanistically, EP364R recruited DDX3X, facilitating its role as a scaffold for NLRP3 inflammasome assembly and activation. When DDX3X was knocked out, ASFV- and EP364R-induced pyroptosis, inflammatory cytokines release, and danger signal (such as ATP, CRP) release were significantly reduced, along with the oligomerization and enrichment of the NLRP3 inflammasome. However, when DDX3X was restored, the function of EP364R was restored. The interaction of EP364R and NLRP3 was also abolished when DDX3X was knocked out. The Co-IP results confirmed that the NACHT domain of porcine NLRP3 interacted with porcine DDX3X. EP364R interacted with the NACHT and LRR domains of NLRP3, and EP364R bound to all domains of DDX3X. Structural modeling showed direct binding between EP364R and DDX3X, facilitated by complementary surface characteristics. Modeling also indicated that EP364R interacted with DDX3X at multiple sites across different domains. Furthermore, a small-molecule HAMNO targeting EP364R was identified as an inhibitor of ASFV replication through screening of a small-molecule library. Collectively, our findings delineated a novel mechanism of ASFV EP364R protein-induced pyroptosis and inflammation through NLRP3 inflammasome activation. This study identified a promising therapeutic candidate and provided crucial insights for the development of drugs against ASFV infection.

Inflammatory cell death (apoptosis, pyroptosis, and necroptosis) is a key factor in triggering cytokines storms [38–40]. Inflammatory cell death has been documented with diverse viral infections [41–43]. *In vivo*, the gastrohepatic lymph nodes undergo extensive lymphocyte depletion following ASFV infection [44]. In Malawi Lil-20/1 ASFV strain-infected pigs, splenic marginal zone B cells exhibited structural disorganization and cell apoptosis [45]. The ASFV pMGF360-16R induced apoptosis by competing with BAX for binding to HSP60 [46]. Additionally, ASFV exploited apoptotic bodies for viral dissemination [47]. Zhang et al., demonstrated that ASFV infection triggered ZBP1-RIPK3-mediated necroptosis, which was readily detectable both *in vivo* and *in vitro* and potently drove immunopathological cell death [48]. Given that the three forms of inflammatory cell death are interconvertible, the observation of both apoptosis and necroptosis in the aforementioned study suggests that substantial pyroptosis is also likely to occur. This has been confirmed in this study and other studies [29,31]. However, research on ASFV-induced pyroptosis remains scarce, with a predominant focus on inhibiting pyroptosis [35]. Previous study showed that ASFV induced low level of pro-inflammatory cytokines mRNA expression [30]. We infected porcine alveolar macrophages (PAMs) with African swine fever virus (ASFV) and observed similar results. This initially raised the question of whether ASFV elicits a robust inflammatory response. However, the late stages of ASFV infection in animals are characterized by a dramatic increase in cytokine secretion. Therefore, these findings indicate that ASFV induces cytokine expression and secretion primarily during the later phase of infection. Furthermore, ASFV likely promotes inflammatory responses through a combined effect of stimulating multiple immune cell types, thereby amplifying the overall immune reaction. Therefore, we first infected bone marrow-derived macrophages (BMDMs) with ASFV and observed that proinflammatory cytokines and pyroptosis occurred in BMDMs after ASFV infection, as indicated by DAMPs release, typical cellular appearance, pyroptosis markers, and strong cytokines secretion. Moreover, ASFV infection elicited a stronger cytokine response in bone marrow-derived macrophages (BMDMs) than in porcine alveolar macrophages (PAMs). This suggests that ASFV-induced cytokine production and pyroptosis may occur in distinct immune cell populations. This also suggests that pyroptosis also constitutes a key mechanism for inducing inflammation and tissue damage in domestic swine infected with ASFV. Notably, our further findings indicated that ASFV infected PBMCs and elicited a more robust cytokines secretion profile. Consequently, we utilized PBMCs to investigate ASFV-induced pyroptosis. We found that ASFV infected these mixed immune cells with ASFV and triggered pyroptosis and significantly elevated cytokines production. These results further demonstrated that ASFV infection of heterogeneous cell populations may be involved in enhancing intercellular communication and promoting inflammatory responses.

ASFV, belonging to the family *Asfarviridae*, possesses a large double-stranded DNA genome. AIM2 serves as a key inflammasome component by sensing microbial double-stranded DNA in the cytosol to induce cell pyroptosis; however, its functions as a pseudogene in swine (*Sus scrofa*). The previous study has shown that NLRP3 functionally replaced AIM2 as the DNA inflammasome sensor in human myeloid cells [49]. The NLRP3 inflammasome, a key component of the innate immune system, plays a critical role in sensing cellular damage and pathogenic infections [50,51]. Consequently, we focused on whether ASFV infection triggered NLRP3-mediated pyroptosis and inflammation broadly. To address this issue, we employed inflammasome inhibitors and shRNA-mediated knockdown to confirm that ASFV infection could activate the NLRP3 inflammasome.

The regulation of inflammatory responses involves multiple classical signaling pathways such as NF-κB, MAPK, JAK-STAT, and Casp-1 [52–55]. Previous studies have demonstrated that ASFV could induce phosphorylation of NF-κB [56]. Wu et al., revealed that the ASFV protein I177L enhanced NF-κB activation by promoting TRAF6 ubiquitination, thereby increasing TAK1 activity and leading to elevated expression of inflammatory cytokines [31]. Yang Chen et al., further confirmed that dihydromyricetin suppresses ASFV-induced inflammatory responses by modulating the MAPK–NF-κB signaling pathway [57]. The functional activation of many cytokines depends on the JAK-STAT signaling pathway. However, existing research predominantly describes the inhibitory effect of ASFV on NF-κB, MAPK, and JAK-STAT signaling pathway [58–60]. We therefore hypothesize that in ASFV-infected cells, JAK-STAT signaling is suppressed, and the triggered inflammatory response may instead originate from non-infected cells. These cells are likely activated by cytokines or danger signals released from infected cells undergoing inflammatory cell death. In the present study, we found that EP364R activates NF-κB signaling, which serves as the priming signal for inflammasome activation. This finding further supports the ability of EP364R alone to activate the Casp-1-meidated pyroptosis signaling. However, the precise mechanism by which EP364R regulates NF-κB remains to be fully elucidated.

Our previous study demonstrated that the Seneca Valley virus 3D protein activated the NLRP3 inflammasome and triggered inflammatory responses in swine [61]. The M protein of Dengue virus induced tissue damage and vascular leakage in mice through NLRP3 inflammasome activation [62]. Zika virus-encoded NS5 protein promoted viral entry into the brain and exacerbated mortality in mice, also via NLRP3 inflammasome activation [63]. ASFV possesses a large genome encoding over 150 proteins, the majority of which have unknown functions. Although the ASFV MGF505-7R protein was reported to maintain the function to inhibit IL-1β production, the evidence of ASFV-mediated activation of the NLRP3 inflammasome and induction of IL-1β was not provided.

Given that ASFV elicits inflammatory responses and viruses always utilize their own proteins to induce cell death and inflammation, we screened all known ASFV-encoded proteins. We identified EP364R as a candidate to induce pyroptosis and inflammatory activity. ASFV infects domestic pigs, and cytokines are sharply upregulated in the late stage of infection. This may be because such proteins induce a significant amount of cell death in the late stage, leading to the release of cytokines and danger signals, and ultimately inducing a cytokines storm. Our experiments and other studies have shown that EP364R was a late-expressed protein. We further demonstrated that EP364R promoted pyroptosis and the release of cytokines and DAMPs. Notably, EP364R-induced pyroptosis was nearly equivalent to the positive control, suggesting that this protein likely plays a crucial role in triggering pyroptosis and inflammatory responses. Although a recent study identified a 2’,3’-cGAMP binding motif within EP364R and implicated the protein in inhibiting IFN-mediated responses [64], Yutao Wang et al., found that once the inflammasome was activated, Casp-1 interacted and cleaved cyclic guanylate cyclase-adenylate (cGAMP) synthase (cGAS) to inhibit cGAS-dependent immune response [65]. We speculated that EP364R may also activated Casp-1, which cleaves cGAS and subsequently inhibits the innate immune response, thereby facilitating immune evasion. This requires further study to confirm. Furthermore, shRNA targeting EP364R was employed, and the results showed that ASFV-induced pyroptosis and the release of cytokines and DAMPs were significantly reduced, suggesting that EP364R was an important factor, but not the only one, in inducing pyroptosis and inflammatory cytokines production. Other proteins may also be involved in this process. We have screened and identified several additional proteins and are currently investigating the underlying mechanisms. We also attempted to generate an EP364R knockout virus using a homologous recombinant plasmid; however, the knockout virus disappeared after 2–3 passages, and no EP364R-deficient strain was purified. Further EP364R function verification will be conducted using other methods, such as conditional knockout or point mutation of the recombinant ASFV strain. Many biological processes exhibit dual functionality. EP364R promotes the maturation of N-GSDMD, whereas ASFV pS273R cleaves GSDMD into non-pyroptotic fragments. This duality may reflect a finely tuned mechanism by which ASFV balances viral replication and host inflammatory responses during infection. Interestingly, both proteins are late-expressed viral factors, suggesting potential synergism between them. For instance, during active viral replication and assembly, pS273R may suppress pyroptosis to facilitate these processes. Once replication and assembly are complete, EP364R could then promote pyroptosis to aid rapid viral dissemination. Alternatively, the specific GSDMD fragments generated by pS273R cleavage might possess uncharacterized functions. Further investigation is required to elucidate these mechanisms [33].

The excessive release of cytokines and DAMPs represents one of the major contributors to tissue damage. For instance, Dengue virus infection promotes vascular leakage in mice through the production of cytokines. Similarly, SARS-CoV-2 infection induces significantly elevated cytokines levels in patient serum and bronchoalveolar lavage fluid. Our results demonstrated that the EP364R protein elevated cytokines levels in the serum of mice. Notably, EP364R expression induced splenomegaly in a mouse model. Given that splenomegaly is a hallmark clinical manifestation of ASFV infection, these findings suggested that the ASFV EP364R protein might contribute to splenic damage through pyroptosis and pro-inflammatory cytokines production. Consistently, pyroptosis induced by EP364R was significantly attenuated in BMDMs of NLRP3^−/−^ mice.

Activation of the NLRP3 inflammasome initiates with NEK7 binding to deubiquitinated and self-aggregated NLRP3. This complex subsequently recruits the adaptor protein ASC, leading to the formation of large macromolecular assemblies termed ASC specks. Within these specks, ASC engages pro-Casp-1 via CARD-CARD interactions, facilitating proximity-induced autocleavage and activation of Casp-1. The active Casp-1 then proteolytically processes pro-IL-1β and pro-IL-18 into their bioactive forms, IL-1β and IL-18 [66]. Co-IP and IFA results demonstrated that the ASFV EP364R protein interacted with NLRP3 and promoted ASC speck aggregation. However, direct binding assays using purified EP364R and NLRP3 proteins yielded negative results. NLRP3 inflammasome activation responds to diverse stimuli, such as perturbations in ion flux and lysosomal destabilization, and currently lacks a definitive classification scheme. Previous study established that the stress granule protein DDX3X interacted with NLRP3 to drive inflammasome activation. Macrophages utilize DDX3X to interpret stress signals, determining the cellular choice between pro-survival stress granule formation and pyroptotic ASC speck assembly [37]. Therefore, we employed EP364R as bait in immunoprecipitation coupled with IP-MS, revealing a specific interaction between EP364R and DDX3X. To validate this interaction, we generated DDX3X-knockout cells. In this context, the interaction of EP364R and NLRP3 was abrogated, indicating that DDX3X served as an essential molecular bridge for EP364R-mediated NLRP3 inflammasome activation. Further immunofluorescence microscopy confirmed that EP364R expression accelerated ASC speck formation and oligomerization. Crucially, this acceleration was abolished upon deletion of DDX3X. Similar to the previous study, the NACHT domain of porcine NLRP3 interacted with porcine DDX3X. These findings suggested that EP364R might promote the release of DDX3X from stress granules, thereby facilitating its recruitment and contributing to pyroptosis. This hypothesis merits further investigation.

DDX3X is involved in various disease processes, including virus infection, inflammation, and cancer [67]. DDX3X contains a highly conserved helicase core. To determine whether RK-33 participates in the inflammatory response and pyroptosis as a helicase, the enzyme was employed, and the results showed that inhibition of the active site did not lead to the loss of pyroptosis, indicating that the function of DDX3X in inducing pyroptosis is independent of helicase activity. Porcine DDX3X interacts with NACHT, which is essential for NLRP3 self-association, suggesting that DDX3X activates NLRP3 oligomerization. EP364R binds to NACHT and LRR of NLRP3 through DDX3X, may further activate NLRP3 oligomerization and induce the release of LRR inhibition of NLRP3 self-function. To elucidate how EP364R activates the NLRP3 inflammasome via DDX3X, Co-IP assays demonstrated that EP364R bound to multiple domains of porcine DDX3X. To further investigate this interaction, we performed structural modeling of EP364R and porcine DDX3X. The modeling revealed that EP364R engaged multiple surface sites on DDX3X, corroborating the Co-IP results and indicating that complementary structural surfaces likely mediate the interaction. By predicting the disordered regions of EP364R and DDX3X, we found that a significant portion of the binding sites for protein docking was located in the disordered regions.

The large and complex genome of ASFV complicates the identification of effective antigens. Furthermore, vaccine development is hampered by the risk of environmental dissemination associated with live-attenuated vaccines. Consequently, small-molecule therapeutics represent a promising alternative approach for the prevention and treatment of ASF. Given that EP364R induced robust pyroptosis and cytokine release, we screened a small-molecule library targeting EP364R to identify inhibitors of ASFV replication. Among these screened molecules, HAMNO exhibited potent inhibition of ASFV replication with minimal cytotoxicity. Whereas HAMNO has been widely studied in the context of cancer therapy, relatively little attention has been given to its potential function for the inhibition of viral replication. By specifically binding to the DNA-binding domain of the RPA70 subunit and competitively inhibiting RPA’s association with single-stranded DNA, HAMNO causes the accumulation of extensive unprotected single-stranded DNA during replication, which markedly increases replication stress and severely compromises DNA damage repair, and HAMNO dramatically decreases vaccinia DNA replication [68–73]. This molecule was first found to target specific residues on EP364R. EP364R possesses an ERCC4-like region, and the site 1 bound by HAMNO is also located in this region. Based on the function of HAMNO, it may inhibit the functional domain of EP364R, thereby modulating viral replication. The results also demonstrated that HAMNO also inhibits ASFV and EP364R function on pyoptosis and inflammation response. The specific mechanism needs further verification. Due to cost constraints, the *in vivo* function of HAMNO was not validated in swine. The synthesis cost challenges and *in vivo* validation will be addressed in future studies.

**Fig 12:**
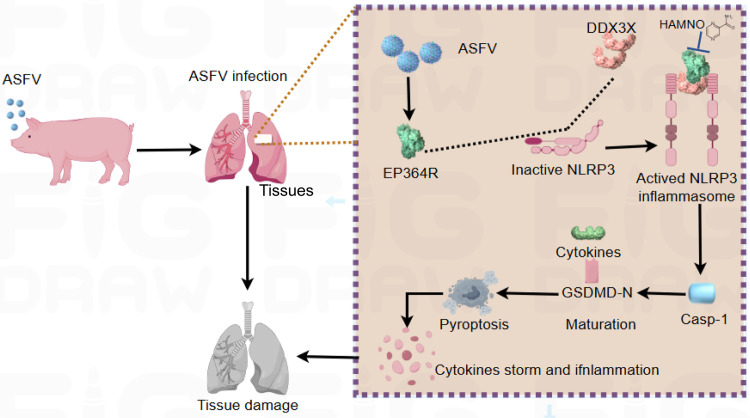
Mechanistic schematic of EP364R induced pyroptosis. Upon infection of domestic swine tissues, the ASFV-encoded protein EP364R binds to a specific spatial structure of DDX3X, which triggers the oligomerization of the NLRP3 inflammasome. This activation leads to the cleavage and activation of Casp-1, which in turn executes pyroptotic cell death and facilitates the release of pro‑inflammatory cytokines and damage‑associated molecular patterns (DAMPs). This cascade is likely a key driver of the cytokine storm‑mediate tissues damage during infection. HAMNO, a small molecule targeting EP364R, inhibits ASFV replication through disruption of the EP364R-DDX3X interaction.

Collectively, we establish a model whereby ASFV, upon infection, utilizes its encoded EP364R protein to engage DDX3X, thereby activating NLRP3 inflammasome assembly. This pathway drives pyroptosis and a massive cytokine release, contributing to splenomegaly and culminating in tissue damage orchestrated by a cytokine storm. Blocking EP364R using HAMNO inhibited ASFV replication, indicating EP364R was a promising therapeutic target for ASF (Fig 12).

### Materials and methods

### Ethics statement

This study complies with local laws and institutional requirements. The animal study was approved by the Ethic Committee of Lanzhou Veterinary Research Institute, Chinese Academy of Agricultural Sciences (License No. LVRIAEC-2022–053).

### Viruses and cells

The ASFV CN/GS/2018 strain was identified and preserved by the ASF Regional Laboratory of the Lanzhou Veterinary Research Institute, Chinese Academy of Agricultural Sciences. The ASFV-GFP strain was preserved by the Lanzhou Veterinary Research Institute. The *E. coli* DH5α strain was purchased from Takara Bio Inc. Primary porcine alveolar macrophages (PAMs) were isolated from 30–40-day-old SPF piglets in our laboratory. HEK293T cells and iPAMs (3D4/21 cell line) were preserved in our laboratory. DDX3X gene knockout HEK293T cells were purchased from Beyotime. NLRP3 gene knockout mice were bred in our laboratory. All the cells are cultured with RPMI 1640 or DMEM (Gibco, USA) with 10% fetal bovine serum (FBS) (Aoqing Biotechnology Co.,LTD, Beijing, China).

### Reagents, antibodies

Lipopolysaccharide (LPS), nigericin, MCC950, VX-765, DSS Crosslinker, and Protein A/G Magnetic Beads were purchased from MCE. RK-33 was purchased from TargetMol. Lipofectamine 2000, Opti-MEM, and Plasmocin treatment were purchased from Invitrogen. The one-step TB Green RT-PCR quantitative detection kit was purchased from Takara. Protease inhibitor PMSF and lactate dehydrogenase (LDH) activity detection kit were purchased from Solarbio. The NucleoBond ultra-rapid transfection-grade plasmid midi-prep kit Plus was purchased from MN (Macherey-Nagel). The Casp-1 activity detection kit and ATP detection kit were purchased from Beyotime. The CCK-8 kit and total RNA extraction reagent were purchased from Biosharp. Propidium iodide (PI) staining solution was purchased from BD Pharmingen. Dimethyl sulfoxide (DMSO) was purchased from Sigma-Aldrich. The cytokines ELISA detection kit was purchased from Ximbio. The lentiviral infection enhancer polybrene was purchased from Hanbio Biotechnology. HA-Tag (26D11) mAb, Myc-Tag (19C2) mAb, and DDX3X antibody were purchased from Abmart. Monoclonal anti-FLAG M2 mouse antibody and anti-beta-actin mouse monoclonal antibody were purchased from Sigma-Aldrich. Horseradish peroxidase (HRP)-labeled goat anti-rabbit IgG (H + L) and HRP-labeled goat anti-mouse IgG (H + L) were purchased from ZSGB-BIO. IL-1β (D3U3E) Rabbit mAb, Cleaved-IL-1β (Asp116) (D3A3Z) Rabbit mAb, Casp-1 (D7F10) Rabbit mAb, Cleaved Casp-1 (Asp297) (D57A2) Rabbit mAb, Gasdermin D (E9S1X) Rabbit mAb, Cleaved Gasdermin D (Asp276) Crosslinker, and fluorescent secondary antibodies were purchased from CST. Anti-ASFV-p72 and Anti-ASFV-p30 antibidies were prepared and stored in our laboratory.

### Plasmids and transfection

Eukaryotic expression plasmids Flag-EP364R, Myc-NLRP3, Myc-LRR, Myc-PYD, and Myc-NACHT were constructed and preserved in our laboratory. Eukaryotic expression plasmids HA-DDX3X, HA-ΔN, HA-Helicase core, HA-ΔC, HA-ΔDEAD, and HA-ΔHelicase were synthesized by Sangon Biotech. Lentiviral overexpression plasmids pHBLV-Vector and pHBLV-EP364R, as well as lentiviral packaging accessory plasmids psPAX2 and pMD2.G, were purchased from HANBIO. HEK293T cells were cultured in DMEM complete medium, while iPAMs cells and PAMs were cultured in 1640 complete medium at 37°C with 5% CO₂. When cell density reached 70%, transfection was performed. The plasmids to be transfected and Lipofectamine 2000 were separately mixed with Opti-MEM medium, and incubated at room temperature for 5 min, then plasmids and Lipofectamine 2000 were mixed and incubated at room temperature for another 15 min followed by added to the cells. The medium was replaced with complete medium at 5h post incubation at 37°C with 5% CO₂, and the cells were collected at 24 h post transfection.

### Gene screen assays

Plasmids encoding NLRP3, pro-Caspase-1, pro-IL-1β and ASC were co-transfected with either ASFV gene-encoding plasmids or an empty vector (EV) into iPAM or PK-15 cells. After 24 h, the supernatants were collected and lyophilized. The resulting powder was reconstituted in PBS, and the concentration of porcine IL-1β was measured using a commercial ELISA kit according to the manufacturer’s instructions. The results were compared with those obtained from supernatants of cells transfected with the EV.

### Co-IP and western blotting analysis

HEK293T cells were transfected with target plasmids, lysed, and centrifuged. Input samples were prepared by boiling lysates with loading buffer. For Co-IP, lysates were incubated with target antibodies or control IgG overnight at 4°C, followed by Protein A/G Magnetic Beads for another 8 h. Beads were washed, boiled with loading buffer, and analyzed by Western Blotting (WB)

### RNA isolation and qPCR

Trizol lysis buffer was added to ASFV CN/GS/2018-infected BMDMs and lyse at room temperature for 10 min. Add chloroform, vortex vigorously for 15 s, let stand for 15 min, and centrifuge at 12000 r/min for 20 min at 4°C. Carefully transfer the upper aqueous phase to a new EP tube, add pre-cooled isopropanol, mix, and let stand at room temperature for 30 min, then centrifuge at 12000 r/min for 15 min at 4°C and discard the supernatant, wash with pre-cooled 75% ethanol, and centrifuge at 12000 r/min for 15 min at 4°C. Discard the supernatant, air-dry the ethanol, dissolve the RNA in DEPC water and store at −80°C or proceed directly to RT-qPCR using the one-step amplification kit. For absolute quantification, use cDNA as the template for RT-qPCR. One-step RT-qPCR amplification kit was employed to amplify the targeting genes. For absolute quantification, the total 20 µL volume of reaction system include: 2 µL template, 0.5 µL probe, 1 µL for each primer (10 µM), 10 µL mix buffer, and 5.5 µL ddH₂O. The reaction program was set as: 94°C for 30 s; 40 cycles of 94°C for 5 s, 60°C for 30 s; 95°C for 15 s, 60°C for 1 min, 95°C for 1 s. For relative quantification, reaction system include: 200 µL template, 0.4 µL for each primer (10 µM), 0.4 µL enzyme, 5 µL 2X Mix, 2.8 µL ddH₂O. The reaction program set as: 95°C for 30 s; 40 cycles of 95°C for 5 s, 60°C for 30 s.

The sequences of primers and probes used in this study are listed in Table 1.

**Table 1:** Primers for qPCR.

Quantitative qPCR primer and probe	Sequence (5’-3’)
ASFV-P72-F	CTGCTCATGGTATCAATCTTATCGA
ASFV-P72-R	GATACCACAAGATCAGCCGT
ASFV-probe	FAM-CCACGGGAGGAATACCAACCCAGTG-TAMRA
**qPCR primer**	**Sequence (5’-3’)**
ASFV-P72-qPCR-F	ATGAAATGATGCCATCCCATTG
ASFV-P72-qPCR-R	CATTCAAGTGCTGTCTGACATG
EP364R-qPCR-F	TCTAAGGAAATGGTGTGCGTCGTTC
EP364R-qPCR-R	GCTACGGAACAAGTGTTTGCCAAG
p-GAPDH-qRT-F	ACATGGCCTCCAAGGAGTAAGA
p-GAPDH-qRT-R	GATCGAGTTGGGGCTGTGACT
p-IL-1β-qRT-F	GACGGGCTTTTGTTCTGCTT
p-IL-1β-qRT-R	GGACATGGAGAAGCGATTTGT
p-IL-6-qRT-F	CTGCTTCTGGTGATGGCTACTG
p-IL-6-qRT-R	GGCATCACCTTTGGCATCTT
p-TNF-α-qRT-F	ACCACGCTCTTCTGCCTACTGC
p-TNF-α-qRT-R	TCCCTCGGCTTTGACATTGGCTAC
p-IFN-β-qRT-F	GTTGCCTGGGACTCCTCAAT
p-IFN-β-qRT-R	ACGGTTTCATTCCAGCCAGT
p-IL-18-qRT-F	TCTACTCTCTCCTGTAAGAAC
p-IL-18-qRT-R	CTTATCATGTCCAGGAAC
p-IL-10-qRT-F	TTCAAACGAAGGACCAGATG
p-IL-10-qRT-R	TTCAAACGAAGGACCAGATG
p-TNF-α-qRT-F	CCAATGGCAGAGTGGGTATG
p-TNF-α-qRT-R	TGAAGAGGACCTGGGAGTAG
h-GAPDH-qRT-F	CGGGAAGCTTGTGATCAATGG
h-GAPDH-qRT-R	GGCAGTGATGGCATGGACTG
h-IL-1β-qRT-F	GCAAGGGCTTCAGGCAGGCCGCG
h-IL-1β-qRT-R	GGTCATTCTCCTGGAAGGTCTGTGGGC
h-IL-6-qRT-F	GGCCCTTGCTTTCTCTTCG
h-IL-6-qRT-R	GGCCCTTGCTTTCTCTTCG
h-TNF-α-qRT-F	ATGGGCTACAGGCTTGTCACTC
h-TNF-α-qRT-R	ATGGGCTACAGGCTTGTCACTC
h-IFN-β-qRT-F	AGTAGGCGACACTGTTCGTG
h-IFN-β-qRT-R	GCCTCCCATTCAATTGCCAC
m-GAPDH-qRT-F	TGATGGGTGTGAACCACGAG
m-GAPDH-qRT-R	GCCCTTCCACAATGCCAAAG
m-IFN-β-qPCR-F	GTCAACAACCCACAGGTCCA
m-IFN-β-qPCR-R	CGACTCCTTTTCCGCTTCCT
m-IL-1β-qRT-F	ATGGCAGAAGTACCTAAGCTC
m-IL-1β-qRT-R	TTAGGAAGACACAAATTGCATGGTGAACTCAGT
m-IL-6-qRT-F	TACCACTTCACAAGTCGGAGGC
m-IL-6-qRT-R	CTGCAAGTGCATCATCGTTGTTC
m-TNF-α-qRT-F	TCTTCCCTGAGCAATGC
m-TNF-α-qRT-R	GCTCCGTTTTCACAGAAAACATG
**siRNA or shRNA**	**Sequence (5’-3’)**
siDDX3X-1	UCUAAUCCUAUCUUUCCUCTT
siDDX3X-2	AUUUCUCCCAUCUCAACAUTT
siDDX3X-3	UAGUUGGUGCUAAUACCAATT
siEP364R-1	GCAGAAUAUUGAUCAGUUA
siEP364R-2	AGCUCUCCUAGAAAUCAAA
siEP364R-3	AGUAGCAGAUCAUCGAGAA
shEP364R-1-F	GATCCGCTTATAAGCATTAGCAAAGGCTCGAG CCTTTGCTAATGCTTATAAGCTTTTTGG
shEP364R-1-R	AATTCCAAAAAGCTTATAAGCATTAGCAAAGG CTCGAGCCTTTGCTAATGCTTATAAGCG
shEP364R-2-F	GATCCGCTTTCATCGCAGAATATTGACTCGAGTCAATATTCTGCGATGAAAGCTTTTTGG
shEP364R-2-R	AATTCCAAAAAGCTTTCATCGCAGAATATTGACTCGAGTCAATATTCTGCGATGAAAGCG
shEP364R-3-F	GATCCGCATTAGCAAAGGAAATAAGGCTCGAGCCTTATTTCCTTTGCTAATGCTTTTTGG
shEP364R-3-R	AATTCCAAAAAGCATTAGCAAAGGAAATAAGGCTCGAGCCTTATTTCCTTTGCTAATGCG
shASC-F	GTTTCACCGGAAGCTCGTCAACTACTACC
shASC-R	AAACGGTAGTAGTTGACGAGCTTCCGGTG
shNLRP3-F	GTTTCACCGGTGCAAGCTGGCTCGTTACC
shNLRP3-R	AAACGGTAACGAGCCAGCTTGCACCGGTG
shCaspase-1-F	GTTTCACCGGAACGCTACAGTTATGGATA
shCaspase-1-R	AAACTATCCATAACTGTAGCGTTCCGGTG

p represents porcine, h represents human, m represents mice.

### siRNA or shRNA interference

siRNAs or shRNA targeting DDX3X, EP364R, ASC, NLRP3, or Casp-1 were designed (sequences in Table 1), transfected into cells, and knockdown efficiency was validated. Briefly, for DDX3X-targeting siRNA, a total of 5 nmol siRNA was dissolved in DEPC water to prepare a 50 μM stock solution. Then, 1 μl of the siRNA was transfected into cells for 48 hours, followed by treatment with LPS+Nigericin or transfection with EP364R to activate NLRP3. For shRNA targeting EP364R, ASC, NLRP3, or Casp-1, cells were transfected for 48 hours and then infected with ASFV (MOI = 1). Samples were collected 24 hours post-infection for ELISA or Western blot analysis.

### Lentiviral preparation and Infection

HEK293T cells were seeded at 5 × 10⁶ cells/T75 flask and transfected with mix including 10 µg packaging plasmid psPAX2, 10 µg pMD2.G, and 10 µg target plasmid pHBLV-EP364R or pHBLV-Vector. The medium was replaced at 6 h post-transfection. Viral supernatant was collected at 48 h and 72 h post-transfection, filtered through a 0.45 µm filter, and centrifuged at 72,000 g for 120 min at 4°C. Then, HEK293T cells were seeded in a 96-well plate with 1 × 10⁴ cells/well. Lentivirus was serially 10-fold diluted, and added to the cells for overnight. After replacing the medium, GFP expression was observed, and the virus titer (TU/mL) was calculated. The viral pellet was resuspended in 500 µL fresh medium and stored at −80°C. For mice infection with lentivirus, 24 BALB/c mice (4-week-old) were randomly divided into Mock, PBS, LV-Vector, and LV-EP364R groups (n = 6). Mice were injected via the tail vein with 5 × 10⁷TU lentivirus twice at 48 h intervals. At 96 h post injection, mice were sacrificed. Serum was collected for ELISA detection, tissues were collected for RNA and protein extraction, and histopathological analysis.

### Enzyme-Linked Immunosorbent Assay (ELISA)

Blank control (Mock), treatment groups, and positive controls (LPS+Nigericin) were set up. Samples were freeze-thawed twice at −80°C, after vacuum freeze drying, resuspended in PBS, and centrifuged at 5000 r/min for 10 min. ELISA was employed to measure IL-1β, IL-6, IL-18, IL-10, IFN-γ, CRT, and TNF-α levels in the supernatant samples based on the instruction of the Ximbio ELISA kit instructions.

### Oligomerization Detection

Cells were crosslinked with DSS, pelleted, and analyzed by WB to detect oligomerized proteins. Briefly, after treatment, cells were collected by centrifugation at 2,000 rpm for 5 min at 4°C. The cell pellet was resuspended in 300 μL of PBS, 100 μL of this suspension was centrifuged and the pellet was lysed in cell lysis buffer for 4 hours. After lysis, the sample was centrifuged at 12,000 rpm for 10 min at 4°C. The supernatant was collected, mixed with protein loading buffer, and boiled at 100°C for 10 min to prepare the input sample. The remaining 200 μL of cell suspension was centrifuged, and the pellet was resuspended and treated with 2 mM disuccinimidyl suberate (DSS), followed by incubation in a metal bath at 37°C for 30 min. After crosslinking, protein loading buffer was added directly to the sample without further processing to prepare the pellet sample. Both input and pellet samples were subjected to WB analysis.

### Immunofluorescence Assay (IFA)

Adherent cells were cultured in confocal dishes until reaching 50% confluence, followed by transfection with plasmid or viral infection for 24 hours. Cells were then fixed with 4% paraformaldehyde at room temperature for 15 min. After removal of the fixative, permeabilization was performed using 0.1% Triton X-100 for 20 min at room temperature. The solution was discarded, and cells were blocked with 10% FBS at 37°C for 1 h. After blocking, the cells were incubated with primary antibody overnight at 4°C. The antibody was then removed, and the cells were washed four times with PBST, 5 min each. Subsequently, cells were incubated with a fluorescence-conjugated secondary antibody for 45 min at room temperature, protected from light. After removal of the secondary antibody, the cells were washed four times with PBST in the dark, 5 min per wash. Then, DAPI staining was performed for 10 min at room temperature under light protection. After discarding the DAPI solution, the cells were washed twice with PBST in the dark, 3 min each. Finally, PBST was added to cover the cells, and samples were imaged using a laser scanning confocal microscope.

### Transmission electron microscope (TEM) assay

Cells were seeded into 6-well plates and infected with ASFV at an MOI of 1, and the cells were harvested and fixed with 2% glutaraldehyde in PBS for 1 hour at 24 hpi. The samples were dehydrated, embedded, and stained according to standard procedures. The samples were observed and analyzed using a transmission electron microscope.

### Isolation and Culture of BMDMs from NLRP3^-/-^ Mice

Wild-type and NLRP3^-/-^ mice were sacrificed by cervical dislocation, and incubated in 75% ethanol for 5 min, Then, the hind limbs were isolated, and muscle was removed in sterile PBS. Cut the ends of the femur and tibia, and flush out the bone marrow with induction medium using a syringe, and repeatedly pipette to disperse cell clusters. Cells were filtered using a 70 µm cell strainer and centrifuged at 1500 r/min for 10 min, discard the supernatant and the cells in resuspend in BMDM induction medium. The collected cells were seed for 3 days (do not change medium during this process). The medium was half replaced at 3 days and entirely replaced at 5 days, respectively, post seed. The cells can be used for experiments on day 7.

## LDH, Casp-1 Activity, and ATP Assay

### LDH Release Assay

Cells were collected (minimum of 10⁴ cells) and lysed by adding extraction buffer (1 mL per 5 × 10⁶ cells), followed by ultrasonic disruption on ice (200 W power, 3 s sonication pulses with 10 s intervals, repeated 30 times). The lysate was centrifuged at 12,000 rpm and 4 °C for 10 min, and the supernatant was collected and kept on ice for subsequent use. The supernatant was mixed with the LDH detection working solution, prepared according to the instructions of the Solaibao (Beyotime)-LDH-Assay-Kit, and incubated at room temperature (approximately 25 °C) for 30 min in the dark (samples may be wrapped in aluminum foil and gently shaken on a horizontal or rocking shaker). Absorbance was measured at 490 nm.

### Caspase-1 activity assay

Cells were collected and washed once with PBS. Lysis buffer was added (100 µL per 2 × 10⁶ cells), and the pellet was resuspended and incubated on ice for 15 min. The lysate was centrifuged at 12,000 rpm and 4 °C for 20 min, and the supernatant was transferred to a pre-chilled tube. The reaction system and standards were prepared according to the instructions of the Beyotime-Caspase-1-Activity-Assay-Kit. The mixture was incubated at 37 °C for 60–120 min. Once a noticeable color change was observed, absorbance was measured at 405 nm. Further data analysis and statistics were performed.

### ATP level assay

Cells were collected, and 200 µL of lysis buffer per well (equivalent to 1/10 of the original 2 mL culture medium volume) was added to lyse the cells. The lysate was centrifuged at 12,000 rpm and 4 °C for 5 min, and the supernatant was collected for subsequent measurement. Reagents were thawed on ice before use. The detection reagent and standards were prepared according to the instructions of the Beyotime-ATP-Assay-Kit. Luminescence was measured using a luminometer, and relative light unit (RLU) values were recorded for further data analysis and statistics.

### CCK-8 cell viability assay

Cells were seeded in a 96-well plate at a density of 5,000 cells per well in 100 µL of medium and cultured under appropriate conditions. According to experimental requirements, plasmid transfection or viral infection was performed. After 24 h, 10 µL of CCK-8 solution was added to each well. A well containing culture medium without cells was included as a blank control, and LPS plus Nigericin (LPS + Nig)-treated cells were used as a positive control. The plate was incubated at 37°C in a 5% CO₂ atmosphere for 1 h. Absorbance was measured at 450 nm, and data analysis was performed according to the instructions provided by the Biosharp CCK-8 assay kit.

### Protein-protein docking and targeting EP364R inhibitors virtual screening

No crystal structures are currently available for the EP364R protein (369 aa) or porcine DDX3X (662 aa). In this study, we predicted their structures using AlphaFold 3.0. Structural assessment of EP364R revealed that the region spanning residues 305–366 exhibited low prediction confidence and was therefore excluded. The remaining structured regions of EP364R—comprising a nuclease domain (residues 1–160) and a DNA-binding domain (residues 161–304)—were used for molecular docking. The DDX3X model exhibited high overall confidence and was docked as a full-length structure.

Based on binding affinity estimates from molecular docking, 100 binding modes were generated for the EP364R/DDX3X complex. The calculated binding affinities between DDX3X and the nuclease domain of EP364R ranged from –71.4790 kcal/mol to –23.6872 kcal/mol, with a median value of –44.3487 kcal/mol. Affinities between DDX3X and the DNA-binding domain of EP364R ranged from –68.0725 kcal/mol to –19.8363 kcal/mol, with a median of –40.4307 kcal/mol.

Using these median values as thresholds, we selected 50 binding poses for further analysis: those with affinities lower than –44.3487 kcal/mol for the nuclease domain, lower than –40.4307 kcal/mol for the DNA-binding domain, and lower than –52.7687 kcal/mol for full-length EP364R. Protein–protein interaction interfaces within the complex were analyzed using MOE (2022). The results revealed the following specific residue interactions:

In pose 1, Gln30, Lys154, Lys50, and Met156 of EP364R form interactions with Glu388, Asp521, Glu523, Glu449, Pro519, and Arg528 of DDX3X.

In pose 2, Lys259, Arg226, and Lys252 of EP364R interact with Asp433, Asp558, and Asp426 of DDX3X.

In pose 3, Lys174, Met125, Val126, and Lys252 of EP364R form hydrogen bonds with Asp165, Val561, Lys418, Asp426, and Tyr200 of DDX3X.

Potential small molecule-binding sites on the EP364R protein were predicted using MOE, which identified two promising pockets—Site1 located in the nuclease domain and Site2 in the DNA-binding domain—suitable for small molecule binding. These sites were therefore selected for subsequent virtual screening. Site1 consists of the following residues: PHE3, VAL5, LYS39, GLN40, LEU41, PHE42, THR43, LEU47, CYS49, SER53, ILE55, LEU85. Site2 is composed of: GLU256, ILE257, LEU258, LYS259, ASP260, HIS261, ILE277, GLN278, ILE279, VAL280.

Molecular docking of each compound from the T001 library against the predicted structure of EP364R was performed using Schrödinger. Affinity scores were calculated for each compound. Among them, 6,001 compounds were docked into Site1, with binding affinities ranging from –8.9309 kcal/mol to –4.1953 kcal/mol. For Site2, 5,546 compounds were successfully docked, exhibiting affinity scores between –8.5520 kcal/mol and –3.8417 kcal/mol.

Due to variations in affinity distributions, compounds with binding affinities lower than –6 kcal/mol were selected, yielding a total of 310 compounds: 157 binding to Site1 and 153 to Site2. To assess structural diversity, the 157 compounds bound to Site1 and the 153 bounds to Site2 were subjected to structure-based clustering in MOE using the Jarvis-Patrick algorithm with a fingerprint similarity threshold of 70%. This grouped the Site1 set into 108 clusters and the Site2 set into 104 clusters.

The binding poses of the 108 clusters from Site1 and the 104 clusters from Site2 were visually inspected to eliminate unreasonable conformations. After curation, 73 compounds from Site1 and 61 from Site2 were retained for further analysis. These selected molecules were then applied to ASFV-treated cells for functional screening. Finally, the binding mode of HAMNO to EP364R was analyzed via 2D and 3D interaction visualization.

### Virus assays

PAMs or BMDMs were seed into 6-well plates at 6 × 10⁵ cells/well. After 4 h of culture at 37°C, add 50 µL of virus to each well. Continue culturing in a 37°C, 5% CO₂ incubator for 96 h, then collect all cells and medium, and store at −80°C until use. ASFVs were determined using qPCR assay. DNA extraction was performed using the method recommended by the manufacturer (DP315-F, TIANGEN BIOTECH Co.,LTD, Beijing, China). Absolute quantification RT-PCR primers for ASFV detection: 5’- GATACCACAAGATCAGCCGT-3’ (Forward), 5’-CTGCTCATGGTATCAATCTTATCGA-3’ (reverse), the TaqMan probes 5’FAM-CCACGGGAGGAATACCAACCCAGTG-TAMRA-3’. DNA amplification was performed for 39 cycles of 6 s at 95 °C and 11 s at 58 °C. The HAD assay was used to evaluate the infectivity of ASFV. Briefly, PAMs (5 × 10^4^) were seeded into 96-well plates, and infected with ASFV at an MOI of 0.01. At 48 hpi, porcine red blood cells (5 × 10^5^) were added to each well, and the “rosettes” of red blood cells was observed using an optical microscope.

### Biosafety statement

All experiments involving ASFV infection were performed in a biosafety level 3 (P3) facility at Lanzhou Veterinary Research Institute (LVRI) of the Chinese Academy of Agricultural Sciences (CAAS). This study was approved by the Ministry of Agriculture and Rural Affairs and were conducted in accordance with the recommendations in the *Guidelines for the Care and Use of Laboratory Animals* of the Ministry of Science and Technology of the PRC.

### Statistical analysis

Data were analyzed using GraphPad software (statistical significance using the student’s t test and ANOVA methods). Significance levels: * (*p* < 0.05), ** (*p* < 0.01), *** (*p* < 0.001), **** (*p* < 0.0001).

## Supporting information

S1 Fig ASFV infection induces Casp-1–dependent pyroptosis independent of Casp-3/4. (A) BMDMs were infected with ASFV (MOI = 1), after 24 h, cells were collected and subjected to WB for Casp-3/Casp-4 detection. (B-C) BMDMs were pretreated with the Casp‑3 inhibitor Z‑DEVD‑FMK (HY‑12466, 50 μM) or/ and the Casp-4 inhibitor Ac-LEVD-CHO (HY-136727, 20 μM) for 2 h, then infected with ASFV. Culture supernatants were collected 24 h post‑infection and analyzed by ELISA for IL‑1β secretion. And cells were collected and subjected for WB.
(TIF)

S2 Fig shRNA successfully knocks down ASC, NLRP3, and Casp-1 protein expression. (**A**) porcine iPAMs were transfected with 2 μg of shASC, shCasp-1, or shNLRP3 plasmids, respectively. Knockdown efficiency was validated by Western blot (WB) 72 hours post-transfection.
(TIF)

S3 Fig ASFV pEP364R induced pyroptosis and the release of damage-associated molecular patterns. (A) Porcine bone marrow-derived macrophages (BMDMs) were infected with ASFV at an MOI of 1. Cells were harvested at 0, 3, 6, 9, 12, 24, 48, 72, and 96 h post-infection (h p.i.). RNA was extracted, and the expression timing of EP364R during the ASFV replication cycle was determined by qPCR. (B) iPAMs were transfected with increasing concentrations (0.25, 0.5, 1.0 μg) of EP364R plasmid separately, or with 1 μg EV plasmid as a control. At 24 h p.t., Casp-1 activity was measured using a Casp-1 activity assay kit according to the manufacturer’s instructions. (C) iPAMs were transfected with 2 μg of EP364R plasmid. At 24 h p.t., cells were fixed with 2% glutaraldehyde, dehydrated, embedded, and stained following standard protocols. Cells were observed by transmission electron microscopy (TEM) for pyroptotic features. (D) iPAMs were transfected with 2 μg of EP364R plasmid. At 24 h p.t., cells were lysed and phosphorylation of IκBα and p65 was detected by WB. (E) iPAMs cells were transfected with 2 μg of EP364R plasmid. At 24 h p.t., RNA was extracted, and the transcriptional levels of cytokines (IL-1β, IL-6, TNF-α, IFN-β) were determined by one-step qPCR. (F) iPAMs were transfected with 3 μg of EP364R plasmid. At 24 h p.t., LDH, Calreticulin (CRT), and ATP release were measured using respective assay kits according to the manufacturers’ instructions. And p value less than 0.05 was considered statistically significant. *p < 0.05, **p < 0.01, ***p < 0.001.
(TIF)

S4 Fig EP364R overexpression lentivirus induced inflammatory responses in mice. (A) Six mice each from the LV-EP364R and LV-Vector groups were randomly selected and euthanized. Tissues (heart, liver, spleen, lung, kidney; 1 g each) were harvested, homogenized, and RNA was extracted. Transcriptional levels of cytokines IL-1β, IL-6, TNF-α, and IFN-β in these organs were determined by qPCR. (B) Six mice each from the LV-EP364R and LV-Vector groups were randomly selected and euthanized. Peripheral blood serum was isolated, and the secretion levels of cytokines IL-1β, IL-6, TNF-α, and IFN-β were measured by ELISA. And p value less than 0.05 was considered statistically significant. *p < 0.05, **p < 0.01, ***p < 0.001.
(TIF)

S5 Fig Impairing EP364R expression inhibits p65 and IκBα phosphorylation. (A) iPAMs cells were transfected with 2 μg of shEP364R-3 plasmid. At 72 h p.t., cells were infected with ASFV at MOI = 1 for 24 h. Phosphorylation of p65 and IκBα was detected by WB.
(TIF)

S6 Fig Deficiency in NLRP3 inflammasome components reduces EP364R-induced cell death. (A) iPAMs were pretreated with the NLRP3 inhibitor MCC950 (10 μM) or the Casp-1 inhibitor VX765 (10 μM), then transfected with 2 μg of EP364R plasmid. At 24 h p.t., cells were stained with PI (10 μl) and cell death was observed by fluorescence microscopy. (B) BMDMs from NLRP3-/- mice (prepared as in Fig 7G) were transfected with 3 μg of EP364R plasmid. At 24 h p.t., cells were stained with PI (10 μl) and cell death was observed by fluorescence microscopy.
(TIF)

S7 Fig DDX3X deficiency impairs ASFV-induced pyroptosis. (A) PAMs were co-transfected with siDDX3X-3 and shEP364R-3. At 72 h p.t., cells were infected with ASFV at MOI = 1 for 24 h. IL-1β secretion was measured by ELISA, and expression of IL-1β and GSDMD-N was detected by WB. (B) PAMs were co-transfected with siDDX3X-3 and shEP364R-3. At 72 h p.t., cells were infected with ASFV at MOI = 1 for 24 h. Cells were stained with PI (10 μl) and cell death was observed by fluorescence microscopy. And p value less than 0.05 was considered statistically significant. *p < 0.05, **p < 0.01, ***p < 0.001.
(TIF)

S8 Fig The DDX3X enzymatic activity inhibitor RK-33 does not affect EP364R-induced inflammasome assembly. (A-B) HEK293T (A) or iPAMs (B) cells were pretreated with RK-33 (5 μM) for 1 h, then transfected with 3 μg of EP364R plasmid. IL-1β secretion was measured by ELISA 24 h p.t. (C) HEK293T cells were pretreated with RK-33 (5 μM) for 1 h, then transfected with 3 μg of EP364R plasmid. Expression of IL-1β and GSDMD-N was detected by WB 24 h p.t. (D) HEK293T cells were pretreated with RK-33 (5 μM) for 1 h, then transfected with 3 μg of EP364R plasmid. At 24 h p.t., cell lysates were centrifuged; the supernatant was the ‘input’. The pellet was resuspended in PBS, cross-linked with DSS at 37°C for 30 min, and mixed with loading buffer to prepare the ‘pellet’ sample. ASC oligomerization was detected by WB. (E) HEK293T cells were pretreated with RK-33 (5 μM) for 1 h, then transfected with 3 μg of EP364R plasmid. ASC speck formation was observed by IFA 24 h p.t. (F) HEK-293T cells were pretreated with RK-33 (5 μM) for 1 h, then transfected with 3 μg of EP364R plasmid. At 24 h p.t., cells were stained with PI (10 μl) and cell death was observed by fluorescence microscopy. And p value less than 0.05 was considered statistically significant. *p < 0.05, **p < 0.01, ***p < 0.001.
(TIF)

S9 Fig EP364R interacted with DDX3X via spatial structure. (A) Design strategy for plasmids encoding different domains of DDX3X and NLRP3. (B) Structural model of EP364R and DDX3X (used alphafold3). (C) Three potential spatial interaction modes between EP364R and DDX3X.
(TIF)

S10 Fig The natural product HAMNO inhibits ASFV replication by targeting EP364R. (A) Natural products targeting EP364R were analyzed using MOE software. (B) PAMs were treated with different compounds (30 μM) and then infected with ASFV-GFP at MOI = 1. GFP fluorescence was observed by fluorescence microscopy 24 h p.i. to screen for effective compounds from a screened library. (**C**) PAMs were treated with the identified inhibitory compounds (30 μM) and then infected with ASFV-GFP at MOI = 1. ASFV copies were assessed by RT-qPCR 24 h p.i. (**D**) PAMs were treated with the identified inhibitory compounds (30 μM) and then infected with ASFV-GFP at MOI = 1. Cell viability was assessed using the CCK-8 assay (10 μl reagent, 37°C, 1 h, measure 450 nm) 24 h p.i. (**E**) Predicted binding site of HAMNO on the EP364R structure. HAMNO binds to site 2. (**F and G**) 3D (**F**) and 2D (**G**) views of the binding conformation between HAMNO and EP364R. (**H**) PAMs were treated with increasing concentrations of HAMNO (0, 10, 20, 30 μM) and then infected with ASFV-GFP at MOI = 1. ASFV replication was assessed by HAD50 and p72 protein expression was detected by WB 24 h p.i. (**I**) PAMs were treated with increasing concentrations of HAMNO (0, 10, 20, 30 μM) and then infected with ASFV-GFP at MOI = 1. GFP fluorescence was observed by fluorescence microscopy 24 h p.i. (**J**) PAMs were treated with HAMNO at different time points relative to infection (-4 (before), -2, 0, 2, 4, 8 h p.i./p.t.), then infected with ASFV-GFP at MOI = 1. ASFV replication was assessed by HAD50 and p72 protein expression was detected by WB 24 h p.i. (**K**) PAMs were treated with HAMNO at different time points relative to infection (-4(before), -2, 0, 2, 4, 8 h p.i./p.t.), then infected with ASFV-GFP at MOI = 1. GFP fluorescence was observed by fluorescence microscopy 24 h p.i. (**L-O**) BMDMs were pretreated with HAMNO (30 μM) or an equivalent volume of DMSO for 4 hours. Cells were then infected with ASFV at a multiplicity of infection (MOI = 1). Samples were collected 24 hours post-infection (h.p.i.), or iPAMs were transfected with 5 μg of an EP364R-expressing plasmid. After media change, cells were treated with HAMNO (30 μM) or an equivalent volume of DMSO, samples were collected 24 hours post-transfection, or HEK293T cells were transfected with 5 μg of an EP364R-expressing plasmid and subsequently treated with HAMNO (30 μM) or DMSO. Cells were lysed 24 hours post-transfection, 100 μl of the total cell lysate was reserved as the Input control. Culture supernatants were subjected to repeated freeze-thaw cycles and lyophilization, followed by ELISA to quantify secreted IL-1β. Cell lysates were prepared for Western blot analysis to detect the protein levels of the inflammatory cytokine IL-1β and the pyroptosis executioner N-GSDMD. Total RNA was extracted for qPCR to measure the transcriptional levels of the cytokines IL-1β, IL-18, and TNF-α. Co-immunoprecipitation results indicated that the interaction among EP364R, NLRP3, and DDX3X was abolished upon HAMNO treatment. And p value less than 0.05 was considered statistically significant. *p < 0.05, **p < 0.01, ***p < 0.001.
(TIF)

S1 File All the Figure’s WB original data. (PDF)

S1 Data The original data and the data used for plotting in Fig 1. (ZIP)

S2 Data The original data and the data used for plotting in Fig 2. (ZIP)

S3 Data The original data and the data used for plotting in Fig 3A-3DFigure 3BFigure 3CFigure 3D. (ZIP)

S4 Data The original data and the data used for plotting in Fig 3E and 3F. (ZIP)

S5 Data The original data and the data used for plotting in Fig 4. (ZIP)

S6 Data The original data and the data used for plotting in Fig 5. (ZIP)

S7 Data The original data and the data used for plotting in Fig 6. (ZIP)

S8 Data The original data and the data used for plotting in Fig 7A-7DFigure 7BFigure 7CFigure 7D. (ZIP)

S9 Data The original data and the data used for plotting in Fig 7E-7HFigure 7FFigure 7GFigure 7H. (ZIP)

S10 Data The original data and the data used for plotting in Fig 8. (ZIP)

S11 Data The original data and the data used for plotting in Fig 9. (ZIP)

S12 Data The original data and the data used for plotting in Fig 10. (ZIP)

S13 Data The original data and the data used for plotting in Fig 11. (ZIP)

S14 Data The original data and the data used for plotting in S1 Fig. (ZIP)

S15 Data The original data and the data used for plotting in S2 Fig. (ZIP)

S16 Data The original data and the data used for plotting in S3 Fig. (ZIP)

S17 Data The original data and the data used for plotting in S4 Fig. (ZIP)

S18 Data The original data and the data used for plotting in S5 Fig. (ZIP)

S19 Data The original data and the data used for plotting in S7 Fig. (ZIP)

S20 Data The original data and the data used for plotting in S8 Fig. (ZIP)

S21 Data The original data and the data used for plotting in S10 Fig. (ZIP)

S1 Text Describe. (DOCX)
