# Supplementary figures and images for: African swine fever virus pEP364R acts as an important inflammatory-inducing factor to activate NLRP3 inflammasome-mediated pyroptosis by regulating DDX3X
Source: PLoS Pathog. 2026 Feb 25;22(2):e1013874. doi: 10.1371/journal.ppat.1013874 (PMC12952717; doi:10.1371/journal.ppat.1013874)

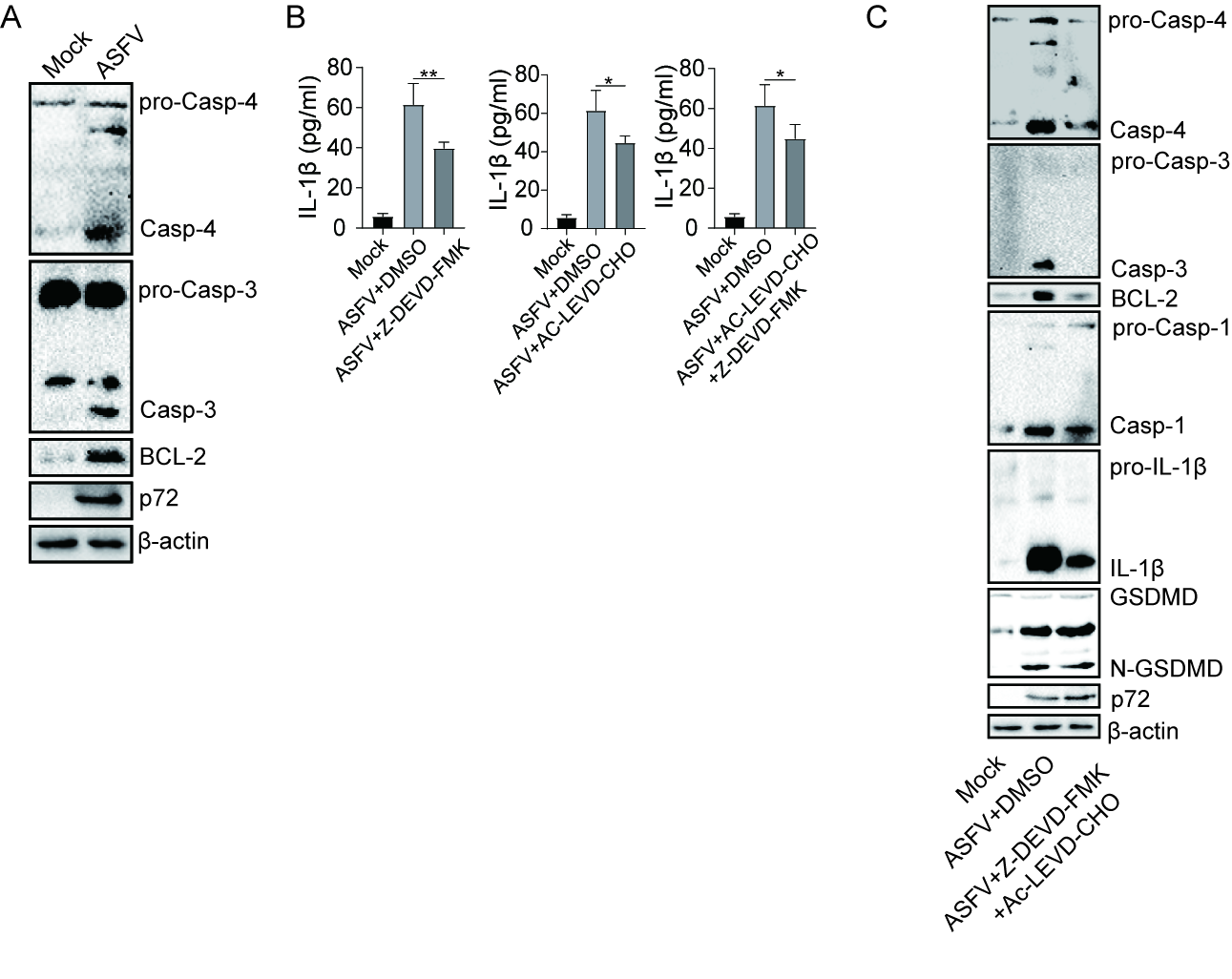

Supplement: S1 Fig — (A) BMDMs were infected with ASFV (MOI = 1), after 24 h, cells were collected and subjected to WB for Casp-3/Casp-4 detection. (B-C) BMDMs were pretreated with the Casp‑3 inhibitor Z‑DEVD‑FMK (HY‑12466, 50 μM) or/ and the Casp-4 inhibitor Ac-LEVD-CHO (HY-136727, 20 μM) for 2 h, then infected with ASFV. Culture supernatants were collected 24 h post‑infection and analyzed by ELISA for IL‑1β secretion. And cells were collected and subjected for WB. (TIF) [file ppat.1013874.s001.tif]

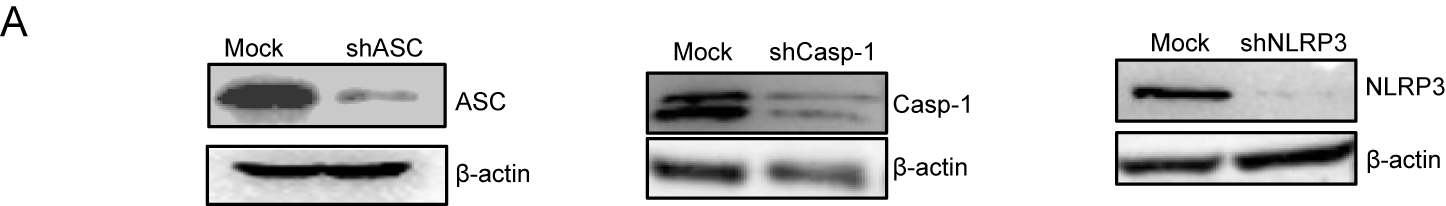

Supplement: S2 Fig — (A) porcine iPAMs were transfected with 2 μg of shASC, shCasp-1, or shNLRP3 plasmids, respectively. Knockdown efficiency was validated by Western blot (WB) 72 hours post-transfection. (TIF) [file ppat.1013874.s002.tif]

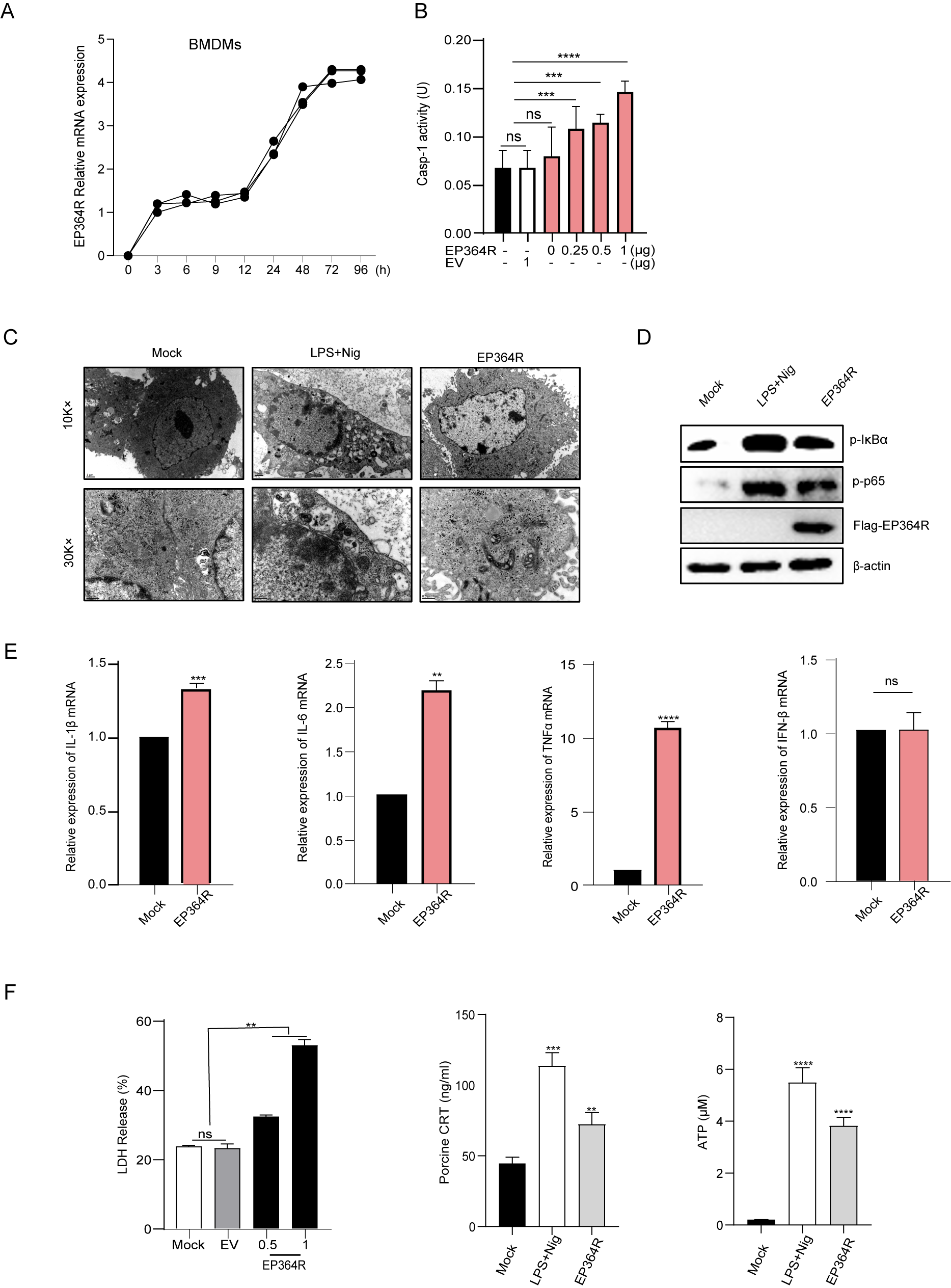

Supplement: S3 Fig — (A) Porcine bone marrow-derived macrophages (BMDMs) were infected with ASFV at an MOI of 1. Cells were harvested at 0, 3, 6, 9, 12, 24, 48, 72, and 96 h post-infection (h p.i.). RNA was extracted, and the expression timing of EP364R during the ASFV replication cycle was determined by qPCR. (B) iPAMs were transfected with increasing concentrations (0.25, 0.5, 1.0 μg) of EP364R plasmid separately, or with 1 μg EV plasmid as a control. At 24 h p.t., Casp-1 activity was measured using a Casp-1 activity assay kit according to the manufacturer’s instructions. (C) iPAMs were transfected with 2 μg of EP364R plasmid. At 24 h p.t., cells were fixed with 2% glutaraldehyde, dehydrated, embedded, and stained following standard protocols. Cells were observed by transmission electron microscopy (TEM) for pyroptotic features. (D) iPAMs were transfected with 2 μg of EP364R plasmid. At 24 h p.t., cells were lysed and phosphorylation of IκBα and p65 was detected by WB. (E) iPAMs cells were transfected with 2 μg of EP364R plasmid. At 24 h p.t., RNA was extracted, and the transcriptional levels of cytokines (IL-1β, IL-6, TNF-α, IFN-β) were determined by one-step qPCR. (F) iPAMs were transfected with 3 μg of EP364R plasmid. At 24 h p.t., LDH, Calreticulin (CRT), and ATP release were measured using respective assay kits according to the manufacturers’ instructions. And p value less than 0.05 was considered statistically significant. *p < 0.05, **p < 0.01, ***p < 0.001. (TIF) [file ppat.1013874.s003.tif]

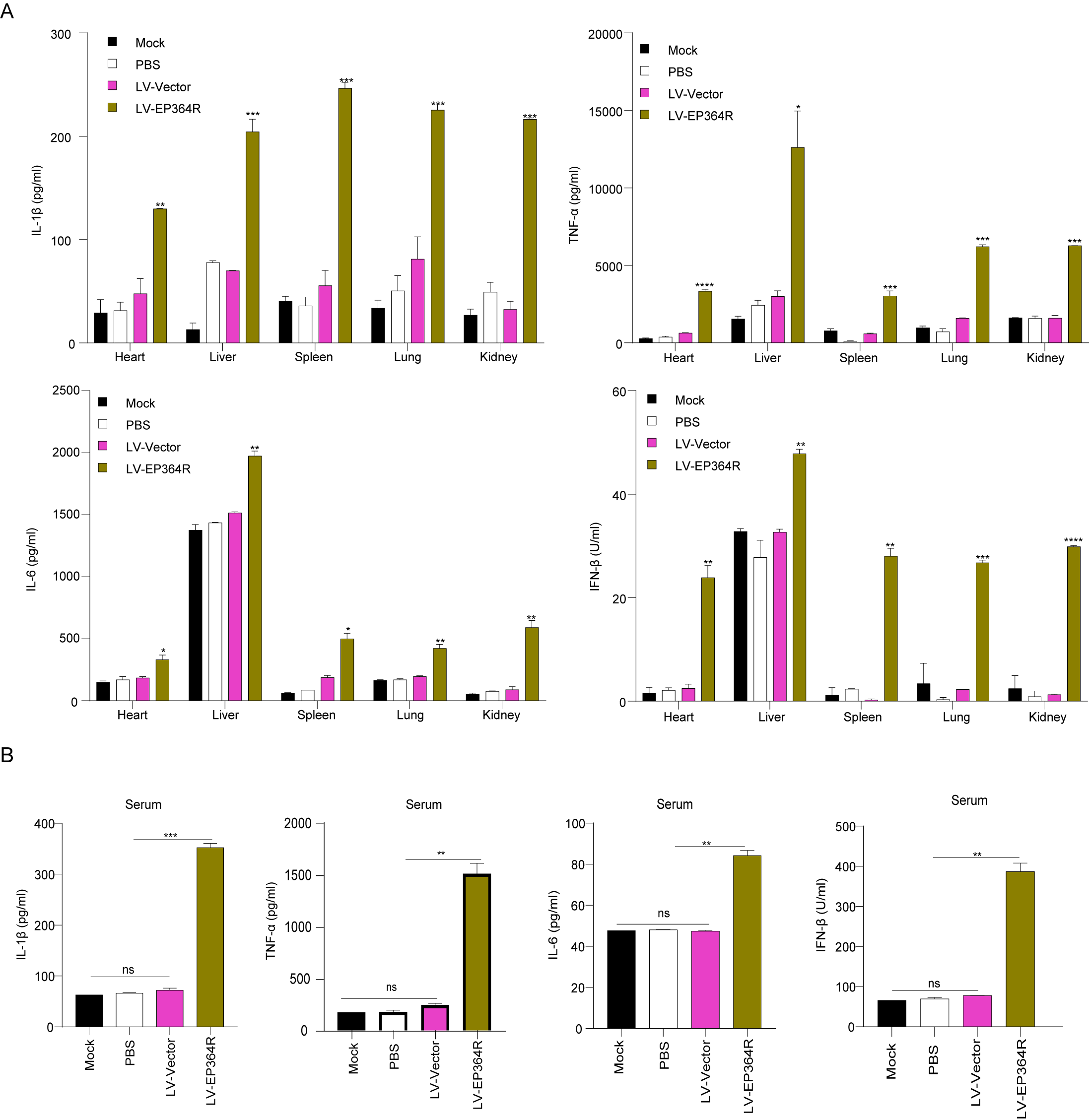

Supplement: S4 Fig — (A) Six mice each from the LV-EP364R and LV-Vector groups were randomly selected and euthanized. Tissues (heart, liver, spleen, lung, kidney; 1 g each) were harvested, homogenized, and RNA was extracted. Transcriptional levels of cytokines IL-1β, IL-6, TNF-α, and IFN-β in these organs were determined by qPCR. (B) Six mice each from the LV-EP364R and LV-Vector groups were randomly selected and euthanized. Peripheral blood serum was isolated, and the secretion levels of cytokines IL-1β, IL-6, TNF-α, and IFN-β were measured by ELISA. And p value less than 0.05 was considered statistically significant. *p < 0.05, **p < 0.01, ***p < 0.001. (TIF) [file ppat.1013874.s004.tif]

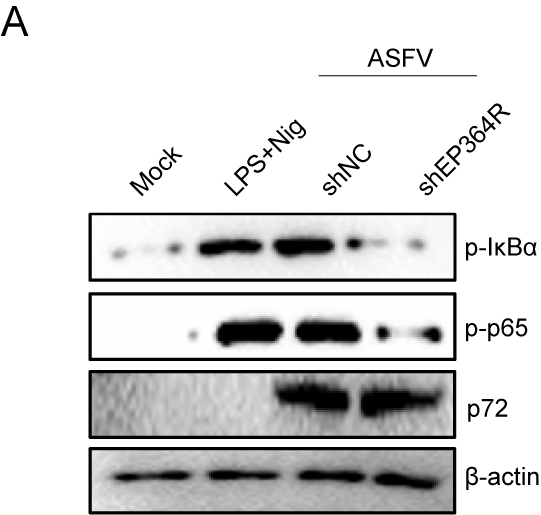

Supplement: S5 Fig — (A) iPAMs cells were transfected with 2 μg of shEP364R-3 plasmid. At 72 h p.t., cells were infected with ASFV at MOI = 1 for 24 h. Phosphorylation of p65 and IκBα was detected by WB. (TIF) [file ppat.1013874.s005.tif]

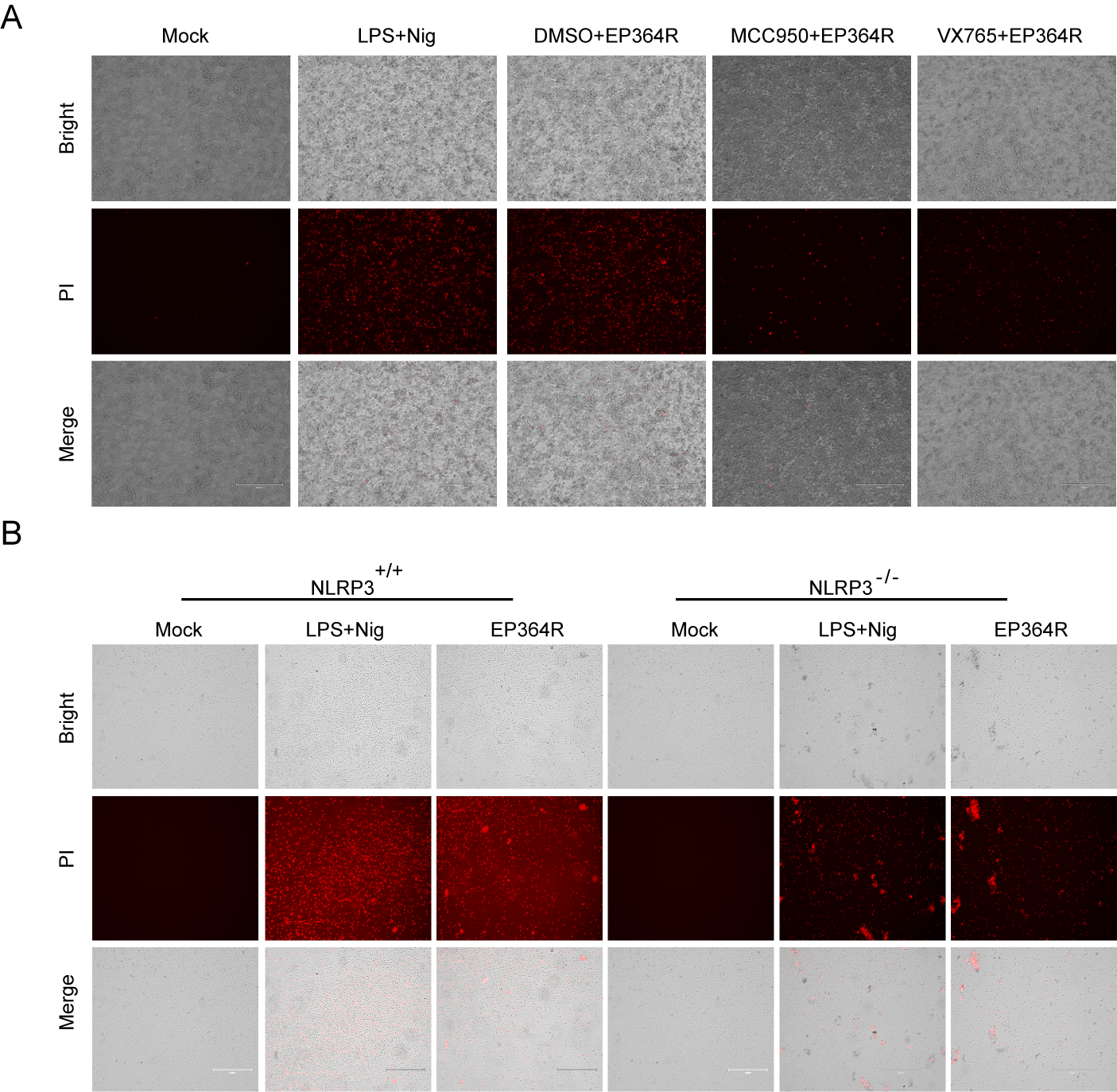

Supplement: S6 Fig — (A) iPAMs were pretreated with the NLRP3 inhibitor MCC950 (10 μM) or the Casp-1 inhibitor VX765 (10 μM), then transfected with 2 μg of EP364R plasmid. At 24 h p.t., cells were stained with PI (10 μl) and cell death was observed by fluorescence microscopy. (B) BMDMs from NLRP3-/- mice (prepared as in Fig 7G) were transfected with 3 μg of EP364R plasmid. At 24 h p.t., cells were stained with PI (10 μl) and cell death was observed by fluorescence microscopy. (TIF) [file ppat.1013874.s006.tif]

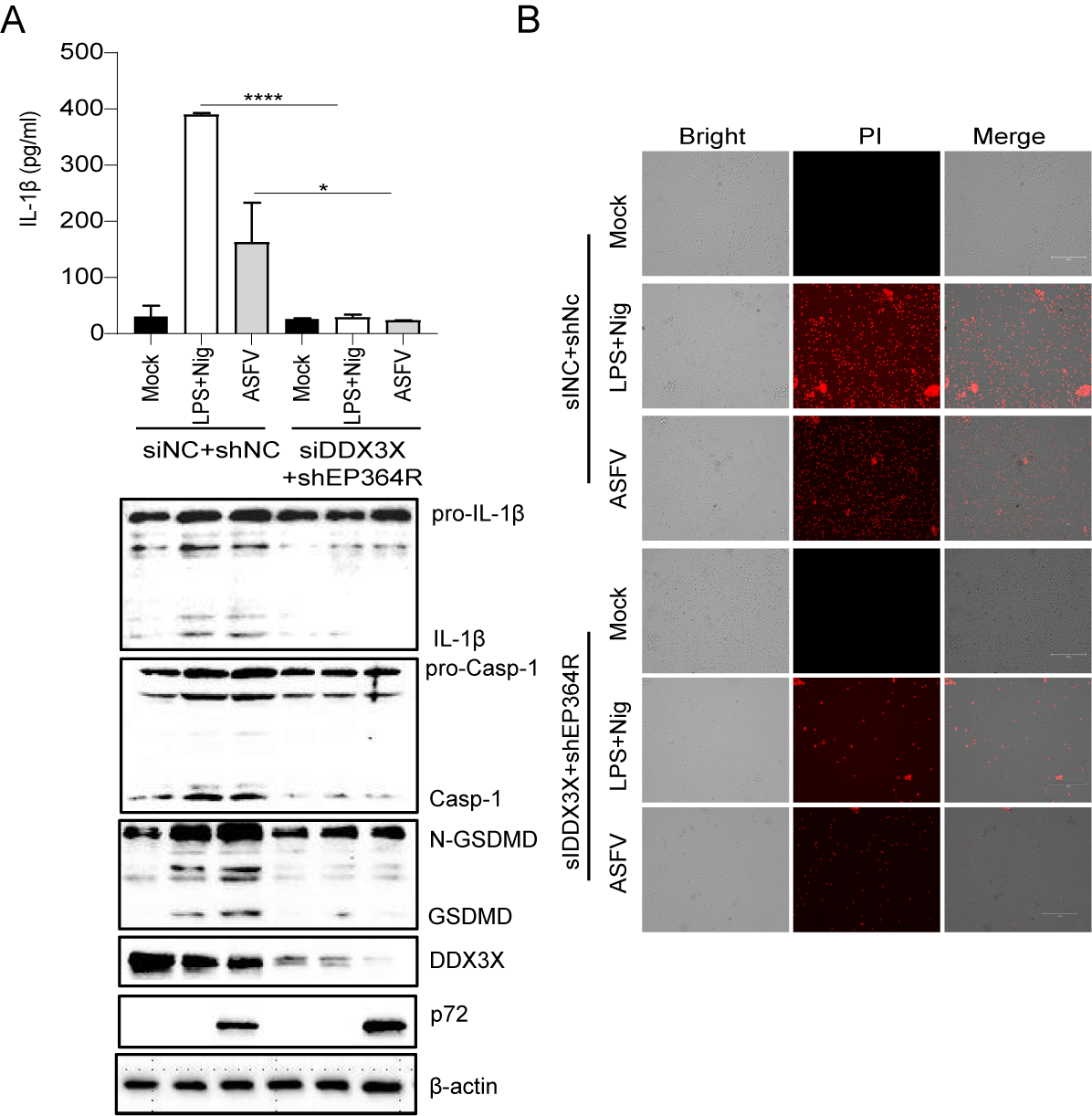

Supplement: S7 Fig — (A) PAMs were co-transfected with siDDX3X-3 and shEP364R-3. At 72 h p.t., cells were infected with ASFV at MOI = 1 for 24 h. IL-1β secretion was measured by ELISA, and expression of IL-1β and GSDMD-N was detected by WB. (B) PAMs were co-transfected with siDDX3X-3 and shEP364R-3. At 72 h p.t., cells were infected with ASFV at MOI = 1 for 24 h. Cells were stained with PI (10 μl) and cell death was observed by fluorescence microscopy. And p value less than 0.05 was considered statistically significant. *p < 0.05, **p < 0.01, ***p < 0.001. (TIF) [file ppat.1013874.s007.tif]

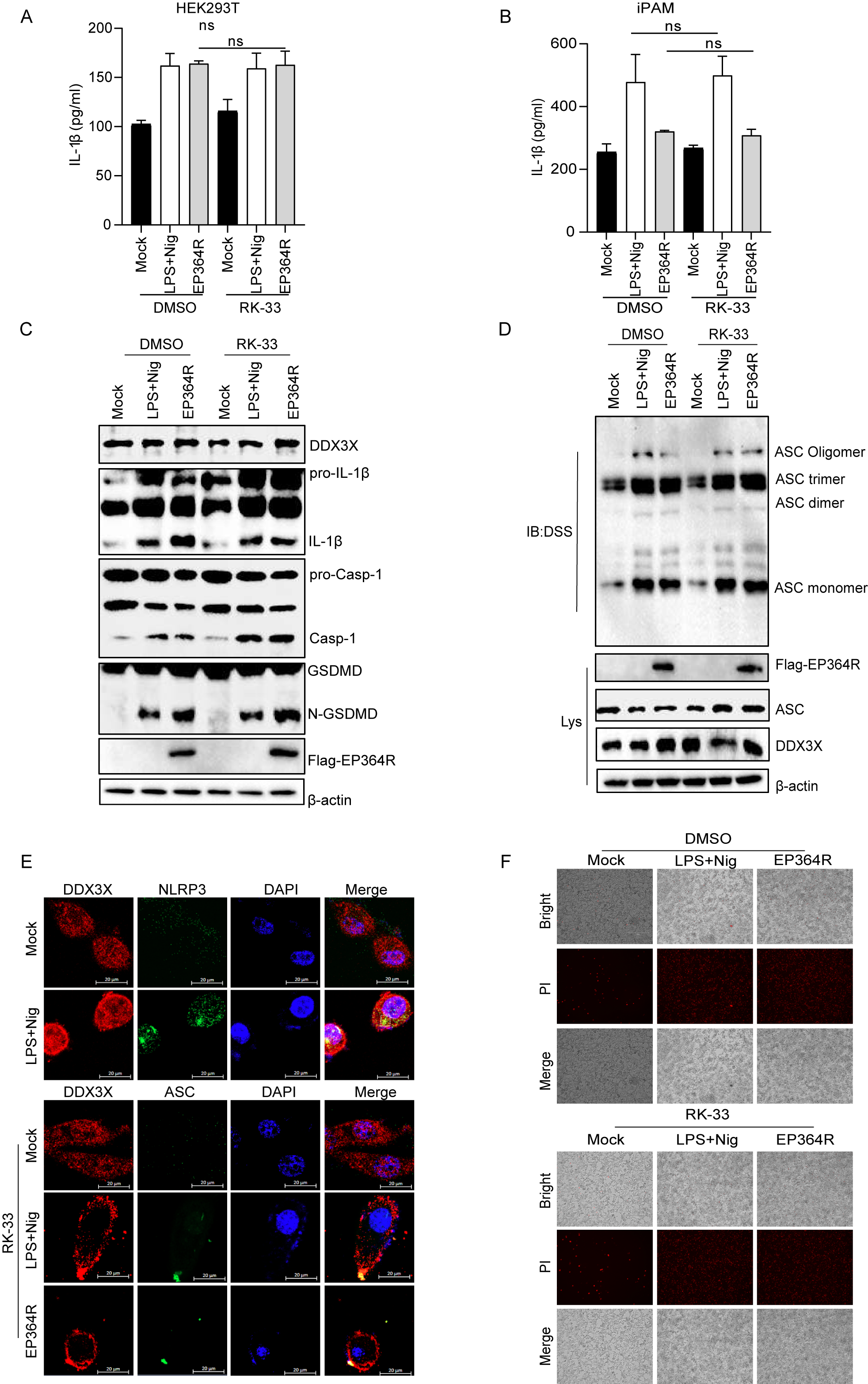

Supplement: S8 Fig — (A-B) HEK293T (A) or iPAMs (B) cells were pretreated with RK-33 (5 μM) for 1 h, then transfected with 3 μg of EP364R plasmid. IL-1β secretion was measured by ELISA 24 h p.t. (C) HEK293T cells were pretreated with RK-33 (5 μM) for 1 h, then transfected with 3 μg of EP364R plasmid. Expression of IL-1β and GSDMD-N was detected by WB 24 h p.t. (D) HEK293T cells were pretreated with RK-33 (5 μM) for 1 h, then transfected with 3 μg of EP364R plasmid. At 24 h p.t., cell lysates were centrifuged; the supernatant was the ‘input’. The pellet was resuspended in PBS, cross-linked with DSS at 37°C for 30 min, and mixed with loading buffer to prepare the ‘pellet’ sample. ASC oligomerization was detected by WB. (E) HEK293T cells were pretreated with RK-33 (5 μM) for 1 h, then transfected with 3 μg of EP364R plasmid. ASC speck formation was observed by IFA 24 h p.t. (F) HEK-293T cells were pretreated with RK-33 (5 μM) for 1 h, then transfected with 3 μg of EP364R plasmid. At 24 h p.t., cells were stained with PI (10 μl) and cell death was observed by fluorescence microscopy. And p value less than 0.05 was considered statistically significant. *p < 0.05, **p < 0.01, ***p < 0.001. (TIF) [file ppat.1013874.s008.tif]

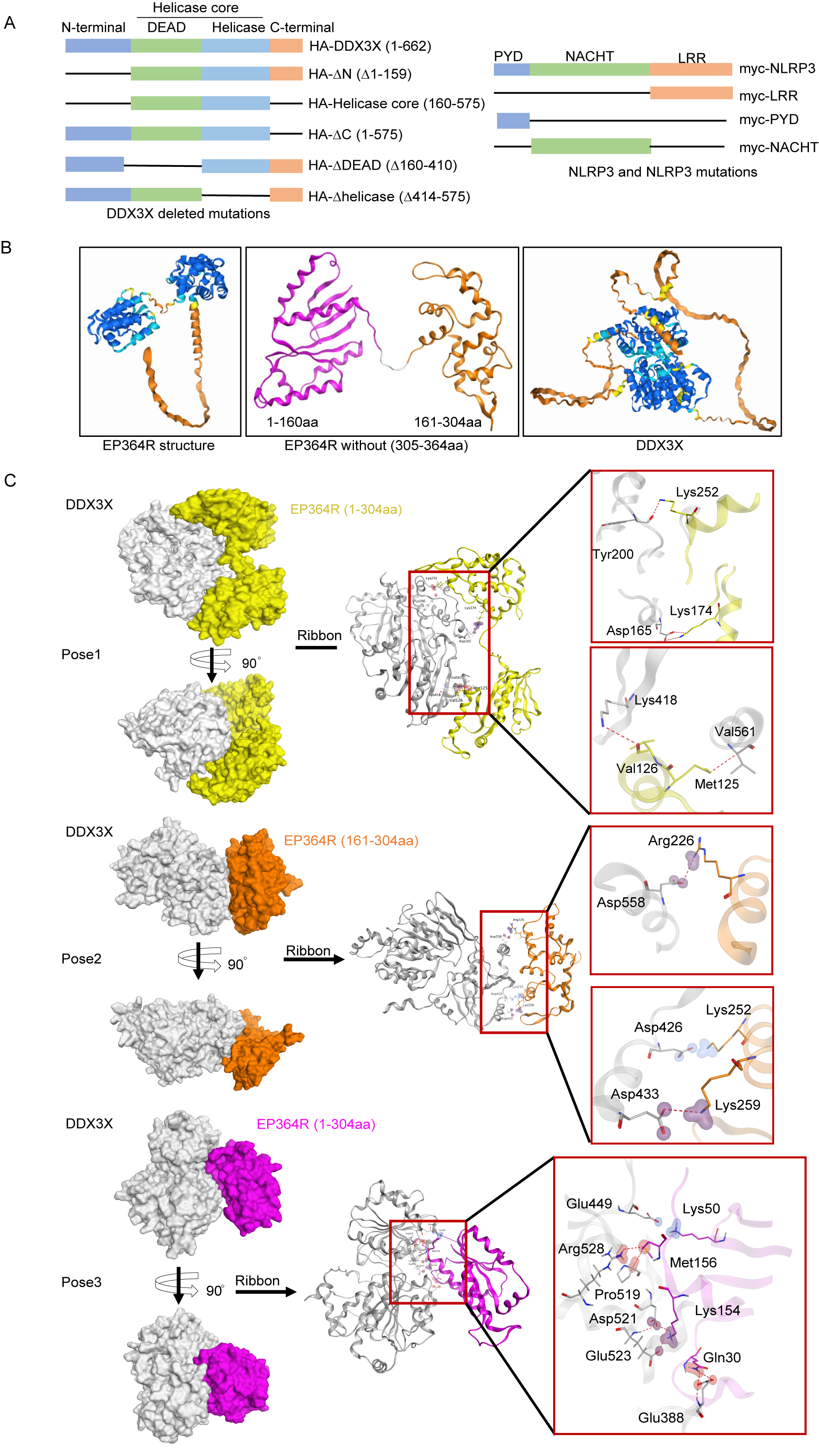

Supplement: S9 Fig — (A) Design strategy for plasmids encoding different domains of DDX3X and NLRP3. (B) Structural model of EP364R and DDX3X (used alphafold3). (C) Three potential spatial interaction modes between EP364R and DDX3X. (TIF) [file ppat.1013874.s009.tif]

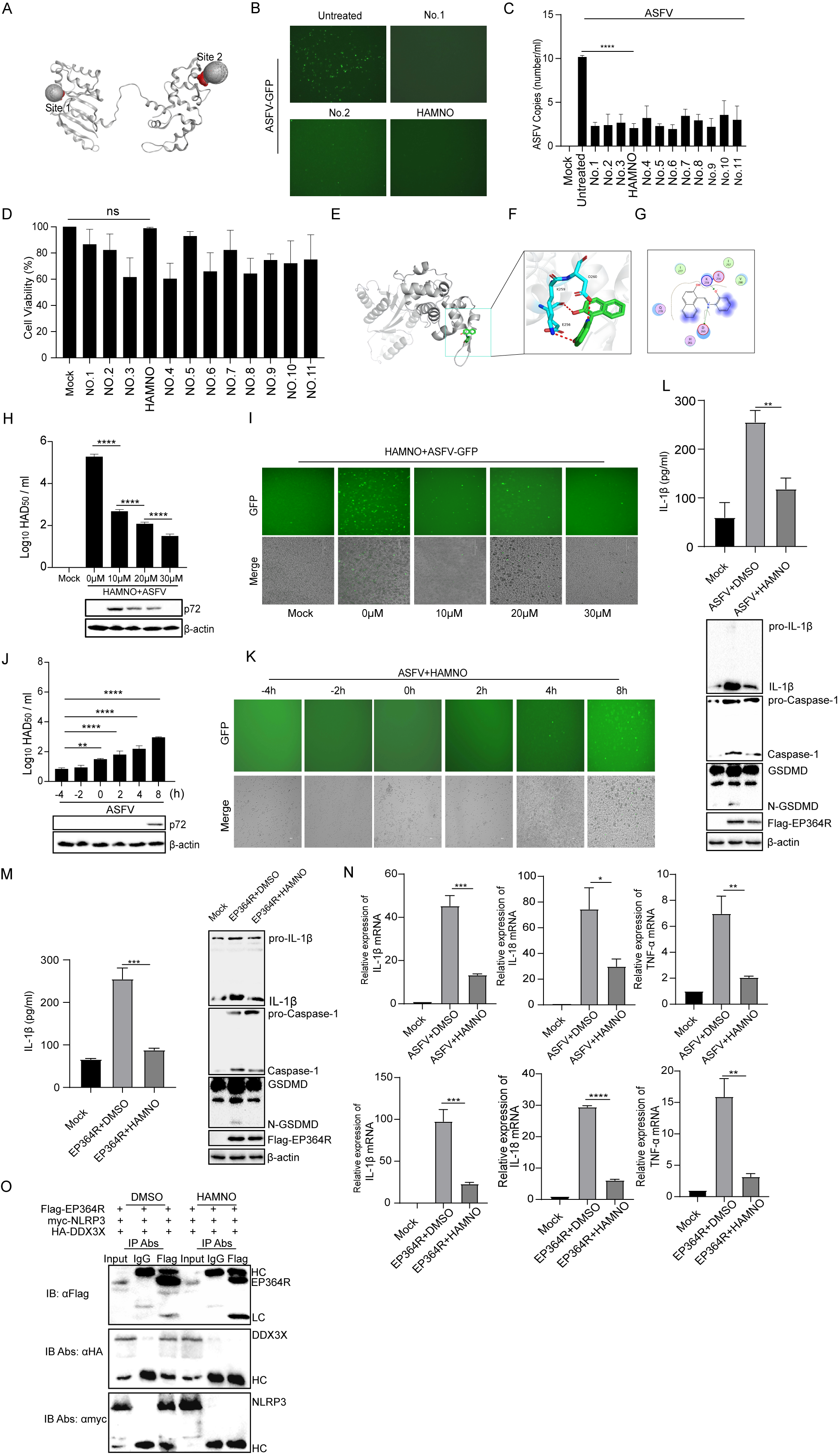

Supplement: S10 Fig — (A) Natural products targeting EP364R were analyzed using MOE software. (B) PAMs were treated with different compounds (30 μM) and then infected with ASFV-GFP at MOI = 1. GFP fluorescence was observed by fluorescence microscopy 24 h p.i. to screen for effective compounds from a screened library. (C) PAMs were treated with the identified inhibitory compounds (30 μM) and then infected with ASFV-GFP at MOI = 1. ASFV copies were assessed by RT-qPCR 24 h p.i. (D) PAMs were treated with the identified inhibitory compounds (30 μM) and then infected with ASFV-GFP at MOI = 1. Cell viability was assessed using the CCK-8 assay (10 μl reagent, 37°C, 1 h, measure 450 nm) 24 h p.i. (E) Predicted binding site of HAMNO on the EP364R structure. HAMNO binds to site 2. (F and G) 3D (F) and 2D (G) views of the binding conformation between HAMNO and EP364R. (H) PAMs were treated with increasing concentrations of HAMNO (0, 10, 20, 30 μM) and then infected with ASFV-GFP at MOI = 1. ASFV replication was assessed by HAD50 and p72 protein expression was detected by WB 24 h p.i. (I) PAMs were treated with increasing concentrations of HAMNO (0, 10, 20, 30 μM) and then infected with ASFV-GFP at MOI = 1. GFP fluorescence was observed by fluorescence microscopy 24 h p.i. (J) PAMs were treated with HAMNO at different time points relative to infection (-4 (before), -2, 0, 2, 4, 8 h p.i./p.t.), then infected with ASFV-GFP at MOI = 1. ASFV replication was assessed by HAD50 and p72 protein expression was detected by WB 24 h p.i. (K) PAMs were treated with HAMNO at different time points relative to infection (-4(before), -2, 0, 2, 4, 8 h p.i./p.t.), then infected with ASFV-GFP at MOI = 1. GFP fluorescence was observed by fluorescence microscopy 24 h p.i. (L-O) BMDMs were pretreated with HAMNO (30 μM) or an equivalent volume of DMSO for 4 hours. Cells were then infected with ASFV at a multiplicity of infection (MOI = 1). Samples were collected 24 hours post-infection (h.p.i.), or iPAMs were transfected [file ppat.1013874.s010.tif]

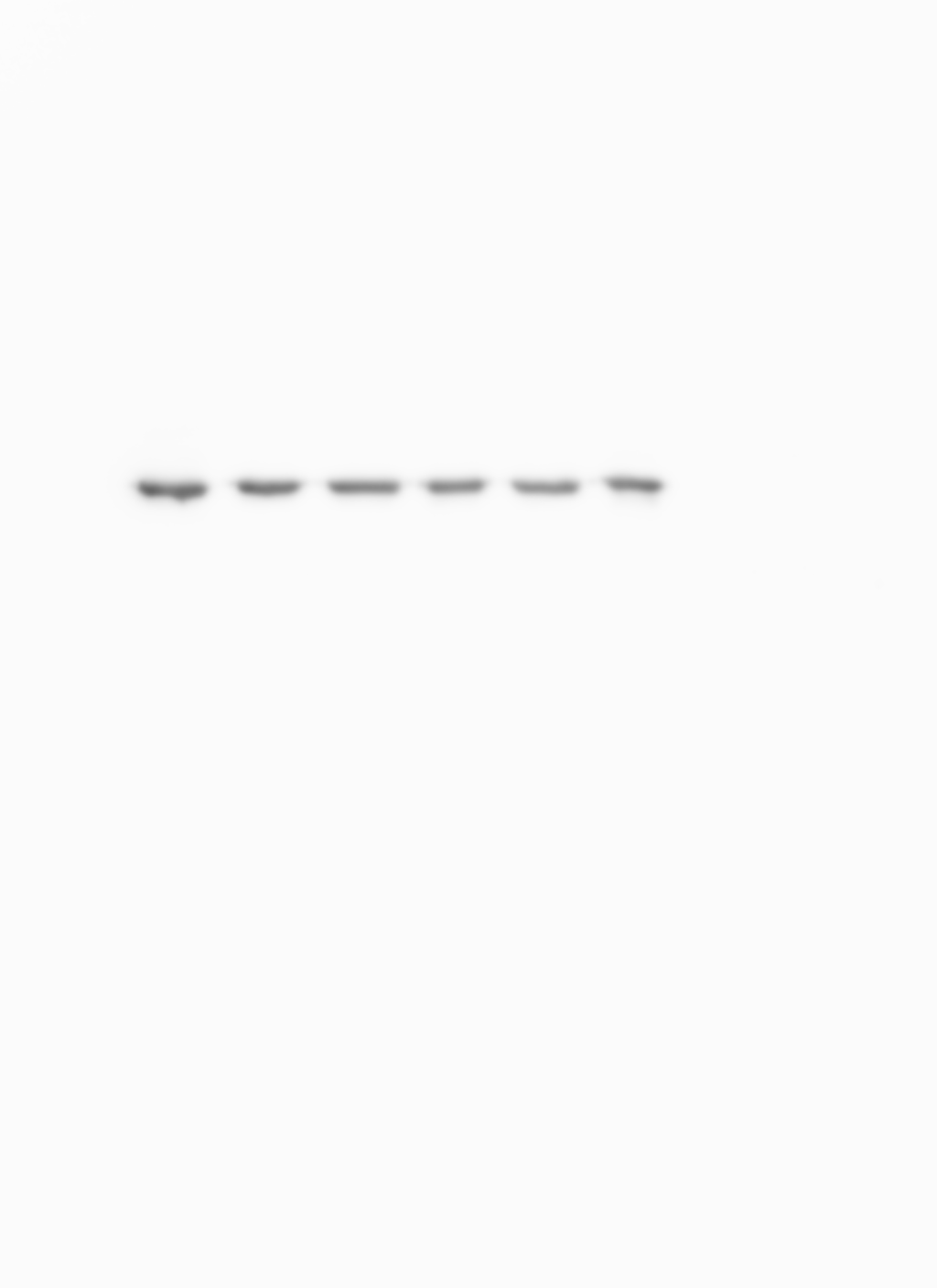

Supplement: S1 Data — (ZIP) [file ppat.1013874.s012.zip › S1_Data/Fig1/D/ASFV Infect-6,12,24h-β-Actin/p-actin 2022.06.09_.tif]

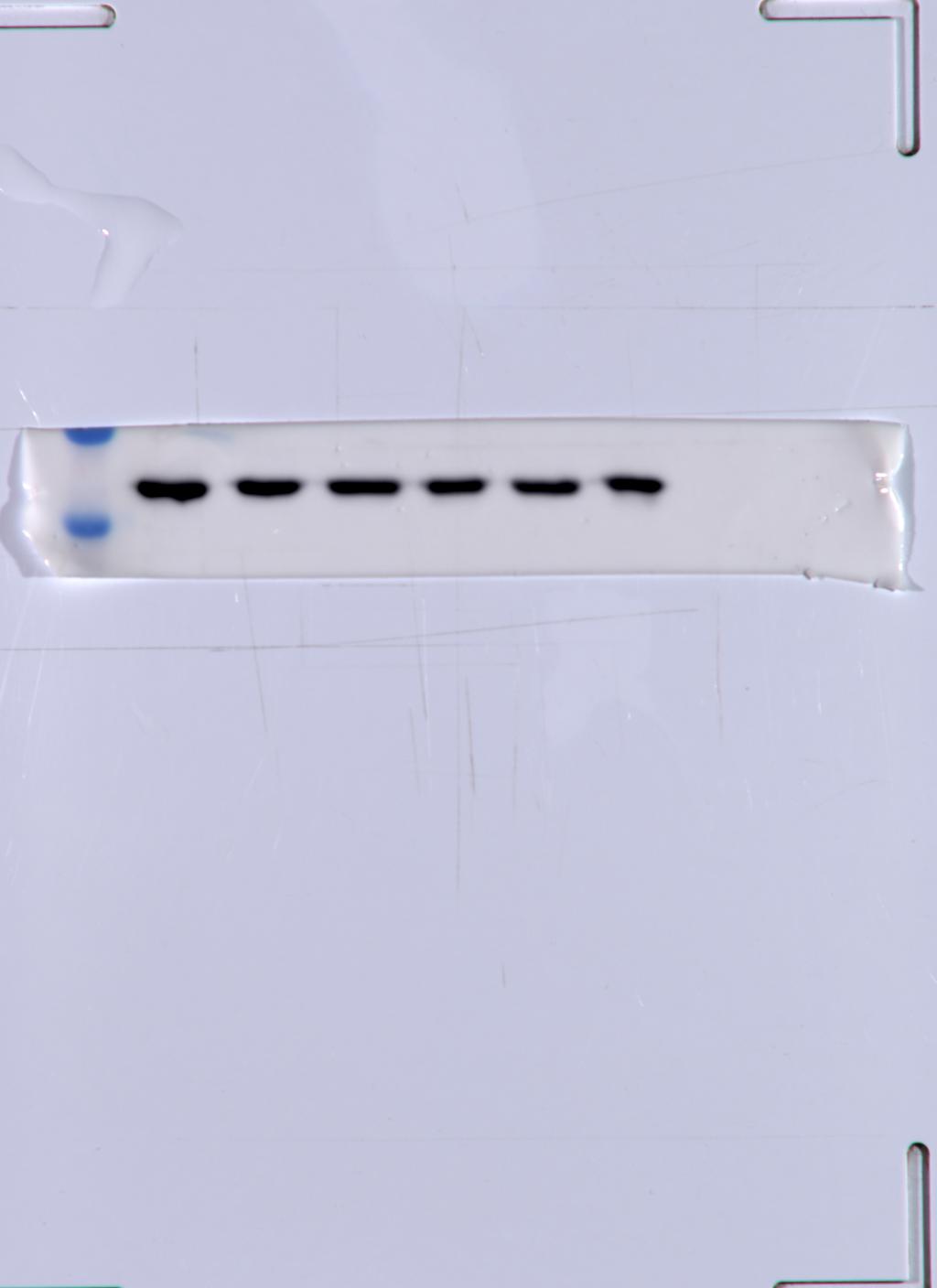

Supplement: S1 Data — (ZIP) [file ppat.1013874.s012.zip › S1_Data/Fig1/D/ASFV Infect-6,12,24h-β-Actin/p-actin 2022.06.09_Ch+Marker.jpg]

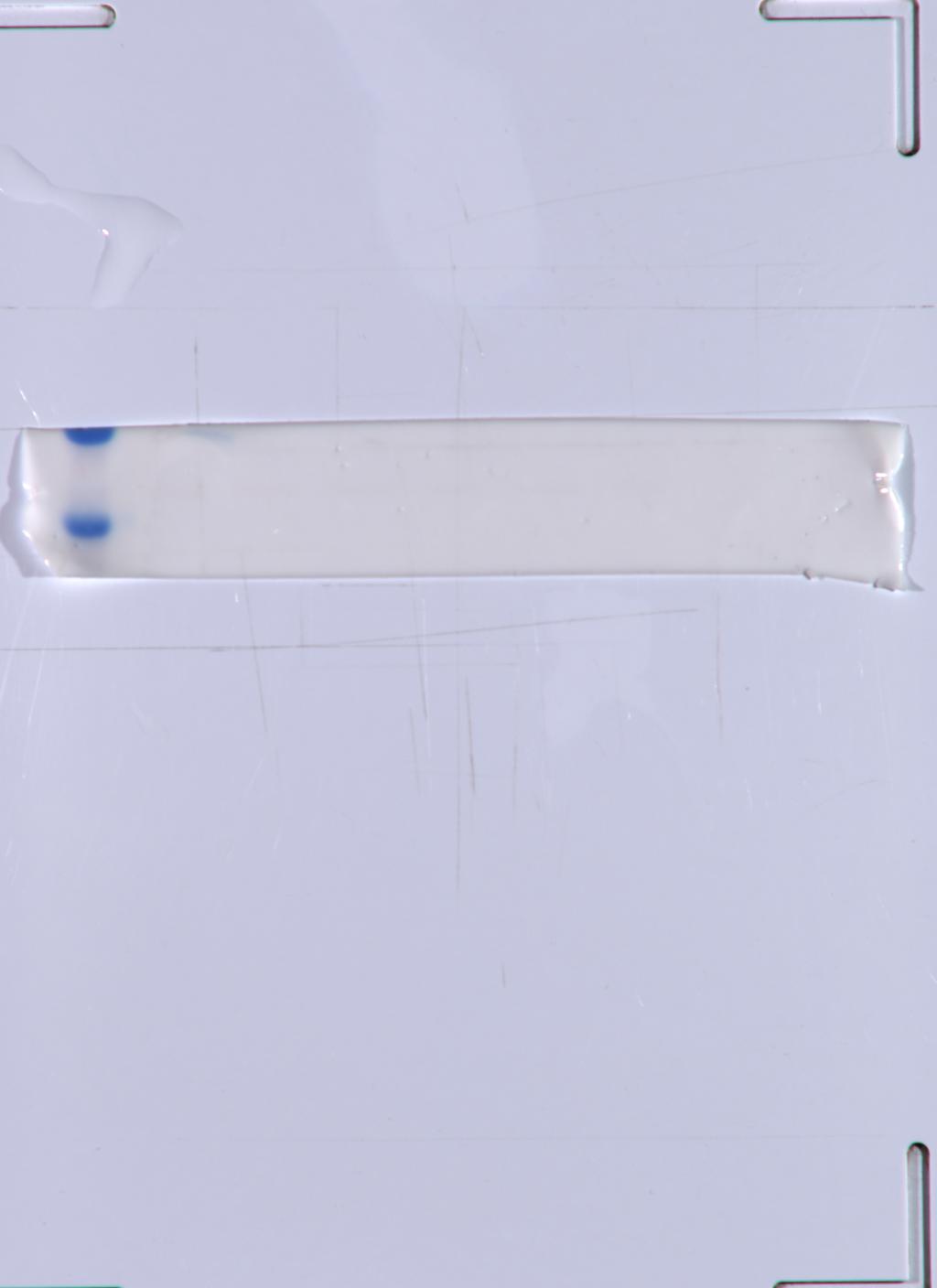

Supplement: S1 Data — (ZIP) [file ppat.1013874.s012.zip › S1_Data/Fig1/D/ASFV Infect-6,12,24h-β-Actin/p-actin 2022.06.09_Ch-Marker.jpg]

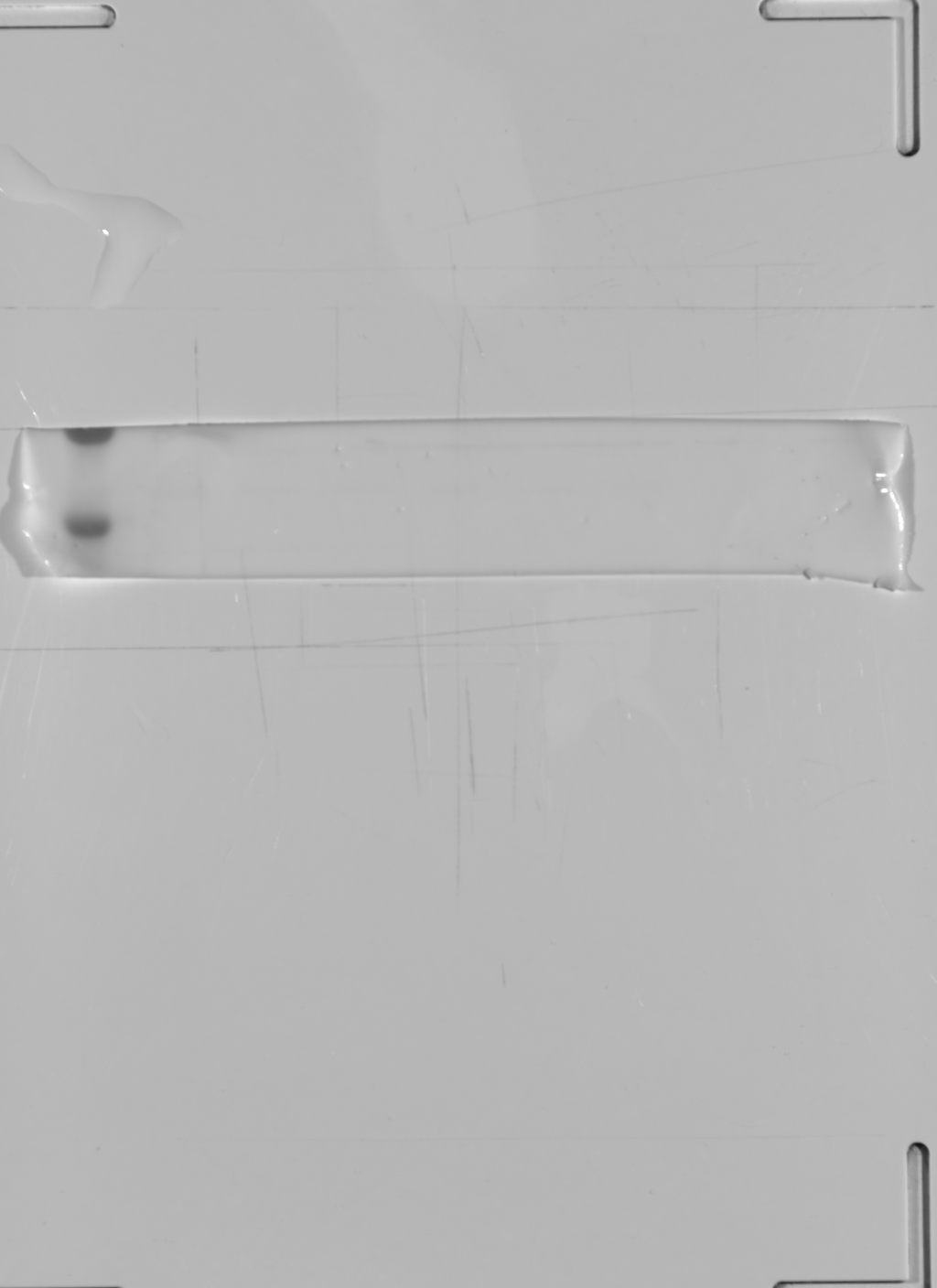

Supplement: S1 Data — (ZIP) [file ppat.1013874.s012.zip › S1_Data/Fig1/D/ASFV Infect-6,12,24h-β-Actin/p-actin 2022.06.09_Ch-Marker.tif]

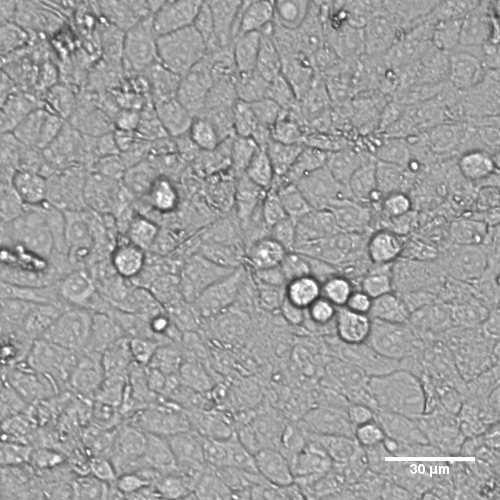

Supplement: S5 Data — (ZIP) [file ppat.1013874.s016.zip › S5_Data/Fig4/G/EP364R.tif]

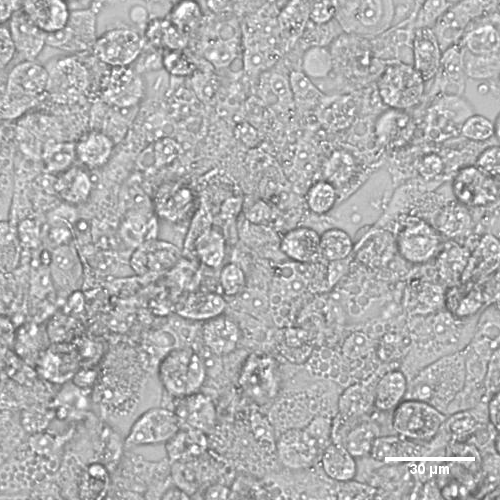

Supplement: S5 Data — (ZIP) [file ppat.1013874.s016.zip › S5_Data/Fig4/G/LPS+Nig.tif]

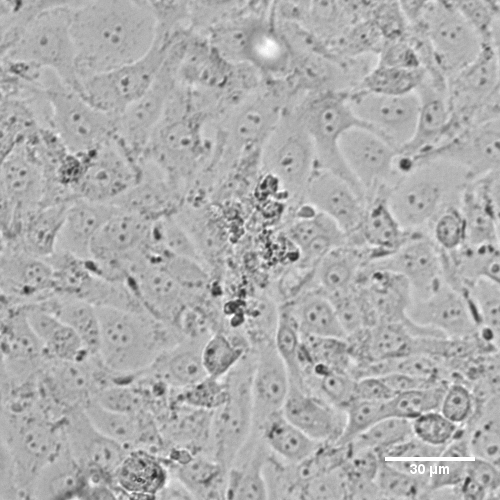

Supplement: S5 Data — (ZIP) [file ppat.1013874.s016.zip › S5_Data/Fig4/G/Mock.tif]

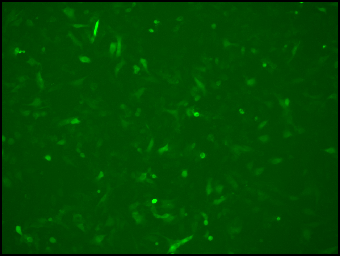

Supplement: S6 Data — (ZIP) [file ppat.1013874.s017.zip › S6_Data/Fig5/A/Fluorescence image/EP364R.png]

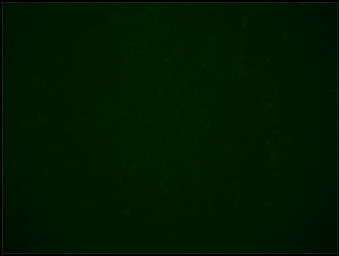

Supplement: S6 Data — (ZIP) [file ppat.1013874.s017.zip › S6_Data/Fig5/A/Fluorescence image/Mock.png]

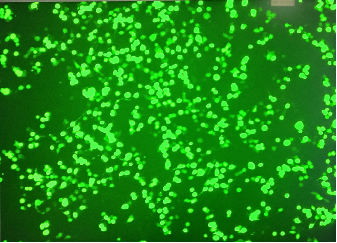

Supplement: S6 Data — (ZIP) [file ppat.1013874.s017.zip › S6_Data/Fig5/E/Fluorescence image/EP364R-GFP.png]

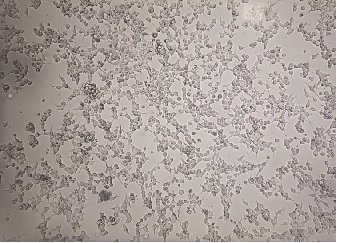

Supplement: S6 Data — (ZIP) [file ppat.1013874.s017.zip › S6_Data/Fig5/E/Fluorescence image/EP364R-TRANS.png]

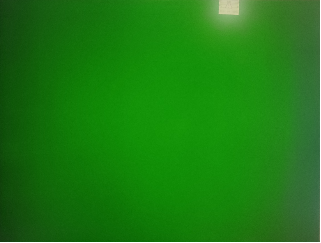

Supplement: S6 Data — (ZIP) [file ppat.1013874.s017.zip › S6_Data/Fig5/E/Fluorescence image/Mock-GFP.png]

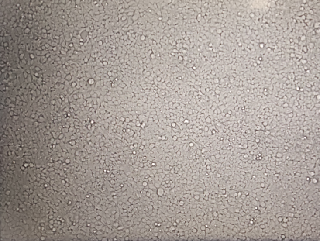

Supplement: S6 Data — (ZIP) [file ppat.1013874.s017.zip › S6_Data/Fig5/E/Fluorescence image/MOCK-TRANS.png]

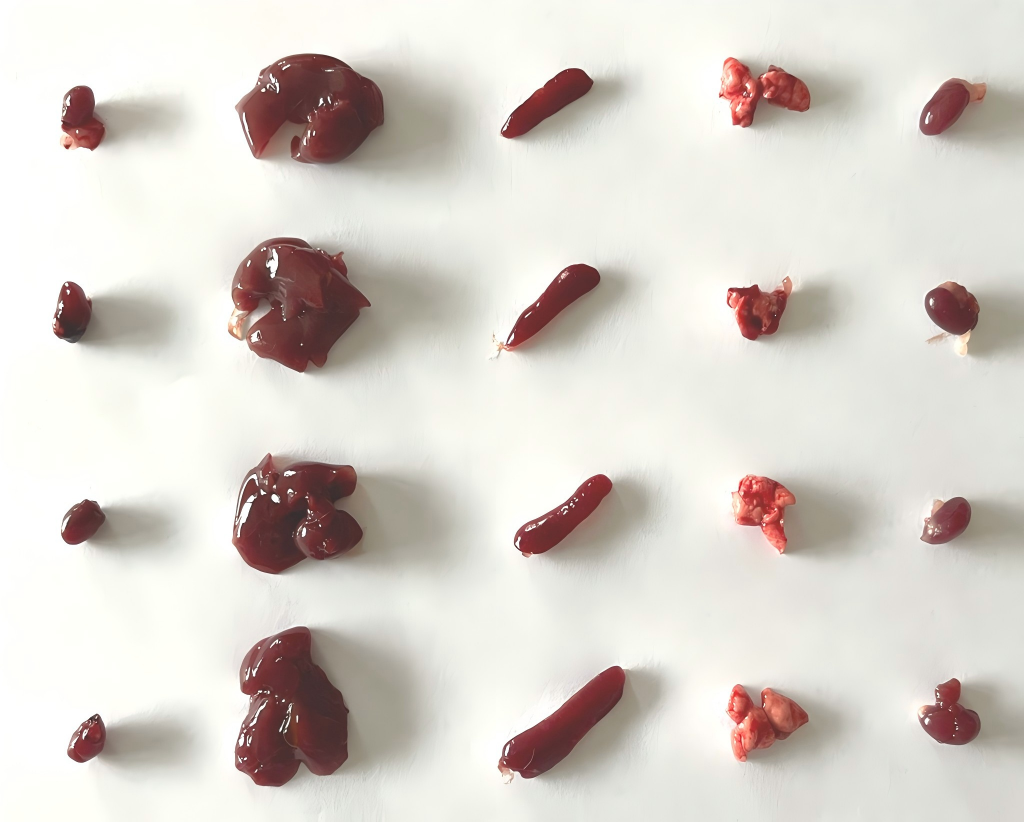

Supplement: S6 Data — (ZIP) [file ppat.1013874.s017.zip › S6_Data/Fig5/F/LV-EP364R感染小鼠组织形态.png]
