# Supplementary material for: African swine fever virus pEP364R acts as an important inflammatory-inducing factor to activate NLRP3 inflammasome-mediated pyroptosis by regulating DDX3X
Source: PLoS Pathog. 2026 Feb 25;22(2):e1013874. doi: 10.1371/journal.ppat.1013874 (PMC12952717; doi:10.1371/journal.ppat.1013874)
Supplement: S1 File — (PDF) [file ppat.1013874.s011.pdf]

Fig1D

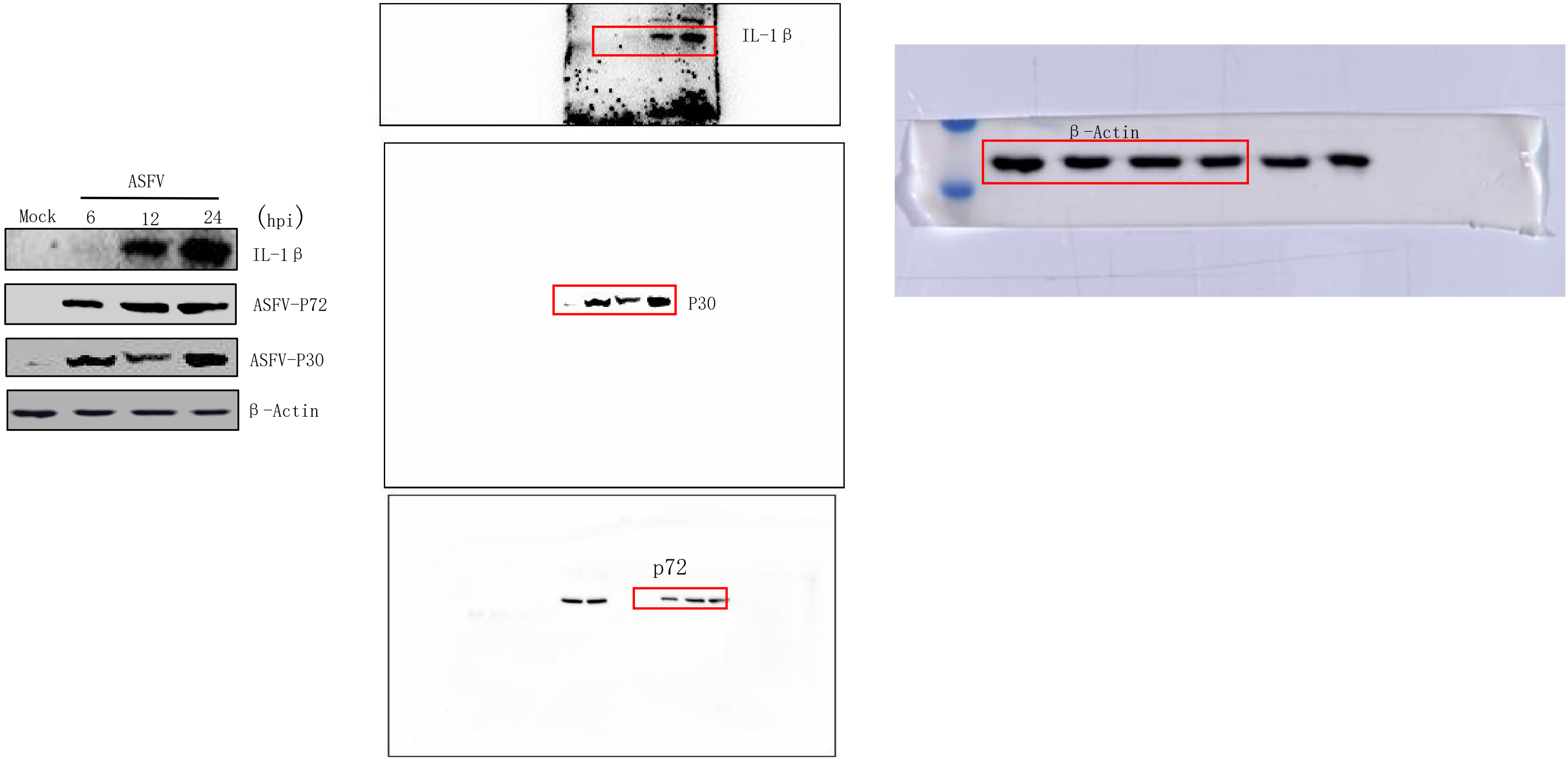
ASFV
Mock
6
12
24
(hpi)
IL-1β
ASFV-P72
ASFV-P30
β-Actin
IL-1β
P30
p72
β-Actin

Fig2E

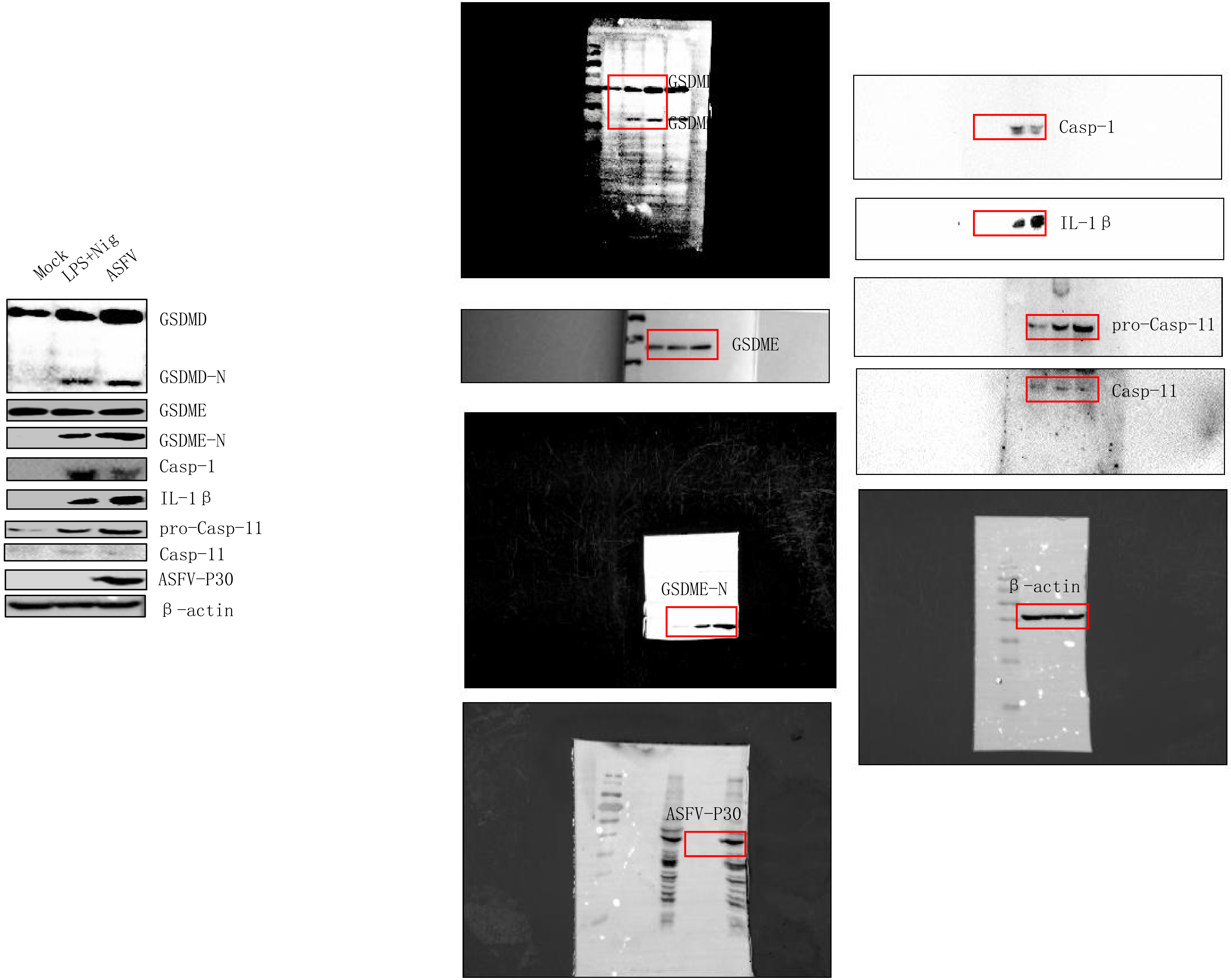
Mock
LPS+Nig
ASFV
GSDMD
GSDMD-N
GSDME
GSDME-N
Casp-1
IL-1β
pro-Casp-11
Casp-11
ASFV-P30
β-actin
GSDME
GSDME-N
ASFV-P30
Casp-1
IL-1β
pro-Casp-11
Casp-11
β-actin

Fig2H

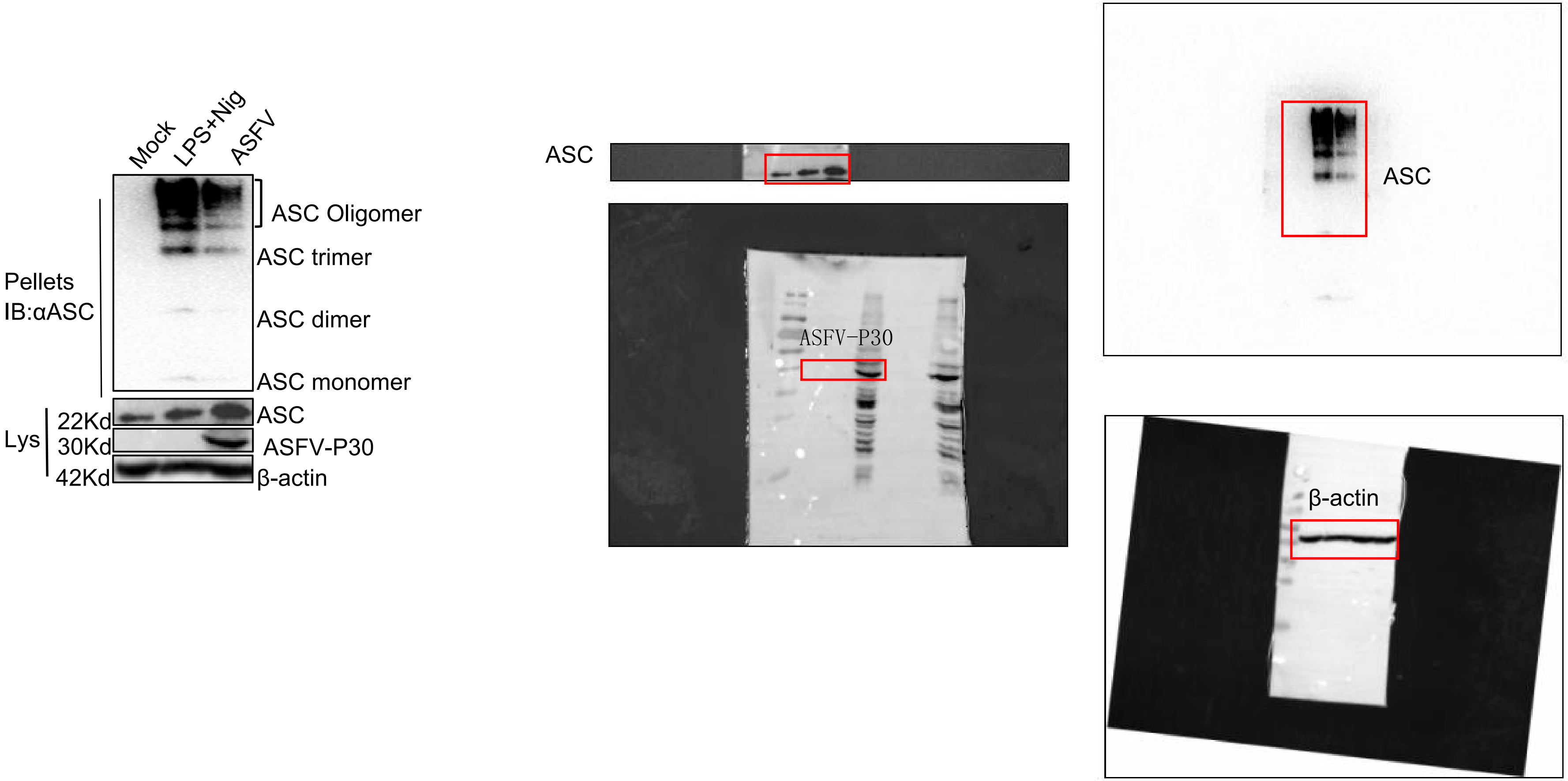
Mock
LPS+Nig
ASFV
ASC Oligomer
ASC trimer
Pellets
IB:αASC
ASC dimer
ASC monomer
Lys
22Kd
30Kd
42Kd
ASC
ASFV-P30
β-actin
ASC
ASFV-P30
ASC
β-actin

Fig2I

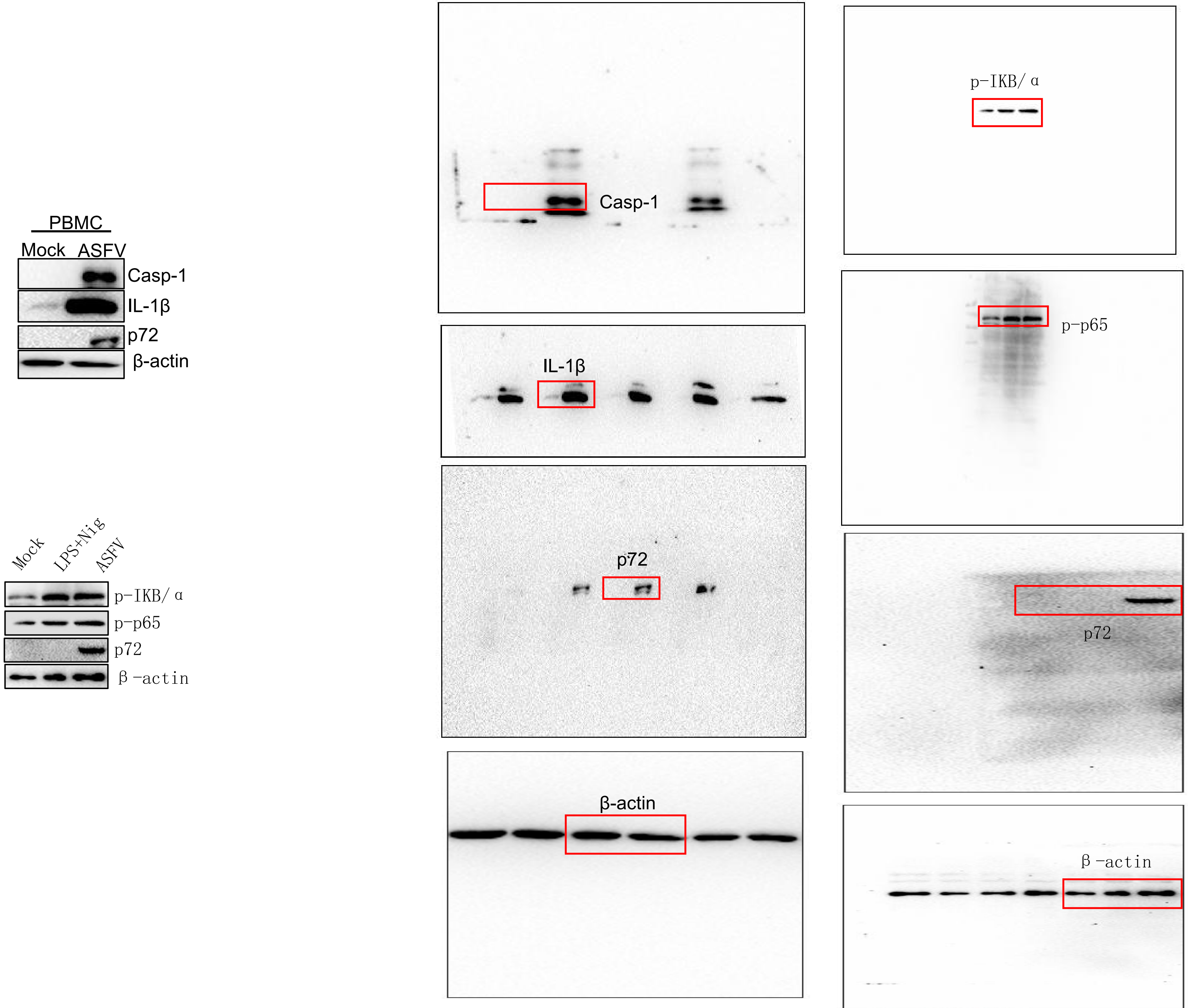

PBMC
Mock
ASFV
Casp-1
IL-1β
p72
β-actin
Mock
LPS+Nig
ASFV
p-IKB/α
p-p65
p72
β-actin
Casp-1
IL-1β
p72
β-actin
p-IKB/α
p-p65
p72
β-actin

Fig3A

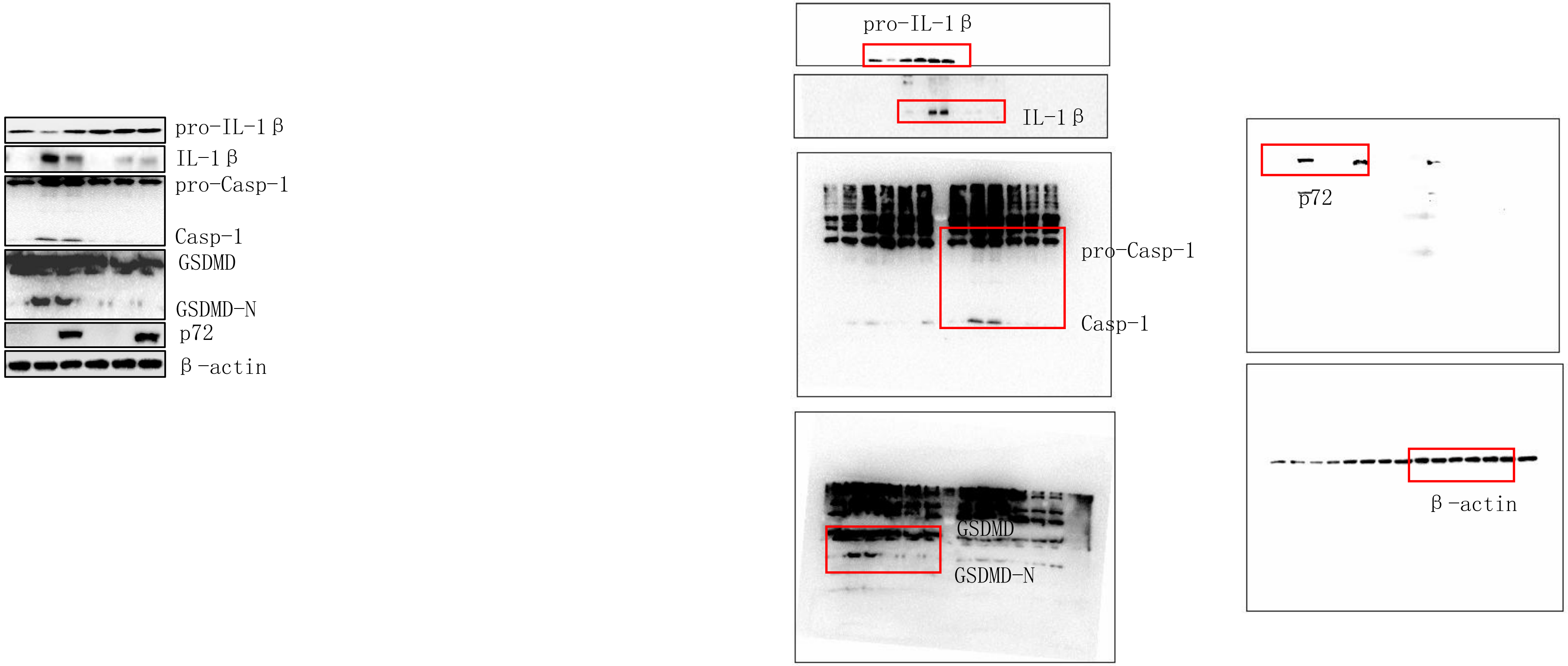
pro-IL-1β
IL-1β
pro-Casp-1
Casp-1
GSDMD
GSDMD-N
p72
β-actin
pro-IL-1β
IL-1β
pro-Casp-1
Casp-1
GSDMD
GSDMD-N
p72
β-actin

Fig3B

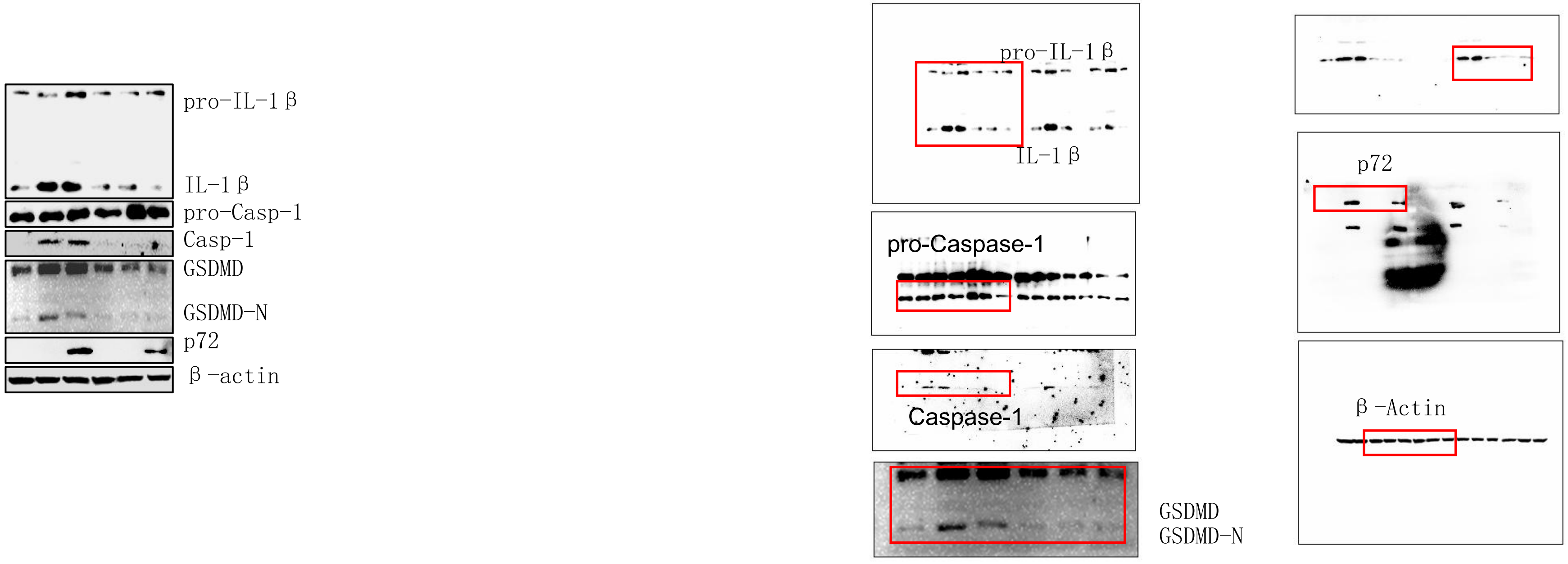
pro-IL-1β
IL-1β
pro-Casp-1
Casp-1
GSDMD
GSDMD-N
p72
β-actin
pro-IL-1β
IL-1β
pro-Caspase-1
Caspase-1
GSDMD
GSDMD-N
p72
β-Actin

Fig3D

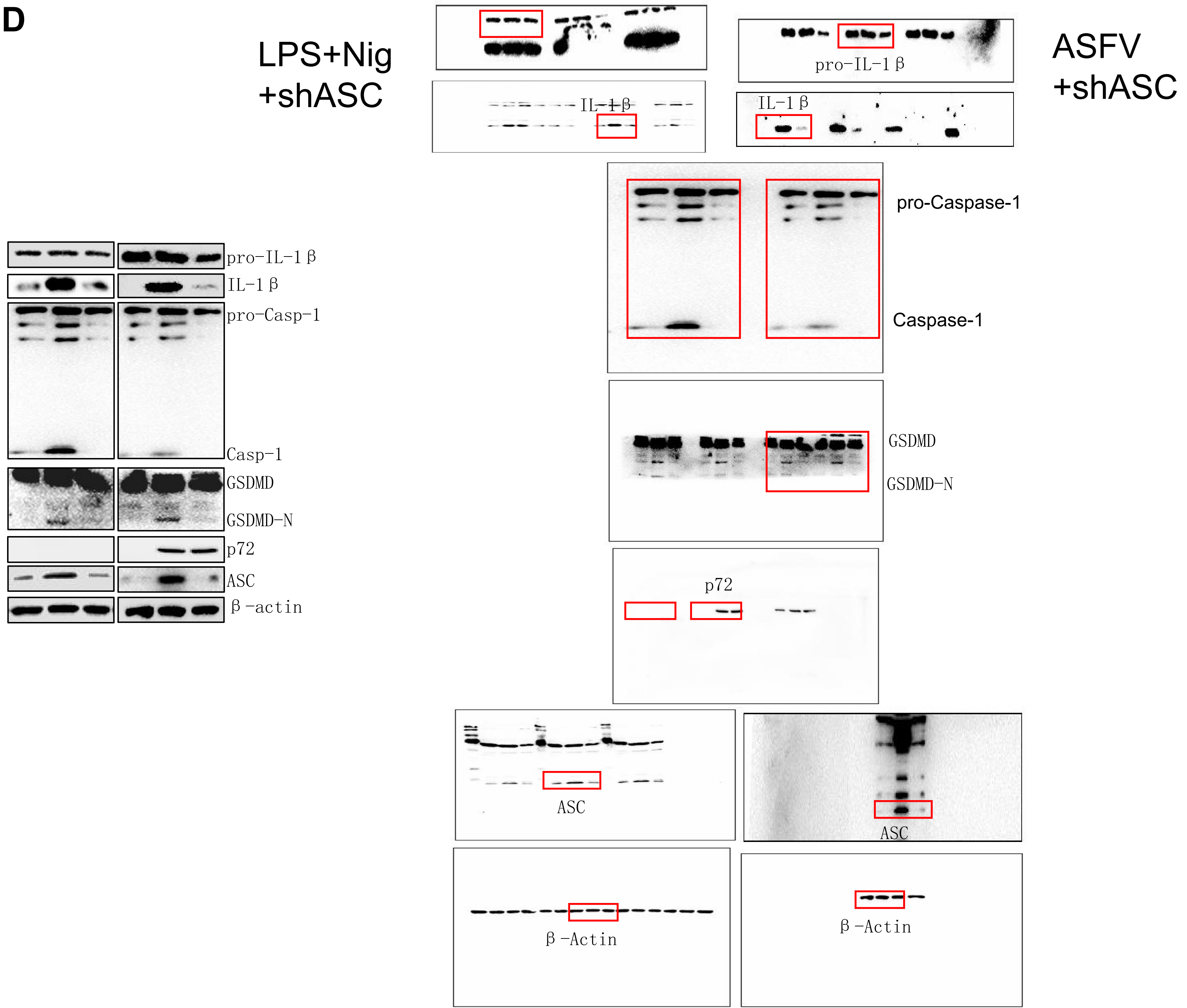
LPS+Nig
+shASC
ASFV
+shASC
pro-IL-1β
IL-1β
pro-Caspase-1
Caspase-1
GSDMD
GSDMD-N
p72
ASC
β-Actin
pro-IL-1β
IL-1β
pro-Casp-1
Casp-1
GSDMD
GSDMD-N
p72
ASC
β-actin

Fig3E

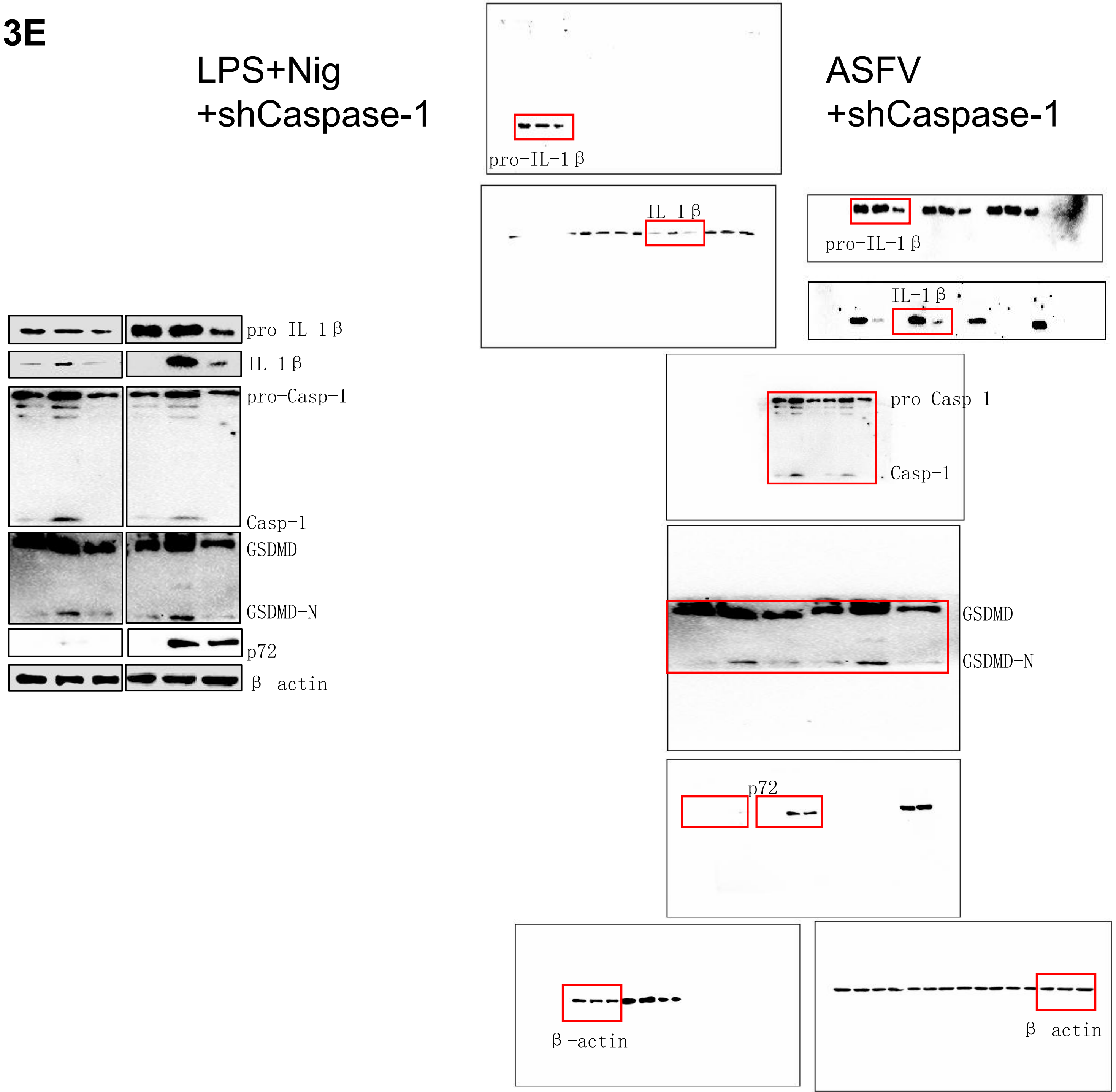
LPS+Nig
+shCaspase-1
ASFV
+shCaspase-1
pro-IL-1β
IL-1β
pro-IL-1β
IL-1β
pro-IL-1β
IL-1β
pro-Casp-1
Casp-1
GSDMD
GSDMD-N
p72
β-actin
pro-Casp-1
Casp-1
GSDMD
GSDMD-N
p72
β-actin
β-actin

Fig3F

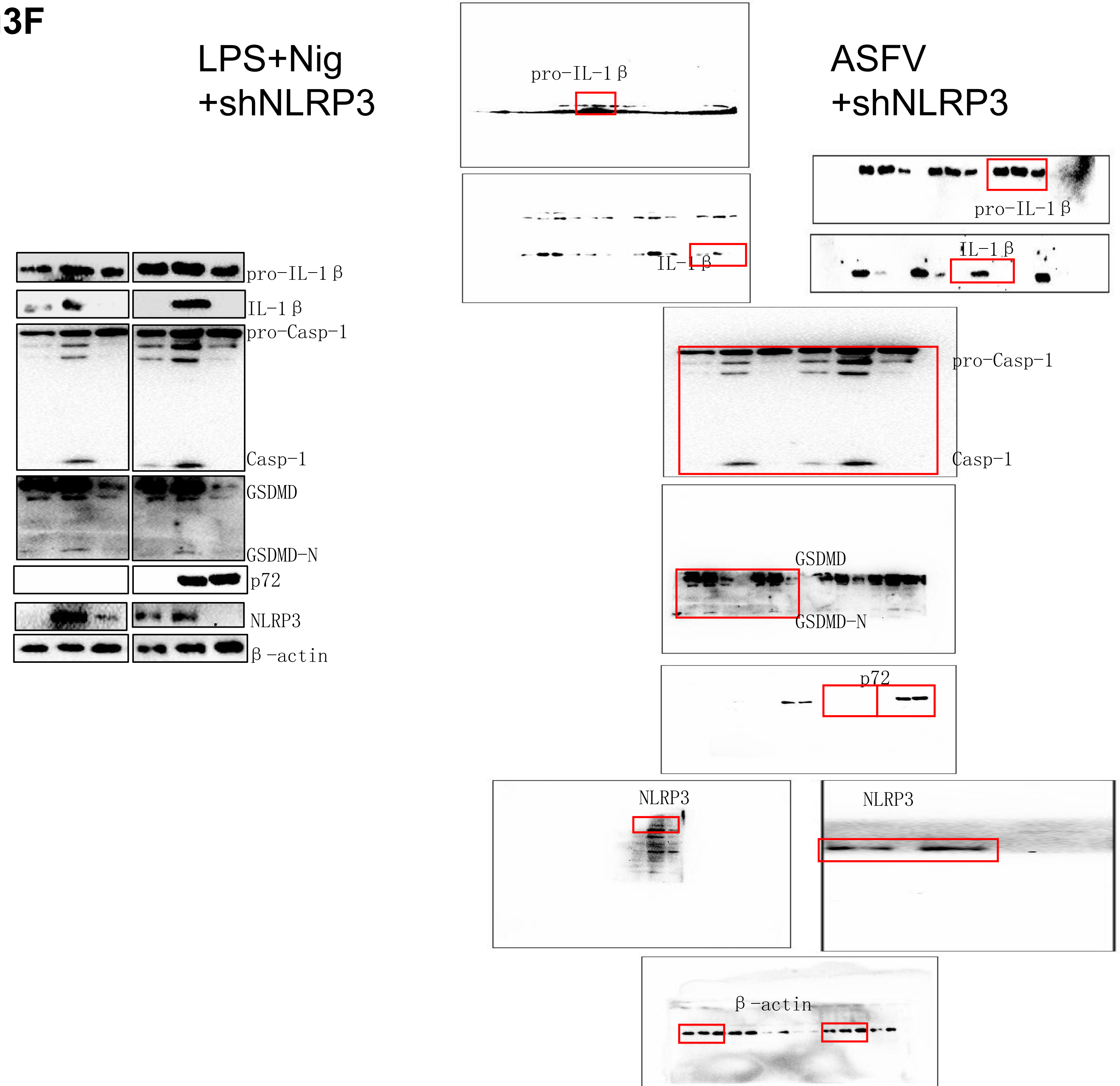
LPS+Nig
+shNLRP3
ASFV
+shNLRP3
pro-IL-1β
IL-1β
pro-Casp-1
Casp-1
GSDMD
GSDMD-N
p72
NLRP3
β-actin

Fig4H

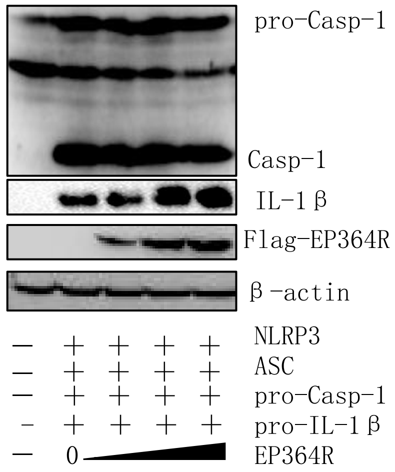
pro-Casp-1
Casp-1
IL-1β
Flag-EP364R
β-actin
NLRP3
ASC
pro-Casp-1
pro-IL-1β
EP364R
0

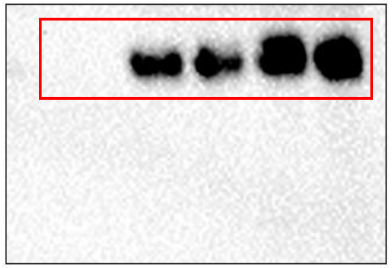

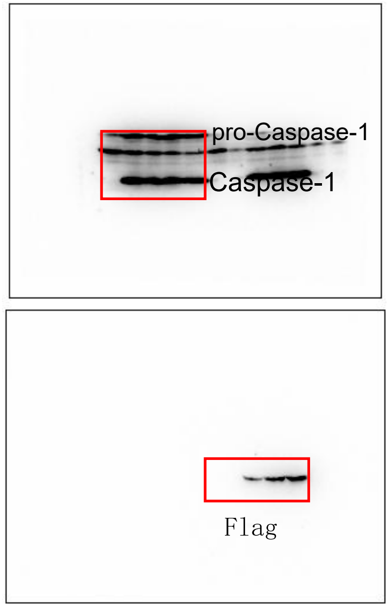
pro-Caspase-1
Caspase-1
Flag

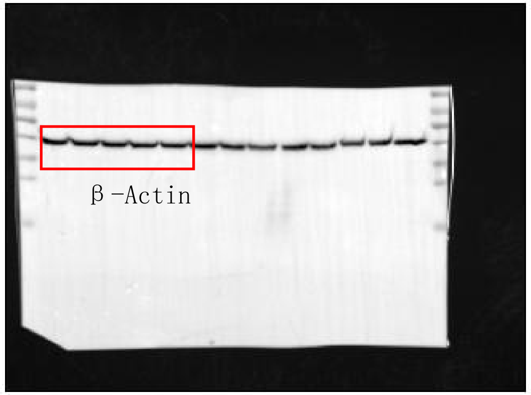
β-Actin

Fig4I

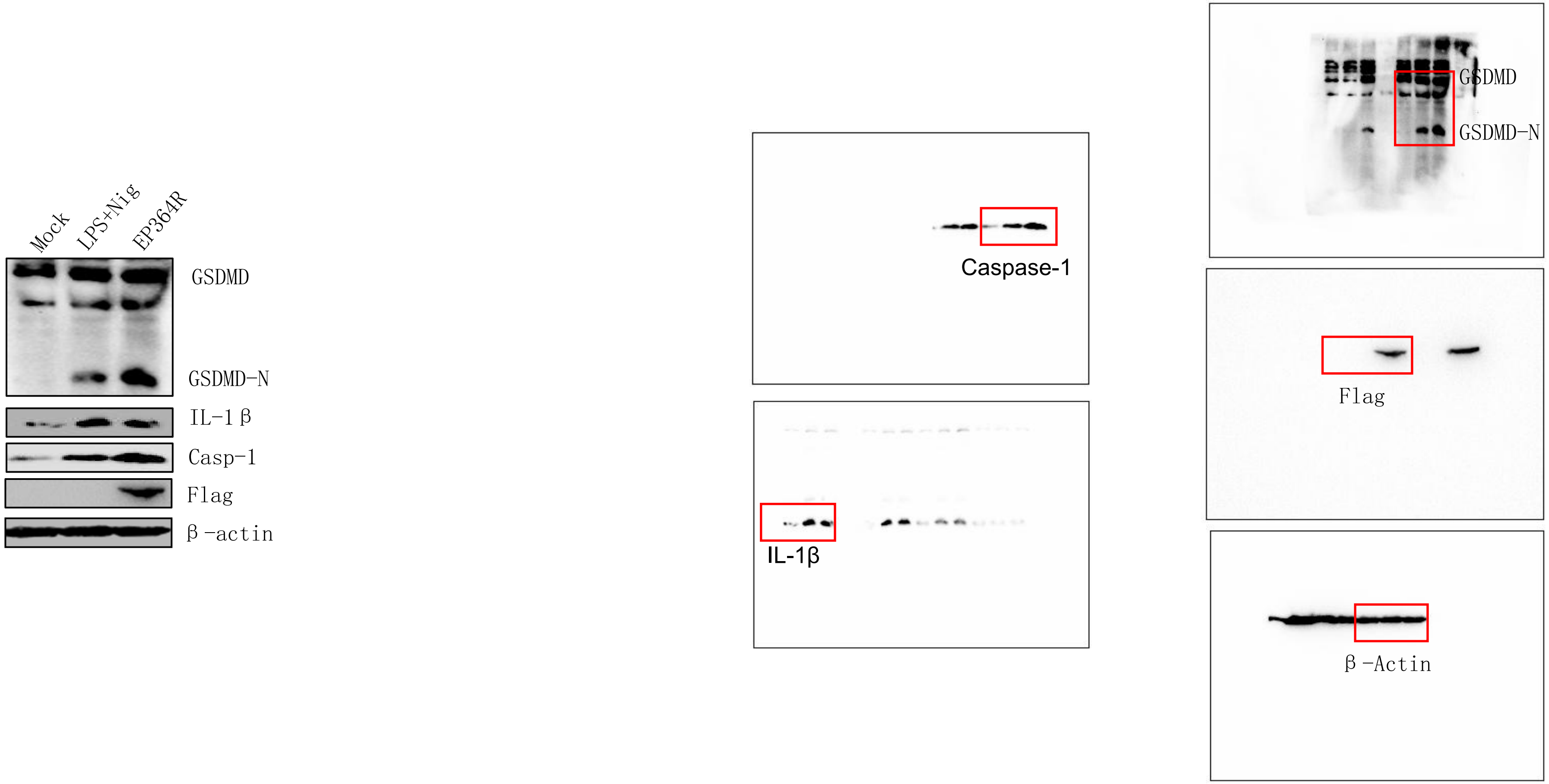
Mock
LPS+Nig
EP364R
GSDMD
GSDMD-N
IL-1β
Casp-1
Flag
β-actin
Caspase-1
IL-1β
GSDMD
GSDMD-N
Flag
β-Actin

Fig4J

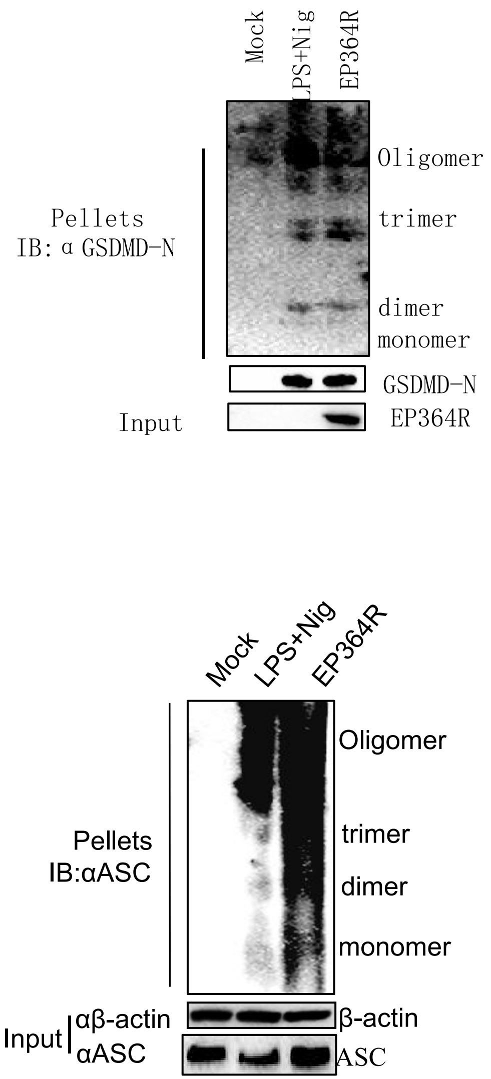
Mock
LPS+Nig
EP364R
Oligomer
trimer
dimer
monomer
Pellets
IB: α GSDMD-N
GSDMD-N
Input
EP364R
Mock
LPS+Nig
EP364R
Oligomer
trimer
dimer
monomer
Pellets
IB:αASC
Input
αβ-actin
αASC
β-actin
ASC

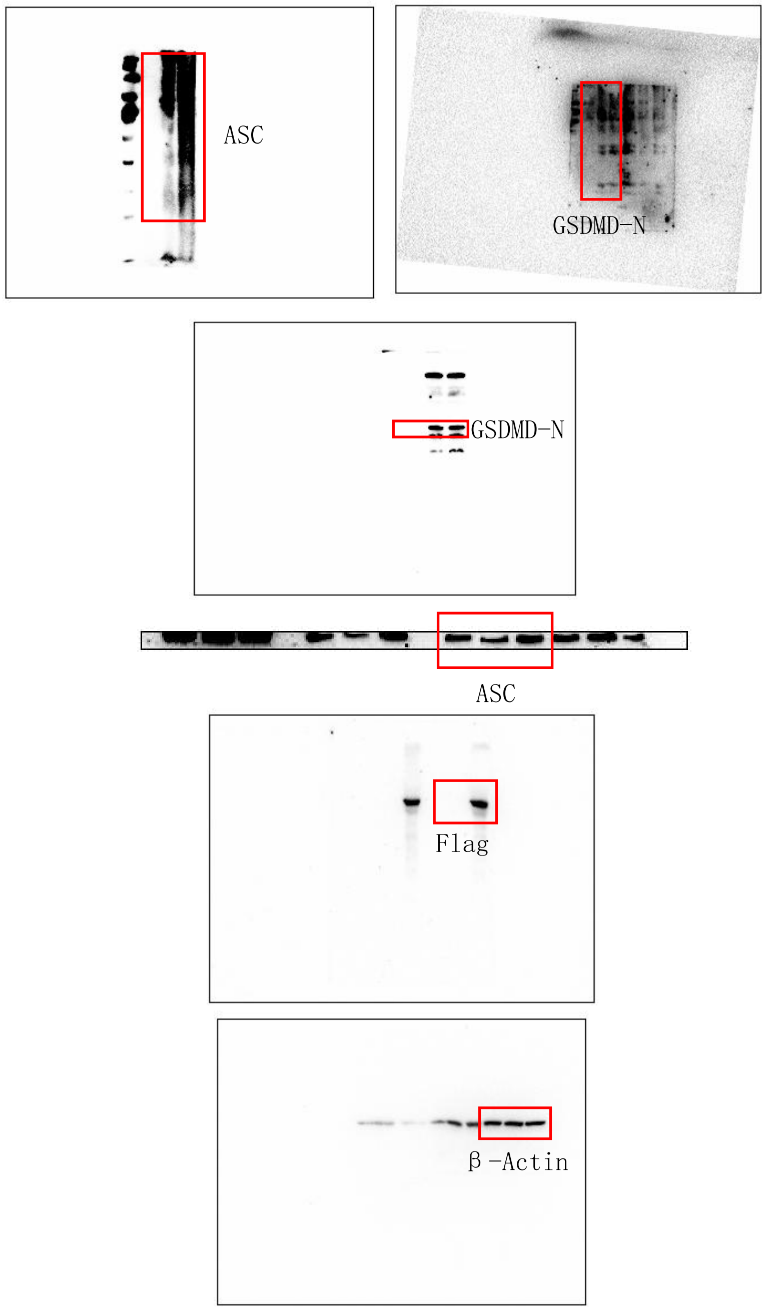
ASC
GSDMD-N
GSDMD-N
ASC
Flag
β -Actin

Fig5A,E

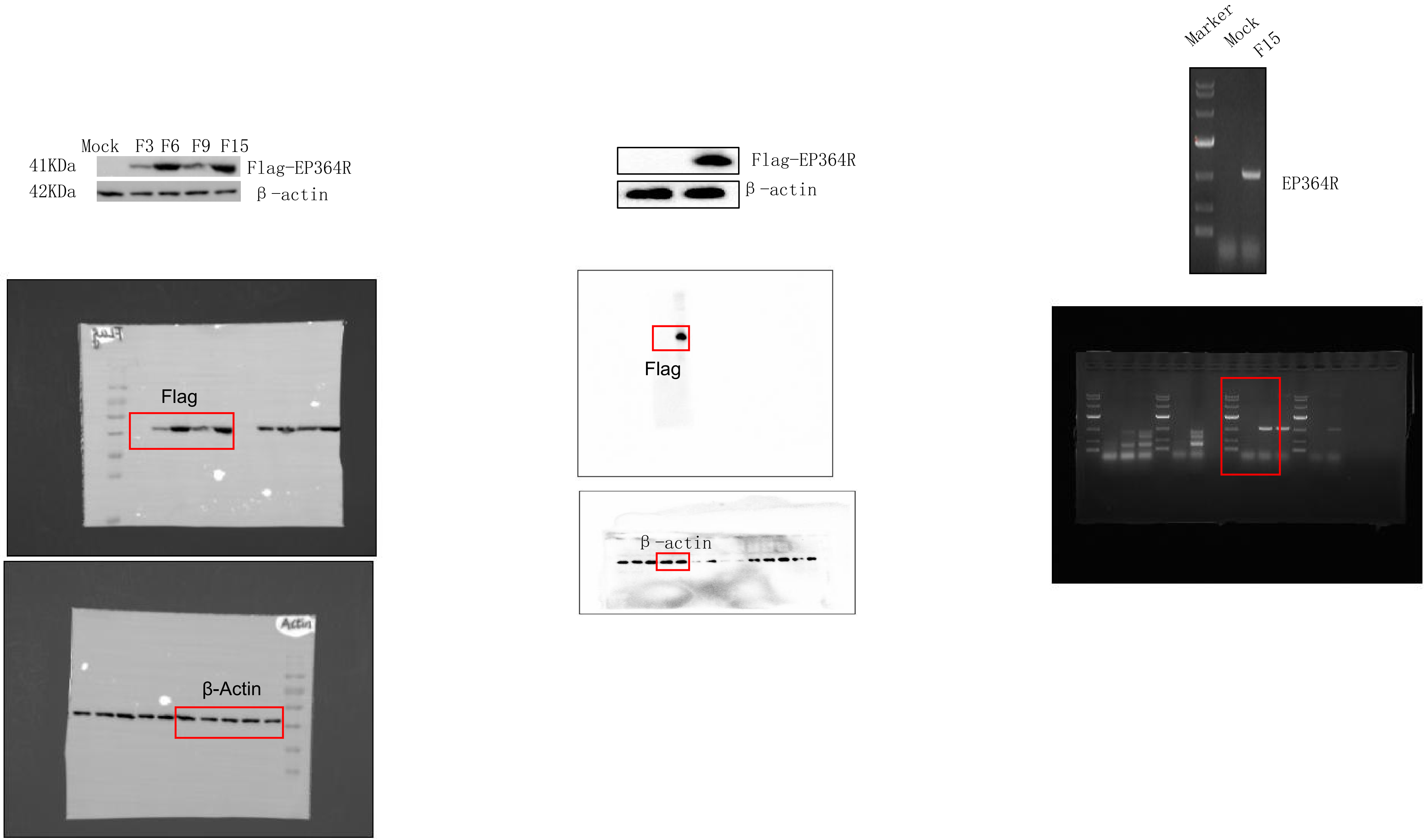

Mock F3 F6 F9 F15
41KDa
42KDa
Flag-EP364R
β -actin
Flag
β-Actin
Actin
Flag-EP364R
β -actin
Flag
β -actin
Marker
Mock
F15
EP364R

Fig5B

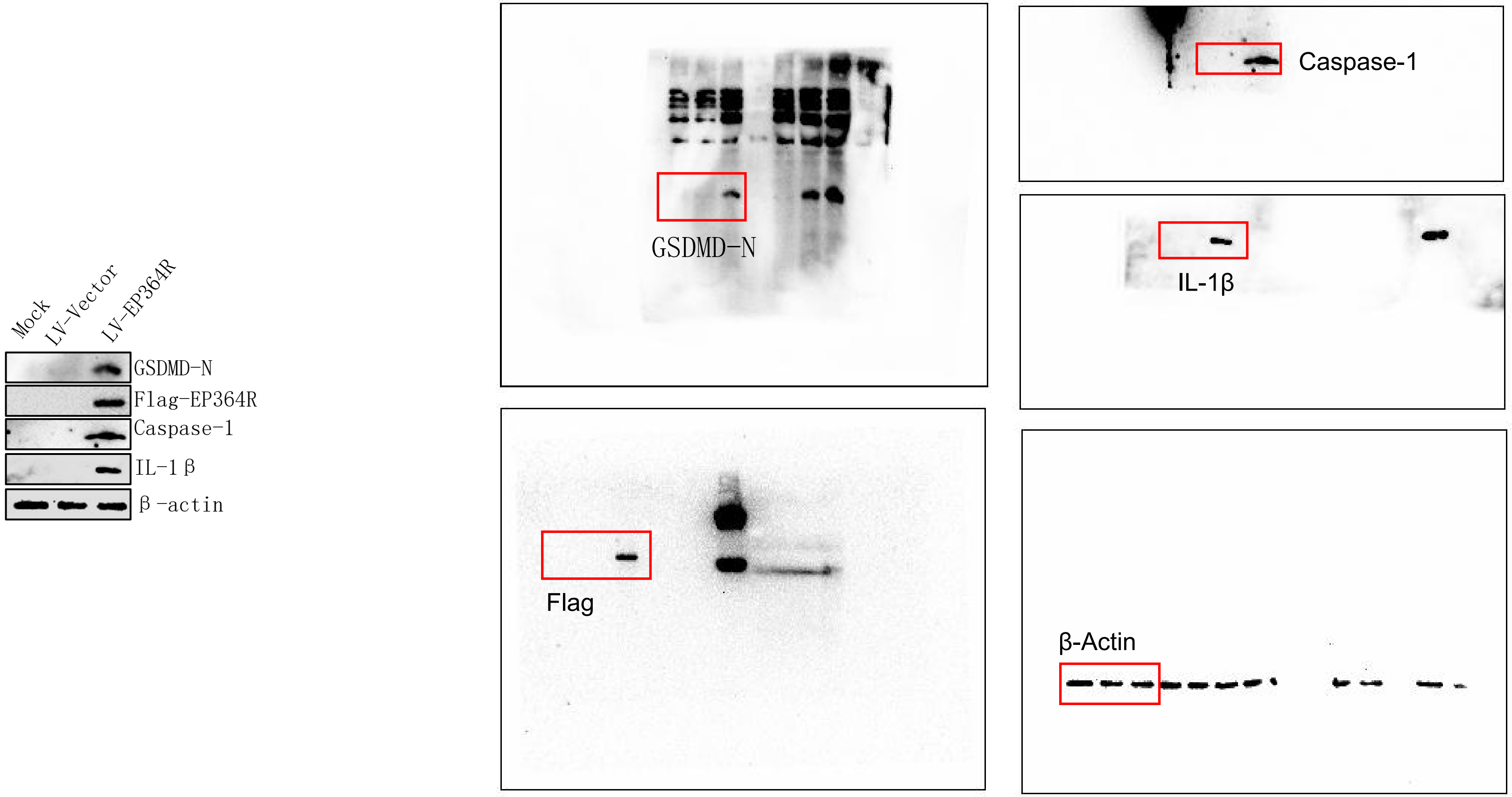
Mock
LV-Vector
LV-EP364R
GSDMD-N
Flag-EP364R
Caspase-1
IL-1 β
β -actin
GSDMD-N
Caspase-1
IL-1β
Flag
β-Actin

Fig5G

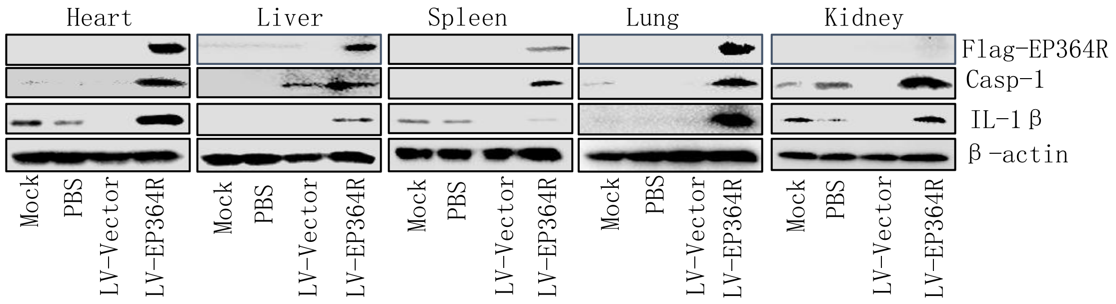

Heart
Liver
Spleen
Lung
Kidney
Flag-EP364R
Casp-1
IL-1β
β-actin
Mock
PBS
LV-Vector
LV-EP364R

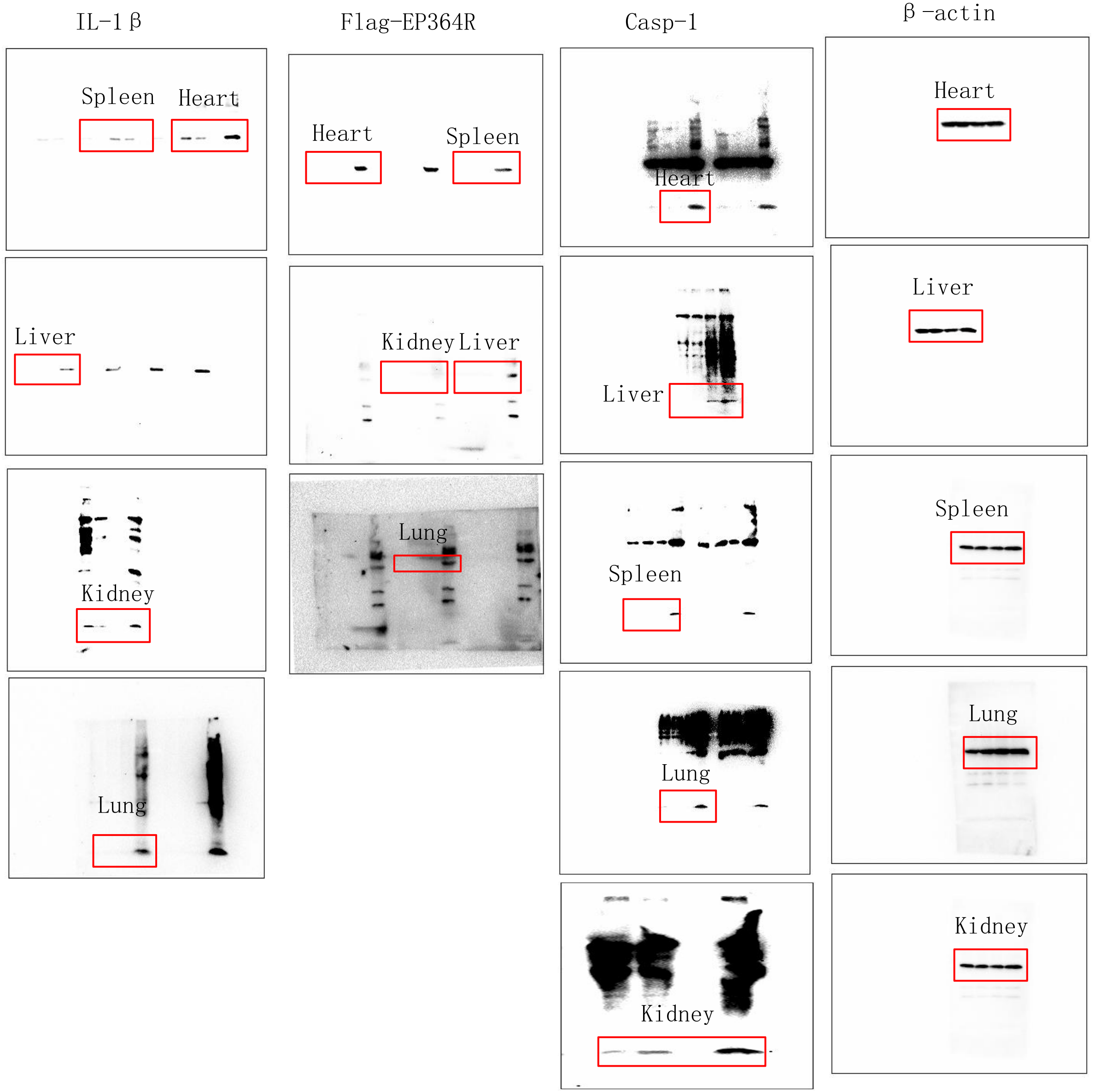

IL-1β
Flag-EP364R
Casp-1
β-actin
Spleen
Heart
Liver
Kidney
Lung

Fig6A

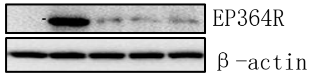

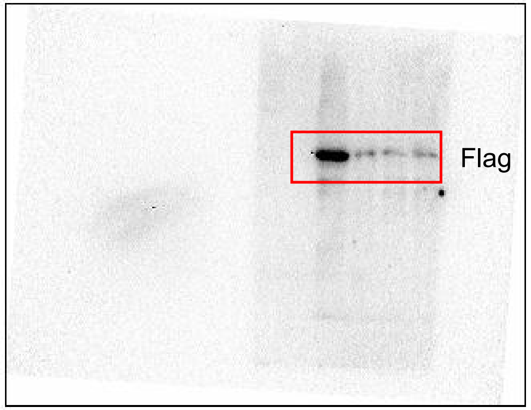

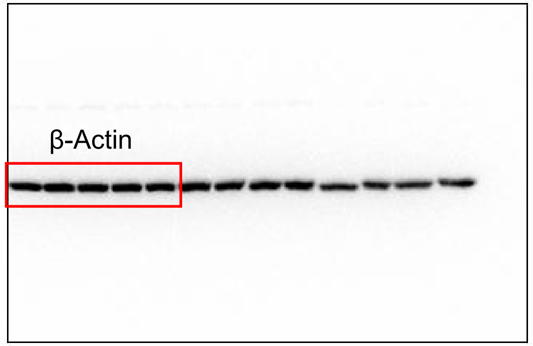

Fig6C

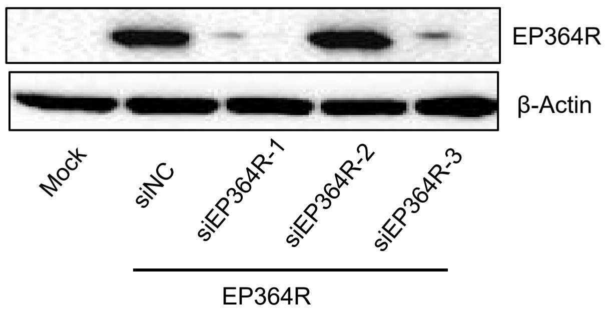
EP364R
β-Actin
Mock
siNC
siEP364R-1
siEP364R-2
siEP364R-3
EP364R

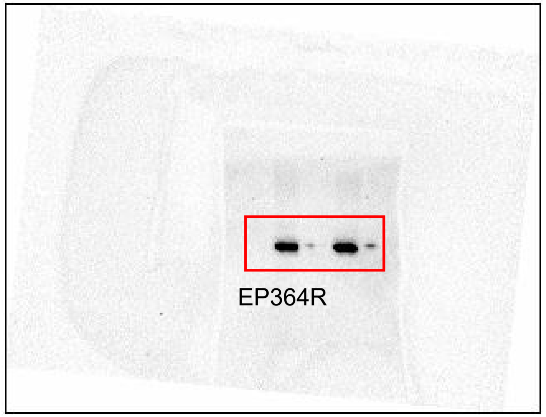
EP364R

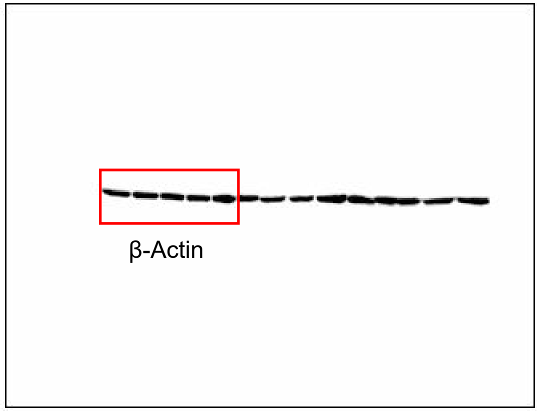
β-Actin

Fig6D

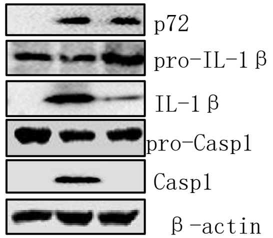

p72
pro-IL-1β
IL-1β
pro-Casp1
Casp1
β-actin

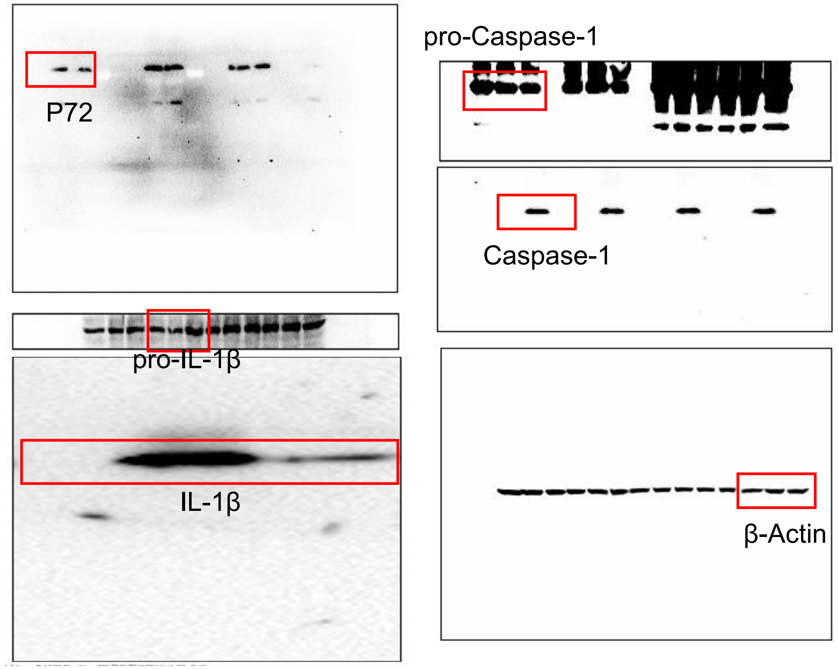

P72
pro-IL-1β
IL-1β
pro-Caspase-1
Caspase-1
β-Actin

Fig6I

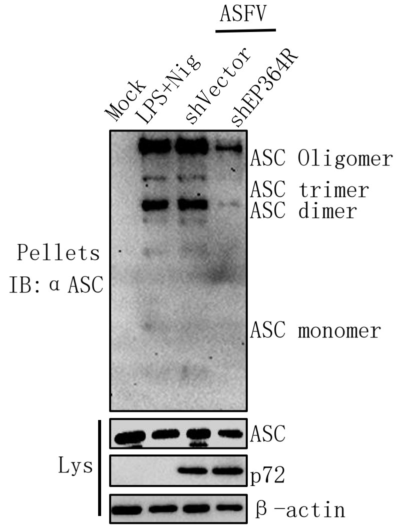
ASFV
Mock
LPS+Nig
shVector
shEP364R
ASC Oligomer
ASC trimer
ASC dimer
Pellets
IB: α ASC
ASC monomer
Lys
ASC
p72
β -actin

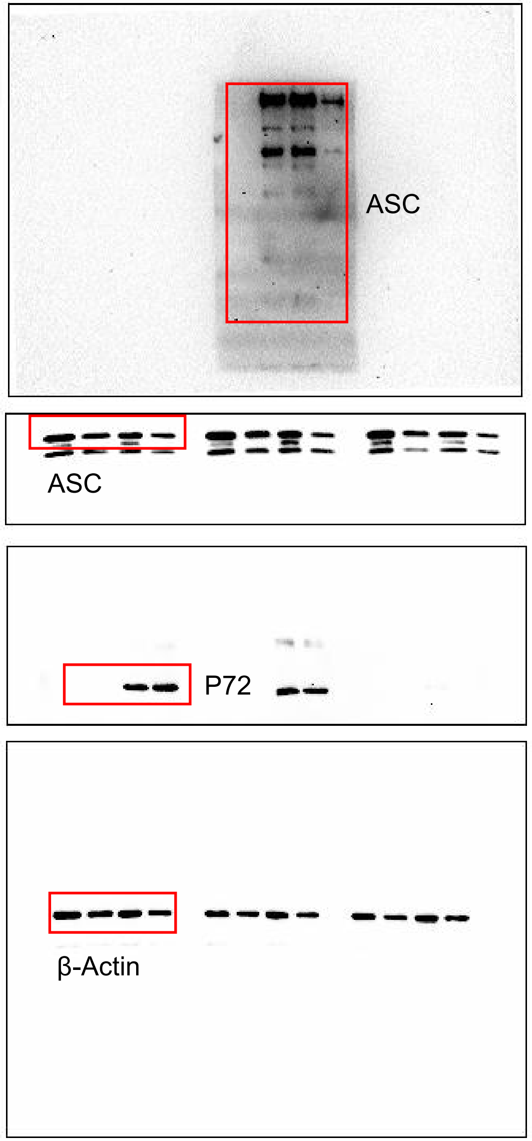
ASC
ASC
P72
β-Actin

Fig7A

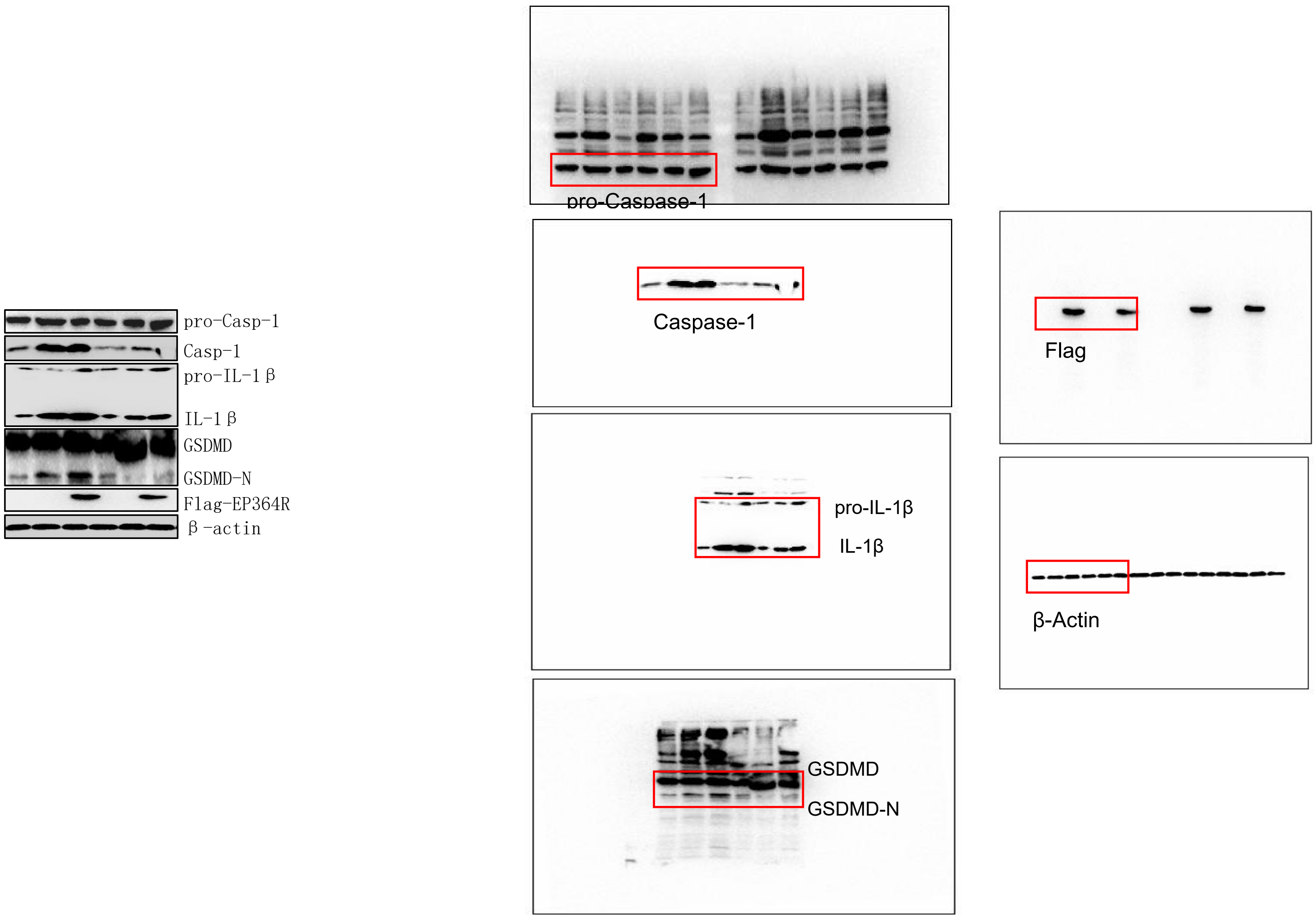
pro-Casp-1
Casp-1
pro-IL-1β
IL-1β
GSDMD
GSDMD-N
Flag-EP364R
β-actin
pro-Caspase-1
Caspase-1
pro-IL-1β
IL-1β
GSDMD
GSDMD-N
Flag
β-Actin

Fig7B

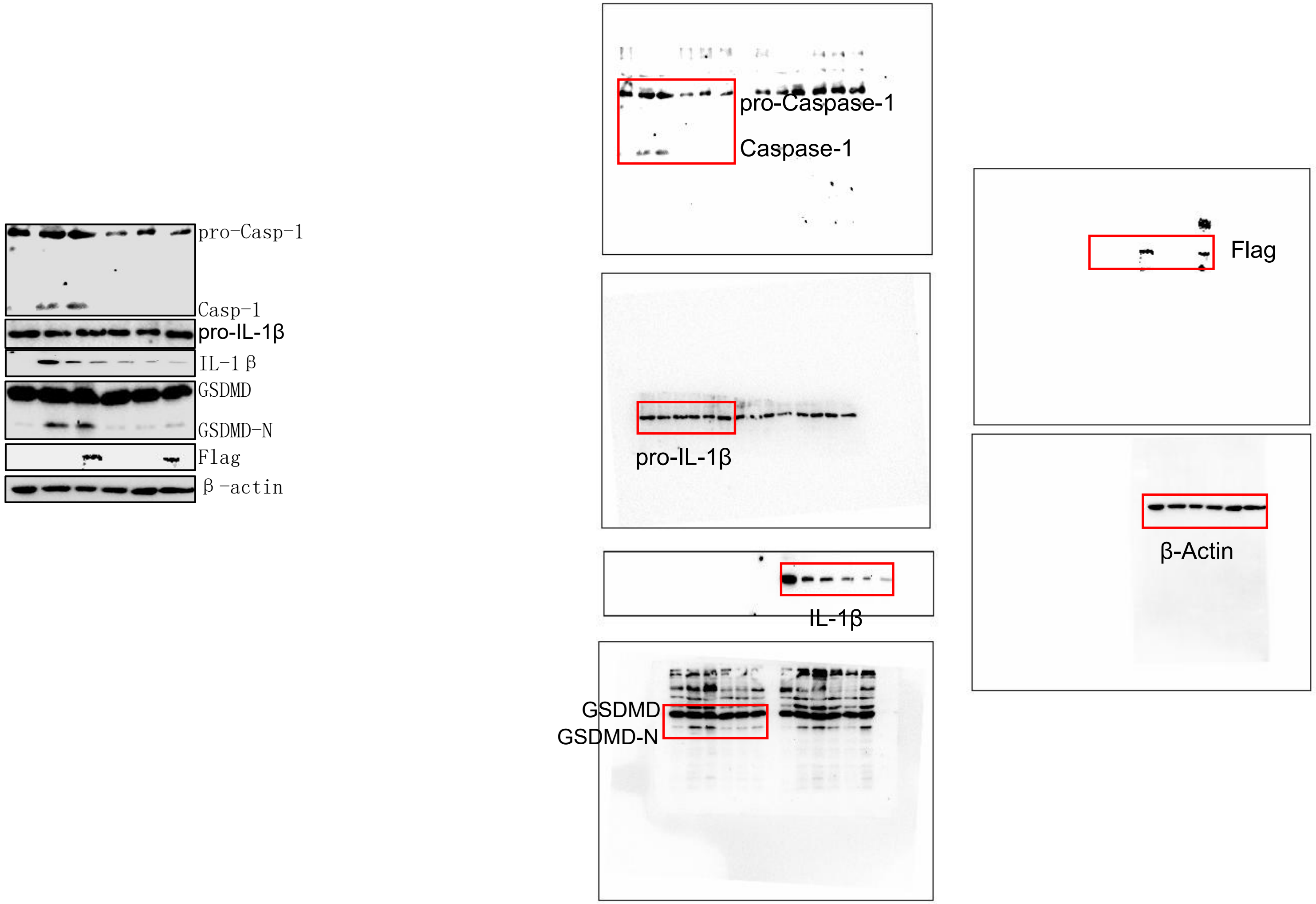
pro-Casp-1
Casp-1
pro-IL-1β
IL-1 β
GSDMD
GSDMD-N
Flag
β -actin
pro-Caspase-1
Caspase-1
pro-IL-1β
IL-1β
GSDMD
GSDMD-N
Flag
β-Actin

Fig7C

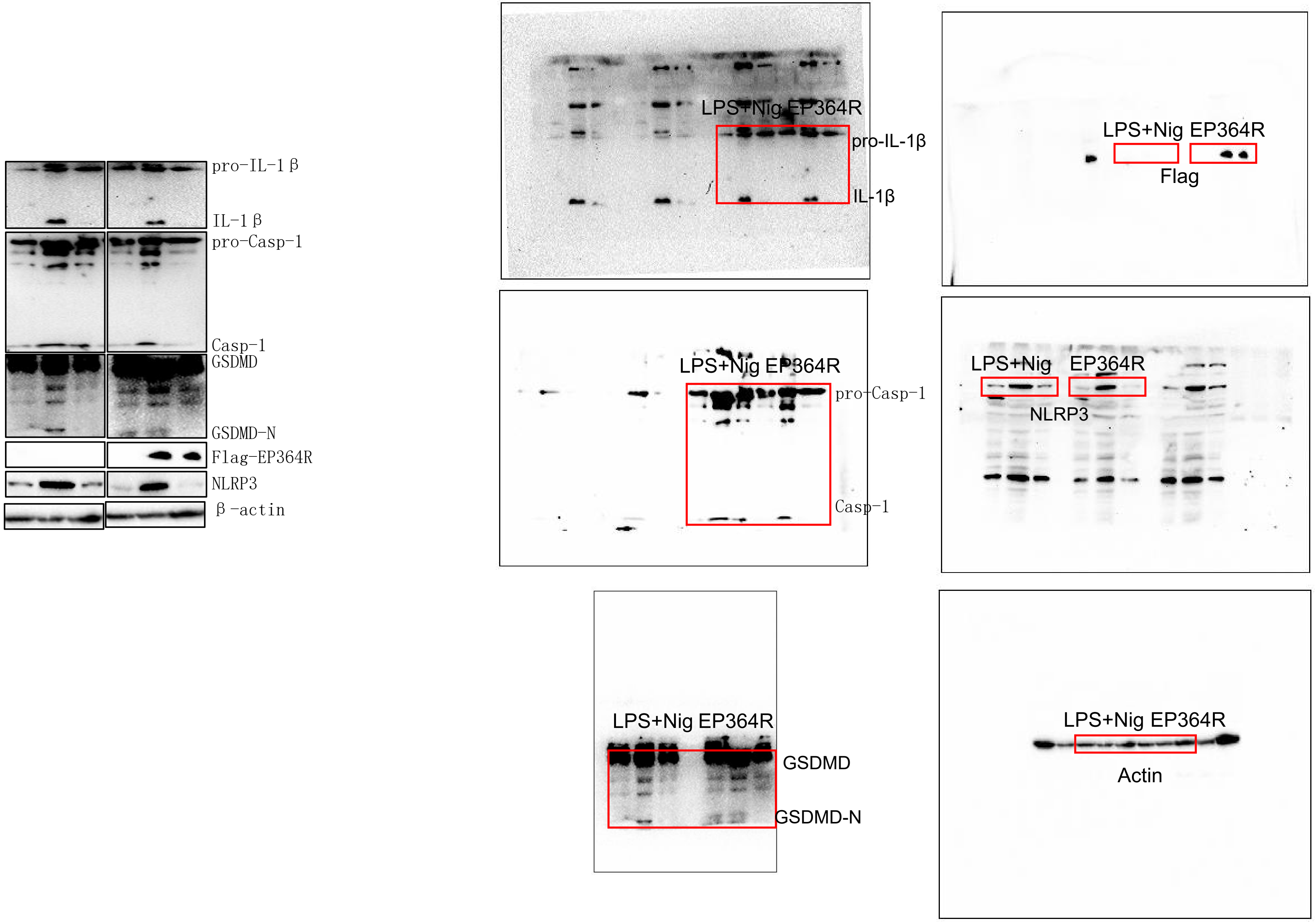
pro-IL-1β
IL-1β
pro-Casp-1
Casp-1
GSDMD
GSDMD-N
Flag-EP364R
NLRP3
β-actin
LPS+Nig EP364R
pro-IL-1β
IL-1β
LPS+Nig EP364R
Flag
LPS+Nig EP364R
pro-Casp-1
Casp-1
LPS+Nig
EP364R
NLRP3
LPS+Nig EP364R
GSDMD
GSDMD-N
LPS+Nig EP364R
Actin

Fig7D

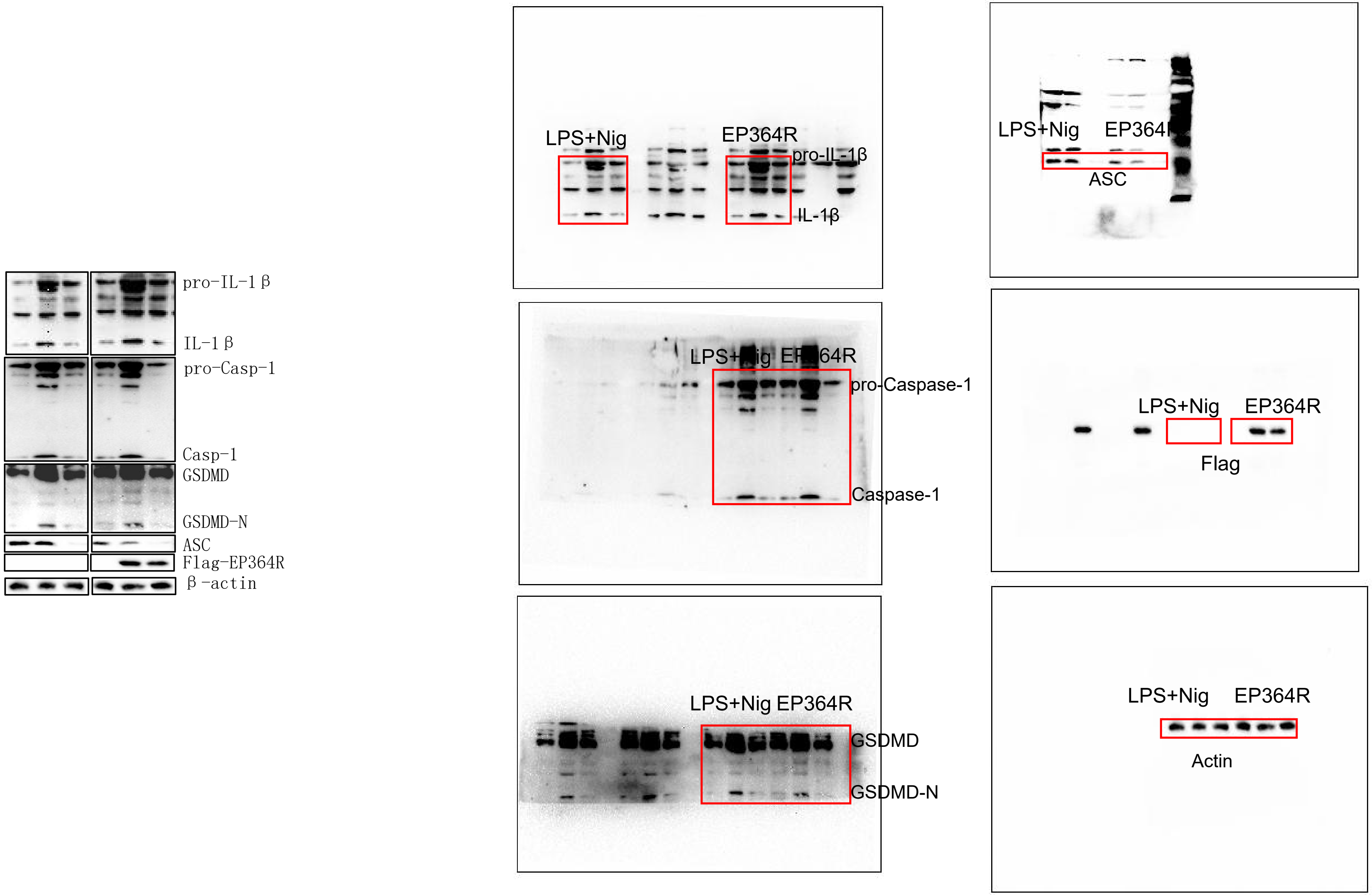
pro-IL-1β
IL-1β
pro-Casp-1
Casp-1
GSDMD
GSDMD-N
ASC
Flag-EP364R
β-actin
LPS+Nig
EP364R
pro-IL-1β
IL-1β
LPS+Nig
EP364
ASC
LPS+Nig EP364R
pro-Caspase-1
Caspase-1
LPS+Nig
EP364R
Flag
LPS+Nig EP364R
GSDMD
GSDMD-N
LPS+Nig
EP364R
Actin

Fig7E

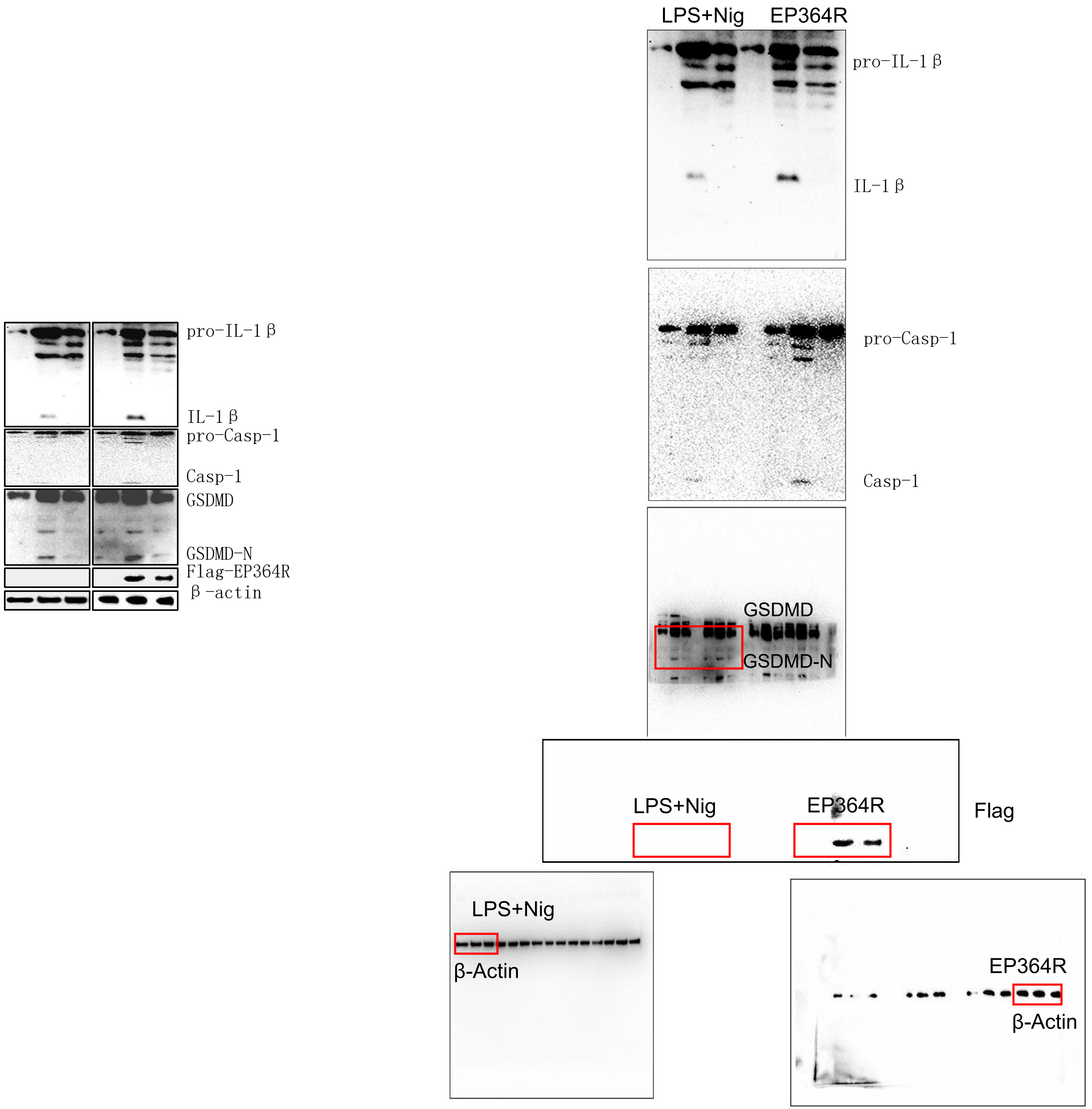
pro-IL-1β
IL-1β
pro-Casp-1
Casp-1
GSDMD
GSDMD-N
Flag-EP364R
β-actin
LPS+Nig
EP364R
pro-IL-1β
IL-1β
pro-Casp-1
Casp-1
GSDMD
GSDMD-N
LPS+Nig
EP364R
Flag
LPS+Nig
β-Actin
EP364R
β-Actin

Fig7H

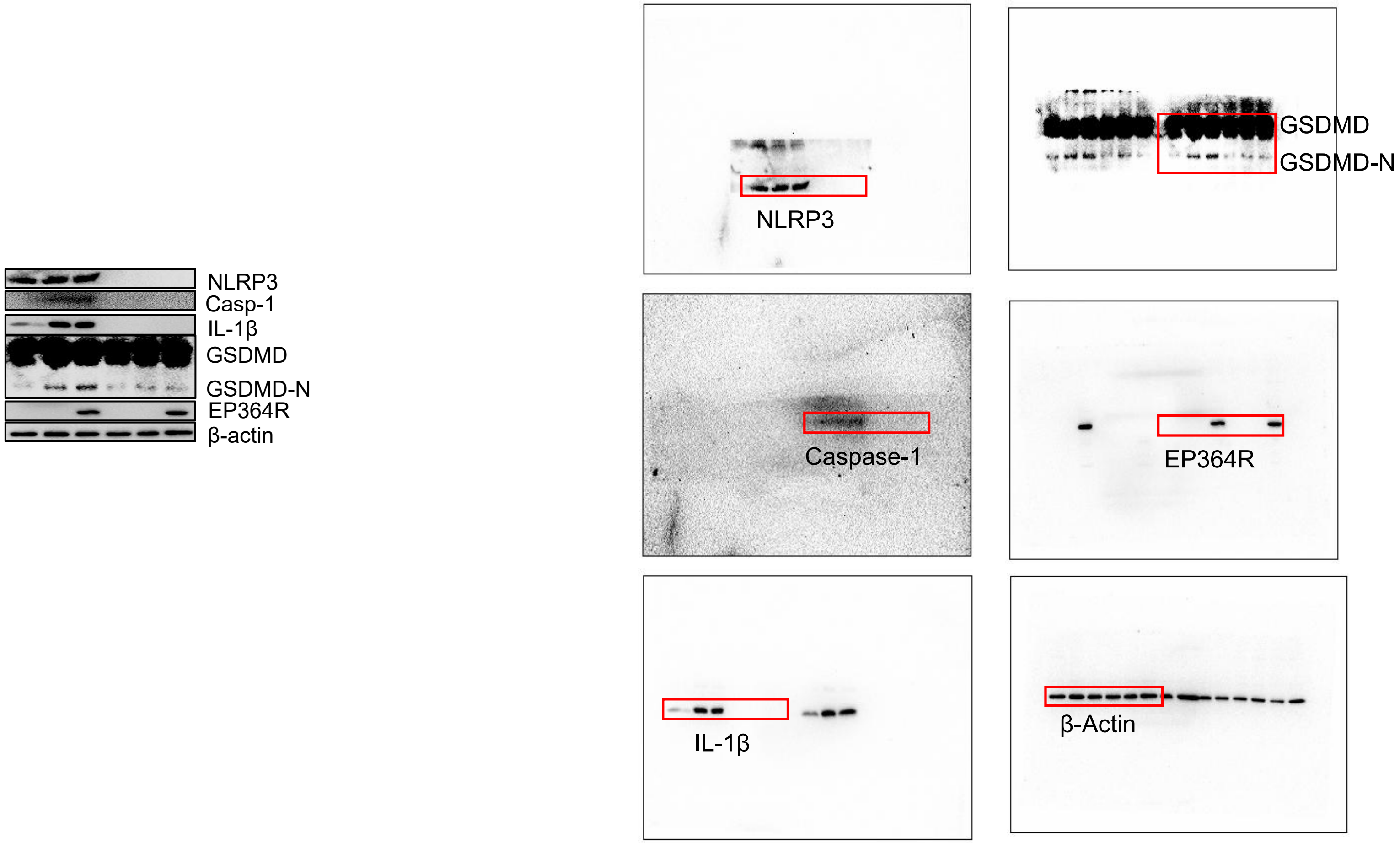
NLRP3
Casp-1
IL-1β
GSDMD
GSDMD-N
EP364R
β-actin
NLRP3
GSDMD
GSDMD-N
Caspase-1
EP364R
IL-1β
β-Actin

Fig8B

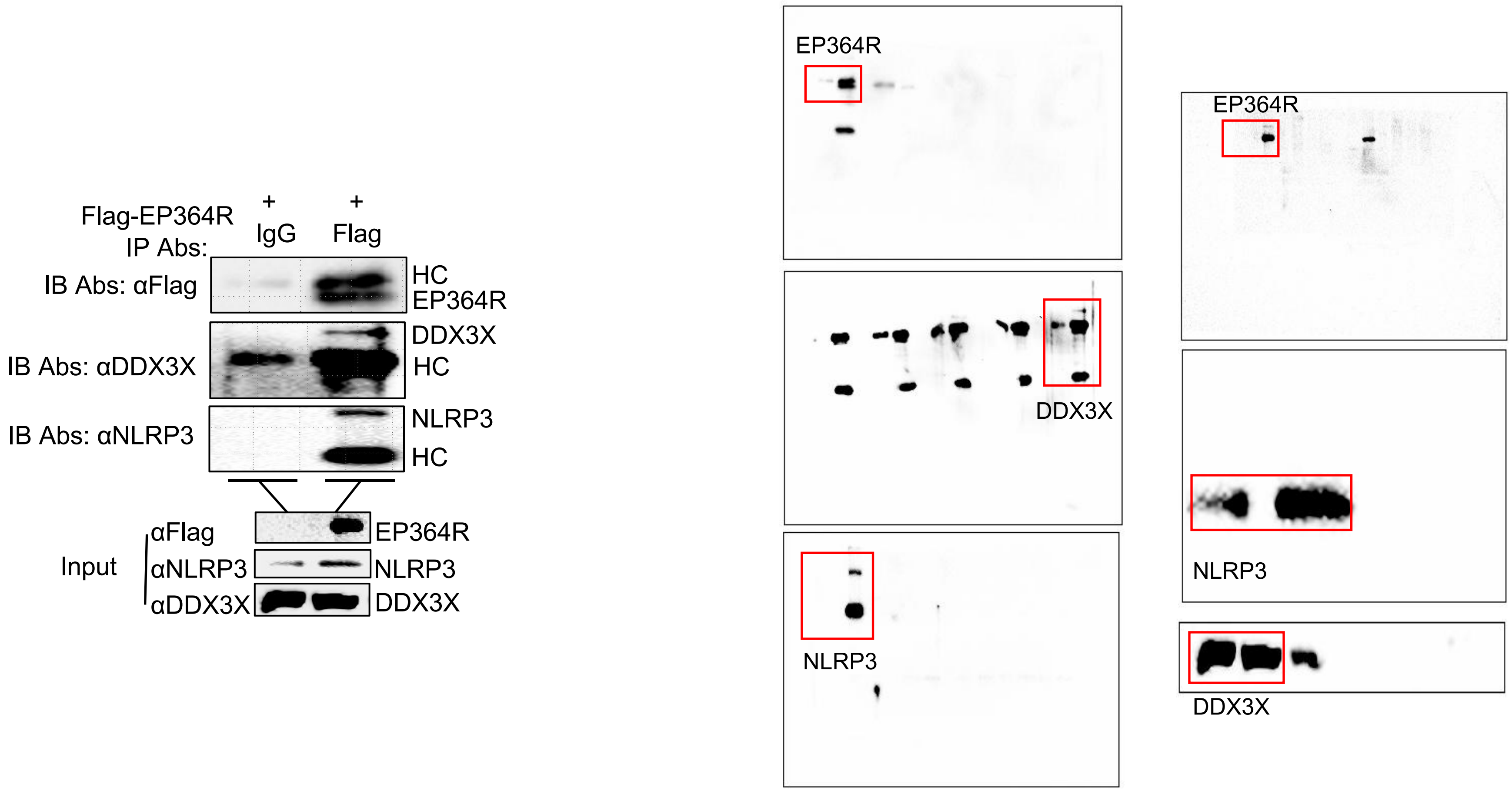

Flag-EP364R
+
+
IP Abs:
IgG
Flag
IB Abs: αFlag
HC
EP364R
IB Abs: αDDX3X
DDX3X
HC
IB Abs: αNLRP3
NLRP3
HC
Input
αFlag
EP364R
αNLRP3
NLRP3
αDDX3X
DDX3X
EP364R
DDX3X
NLRP3
EP364R
NLRP3
DDX3X

Fig8C

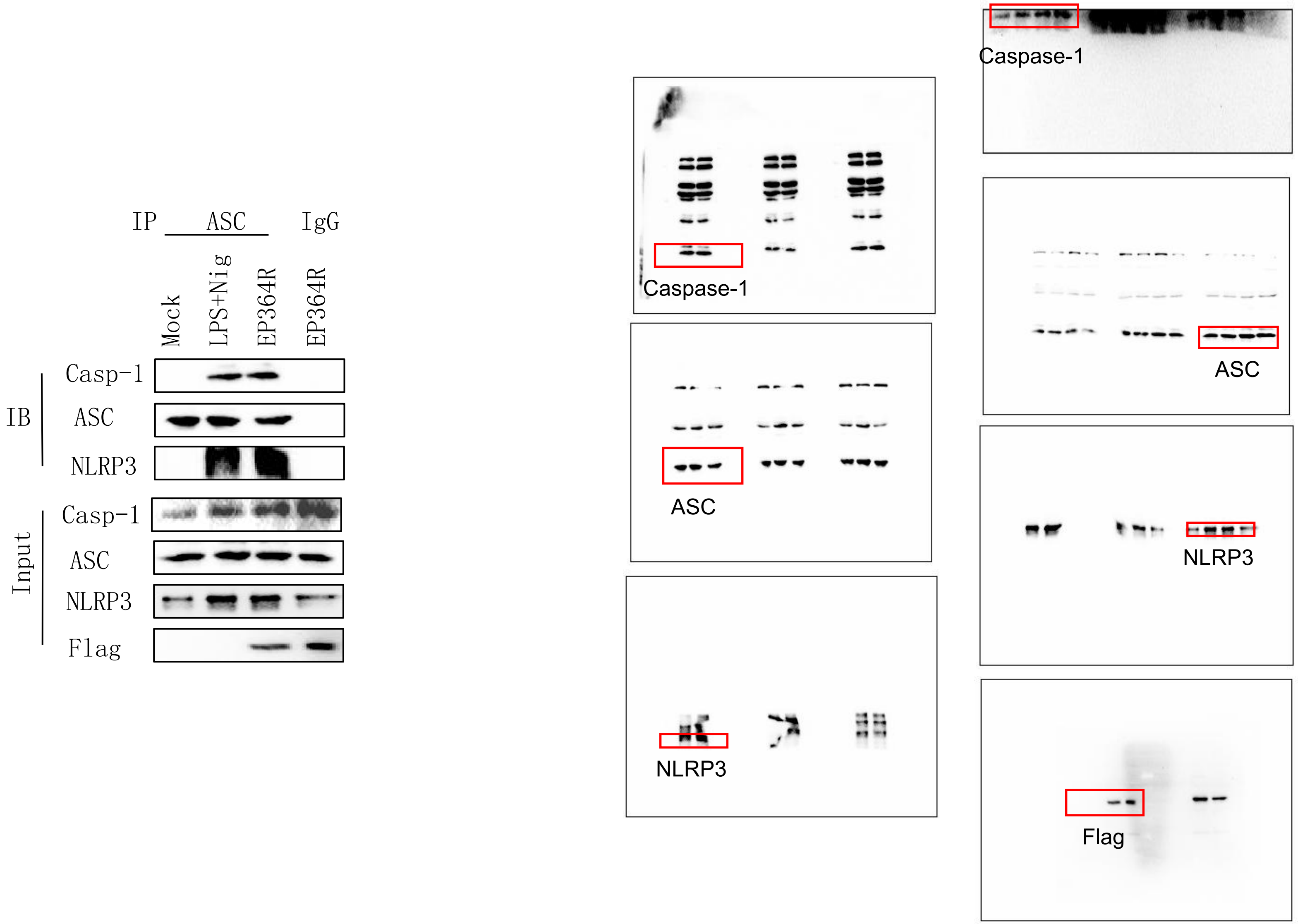

IP
ASC
IgG
Mock
LPS+Nig
EP364R
EP364R
IB
Casp-1
ASC
NLRP3
Input
Casp-1
ASC
NLRP3
Flag
Caspase-1
Caspase-1
ASC
ASC
NLRP3
NLRP3
Flag

Fig8D

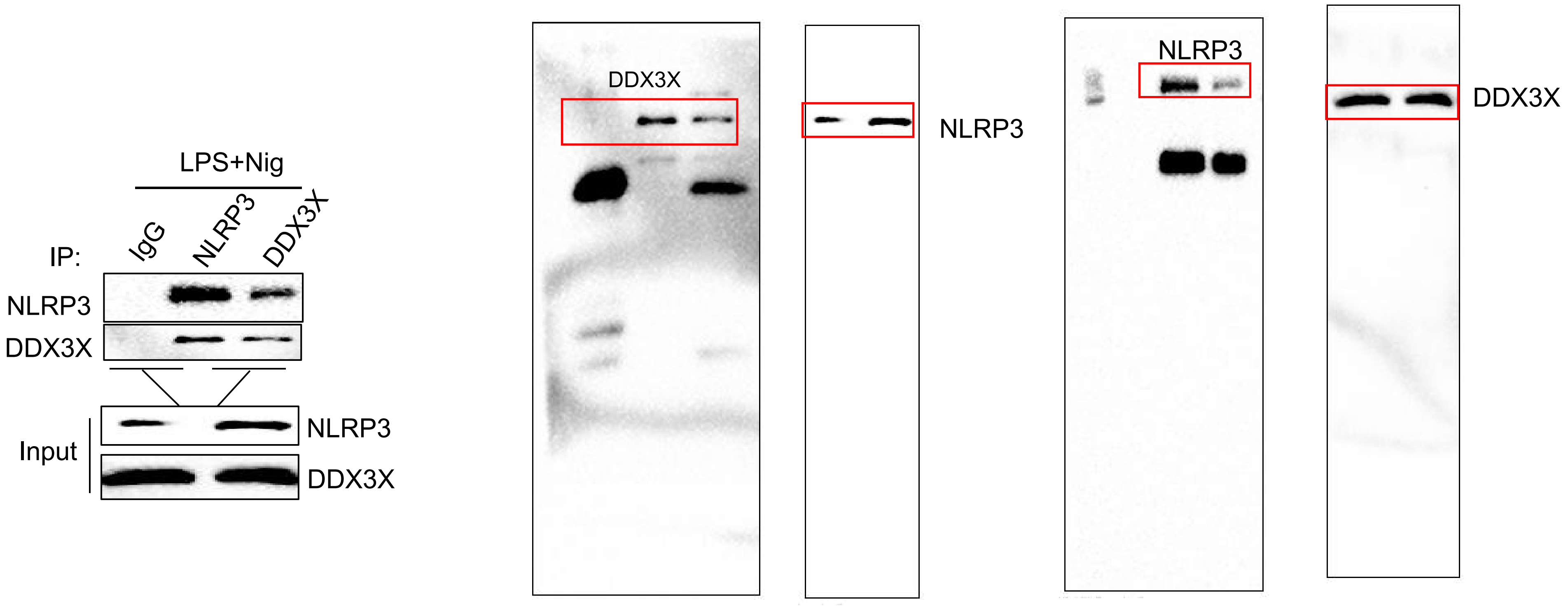
LPS+Nig
IP:
IgG
NLRP3
DDX3X
NLRP3
DDX3X
Input
NLRP3
DDX3X
DDX3X
NLRP3
NLRP3
DDX3X

Fig8E

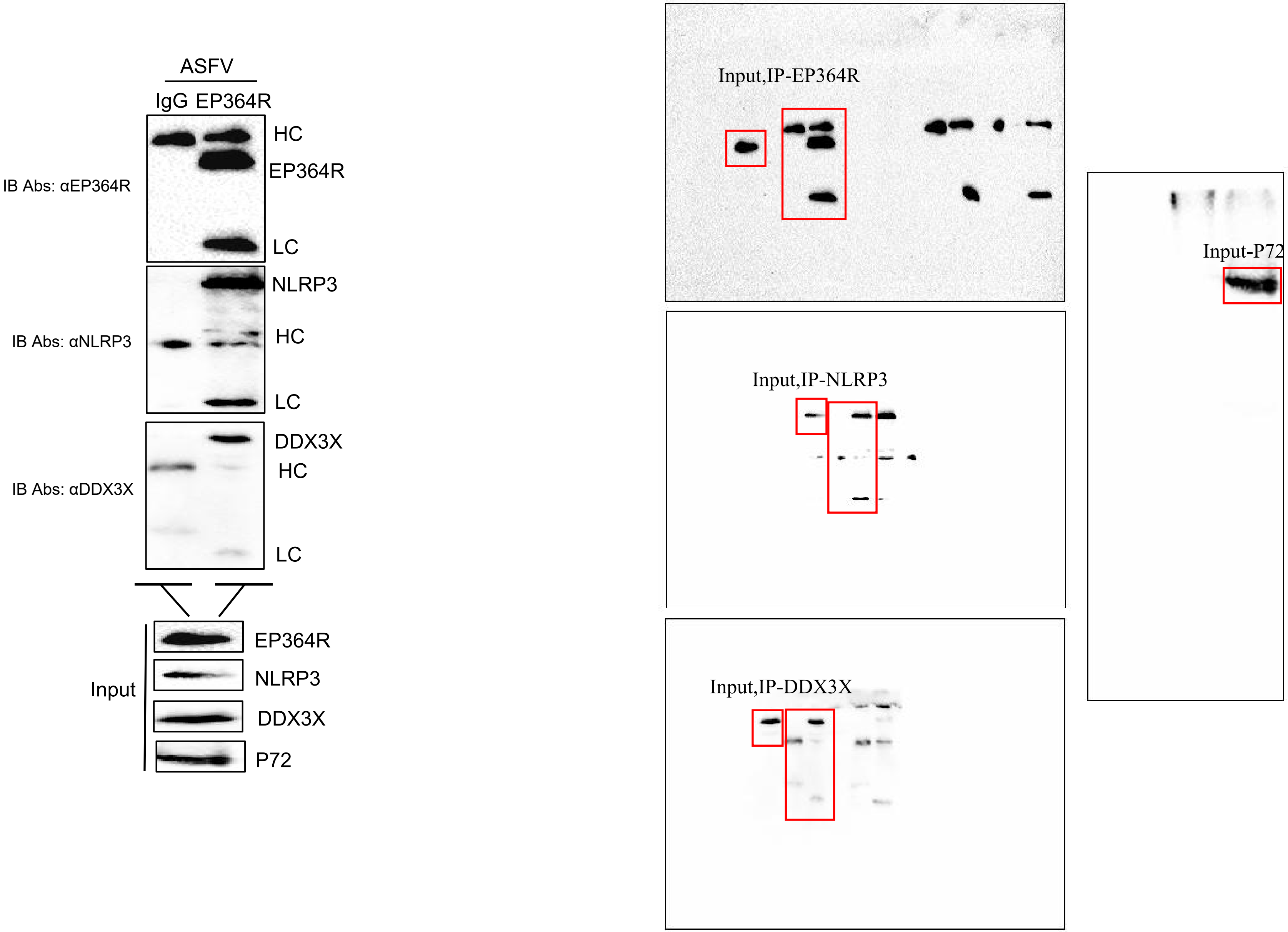
ASFV
IgG EP364R
HC
EP364R
LC
IB Abs: αEP364R
NLRP3
HC
LC
IB Abs: αNLRP3
DDX3X
HC
LC
IB Abs: αDDX3X
Input
EP364R
NLRP3
DDX3X
P72
Input,IP-EP364R
Input,IP-NLRP3
Input,IP-DDX3X
Input-P72

Fig9A

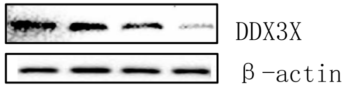

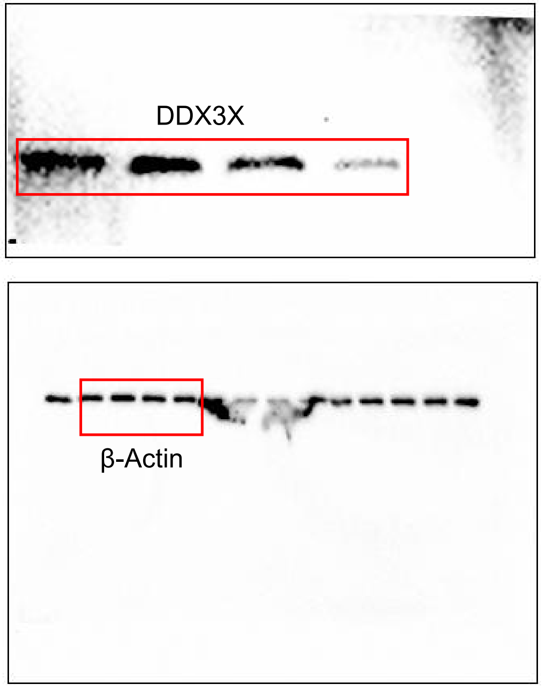

Fig9C

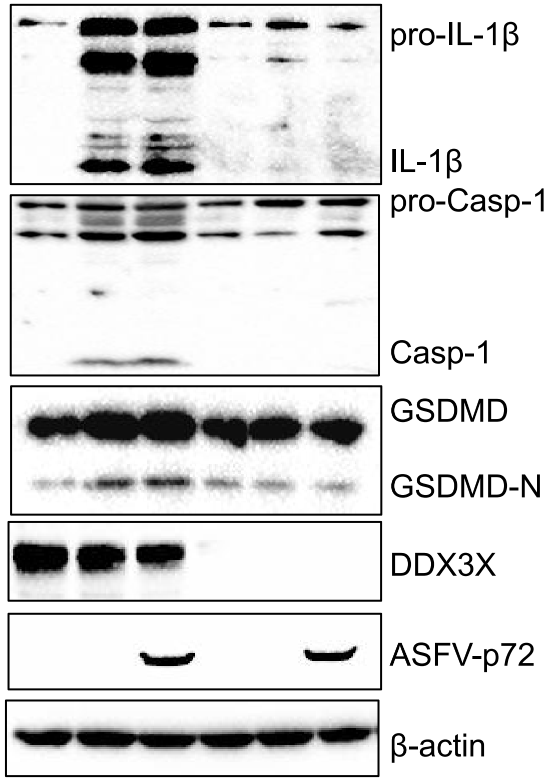

pro-IL-1β
IL-1β
pro-Casp-1
Casp-1
GSDMD
GSDMD-N
DDX3X
ASFV-p72
β-actin

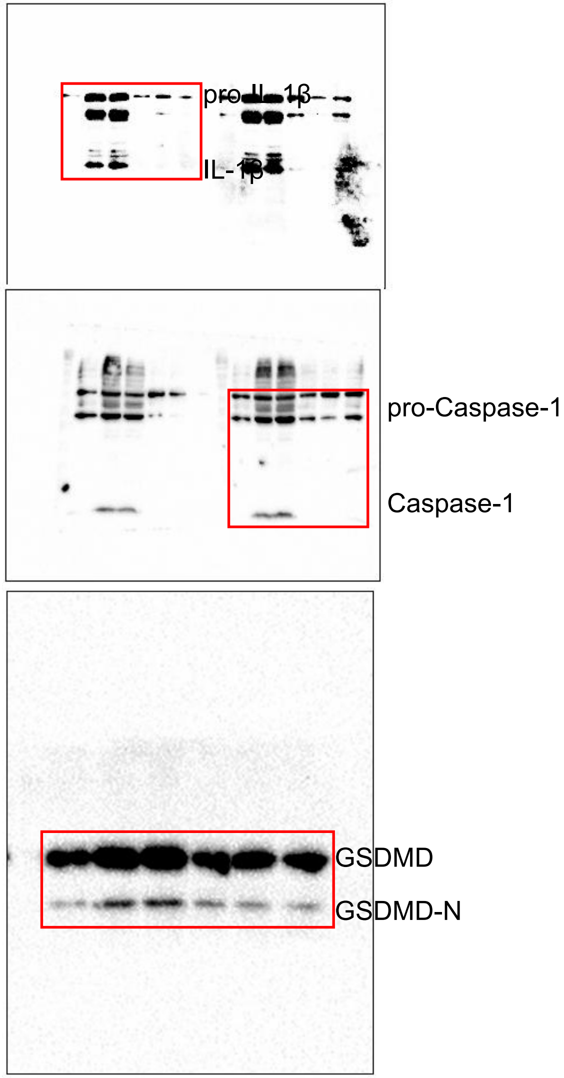

pro-IL-1β
IL-1β
pro-Caspase-1
Caspase-1
GSDMD
GSDMD-N

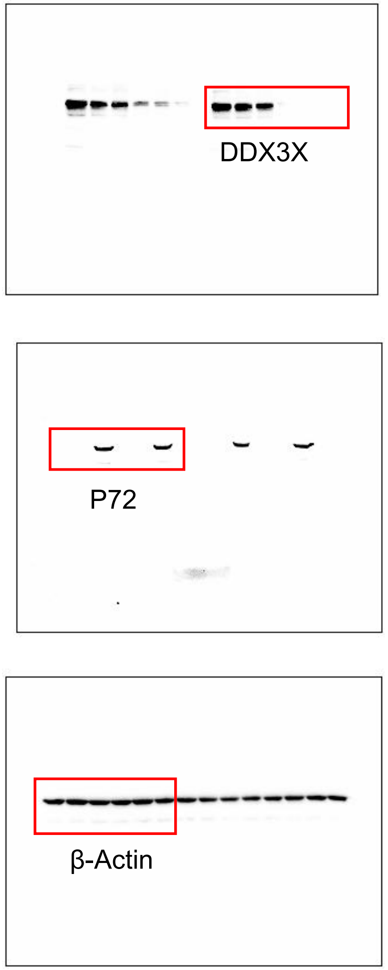

DDX3X
P72
β-Actin

Fig9F

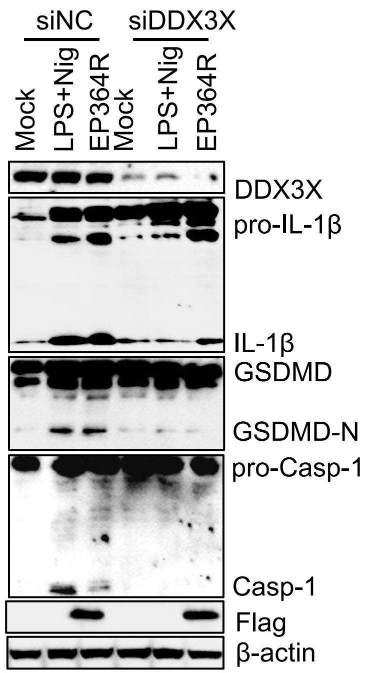
siNC
siDDX3X
Mock
LPS+Nig
EP364R
Mock
LPS+Nig
EP364R
DDX3X
pro-IL-1β
IL-1β
GSDMD
GSDMD-N
pro-Casp-1
Casp-1
Flag
β-actin

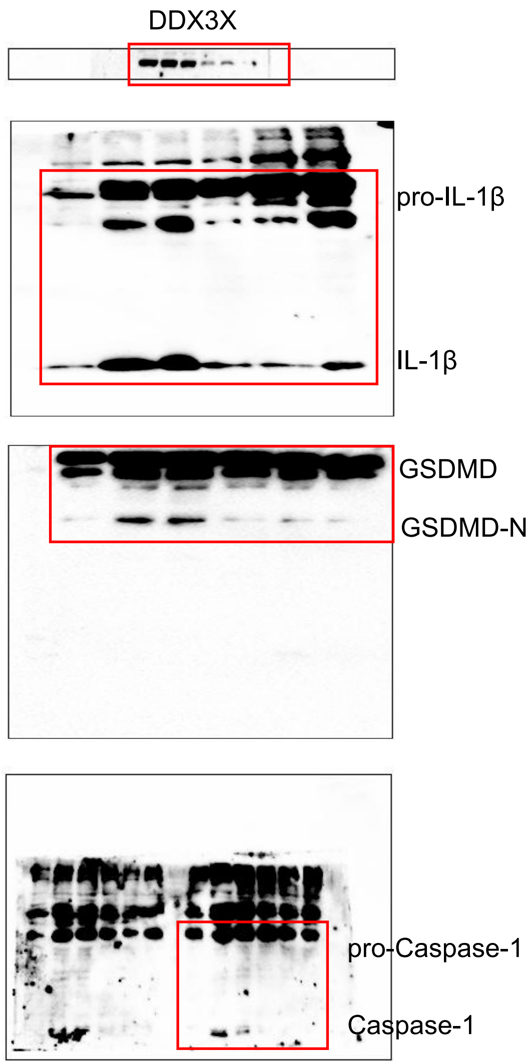
DDX3X
pro-IL-1β
IL-1β
GSDMD
GSDMD-N
pro-Caspase-1
Caspase-1

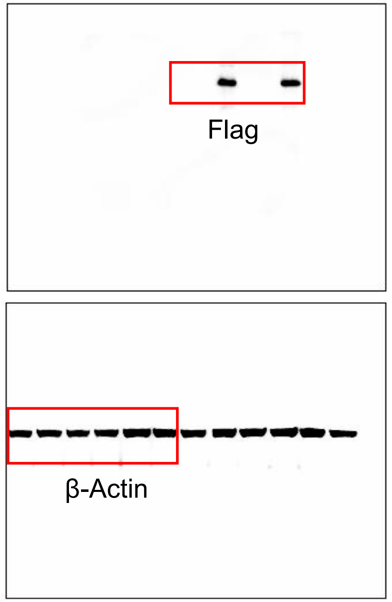
Flag
β-Actin

Fig9G

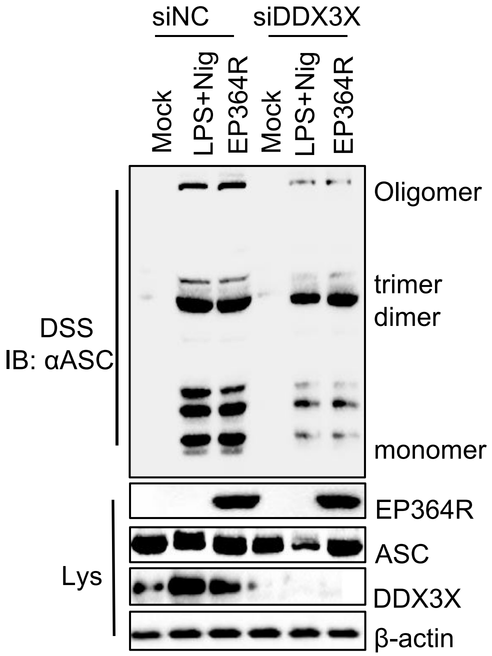
siNC
siDDX3X
Mock
LPS+Nig
EP364R
Mock
LPS+Nig
EP364R
Oligomer
trimer
dimer
monomer
DSS
IB: αASC
Lys
EP364R
ASC
DDX3X
β-actin

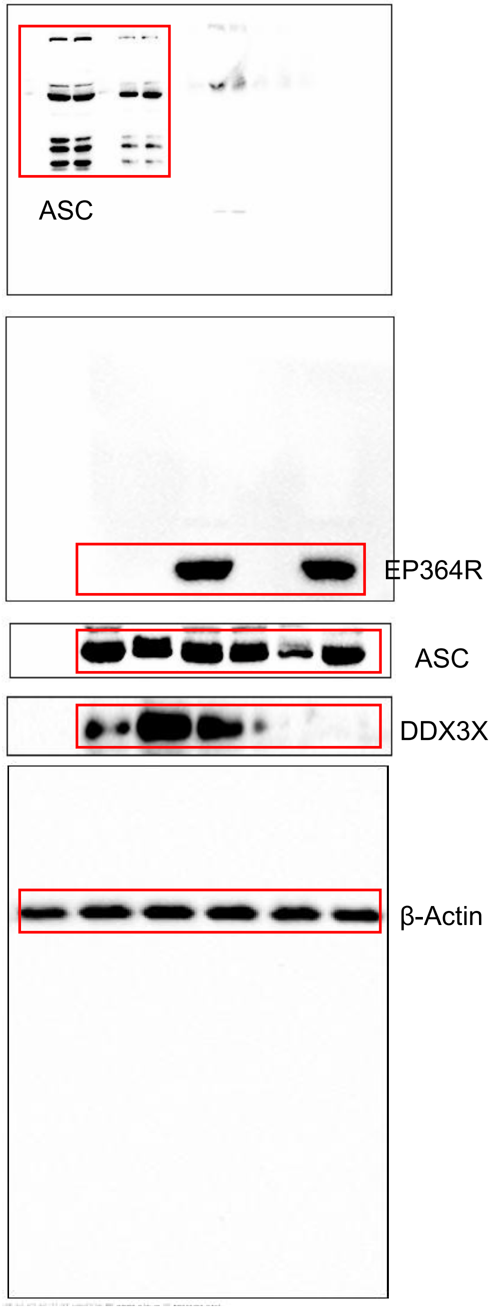
ASC
EP364R
ASC
DDX3X
β-Actin

Fig10A

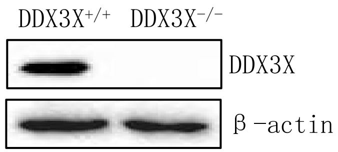

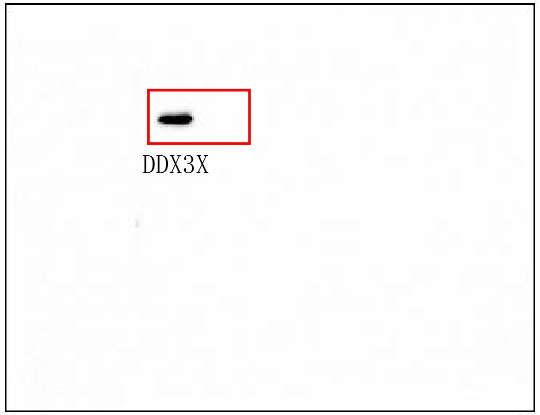

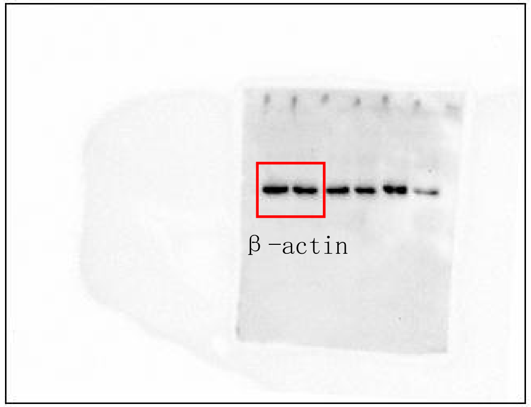

Fig10D

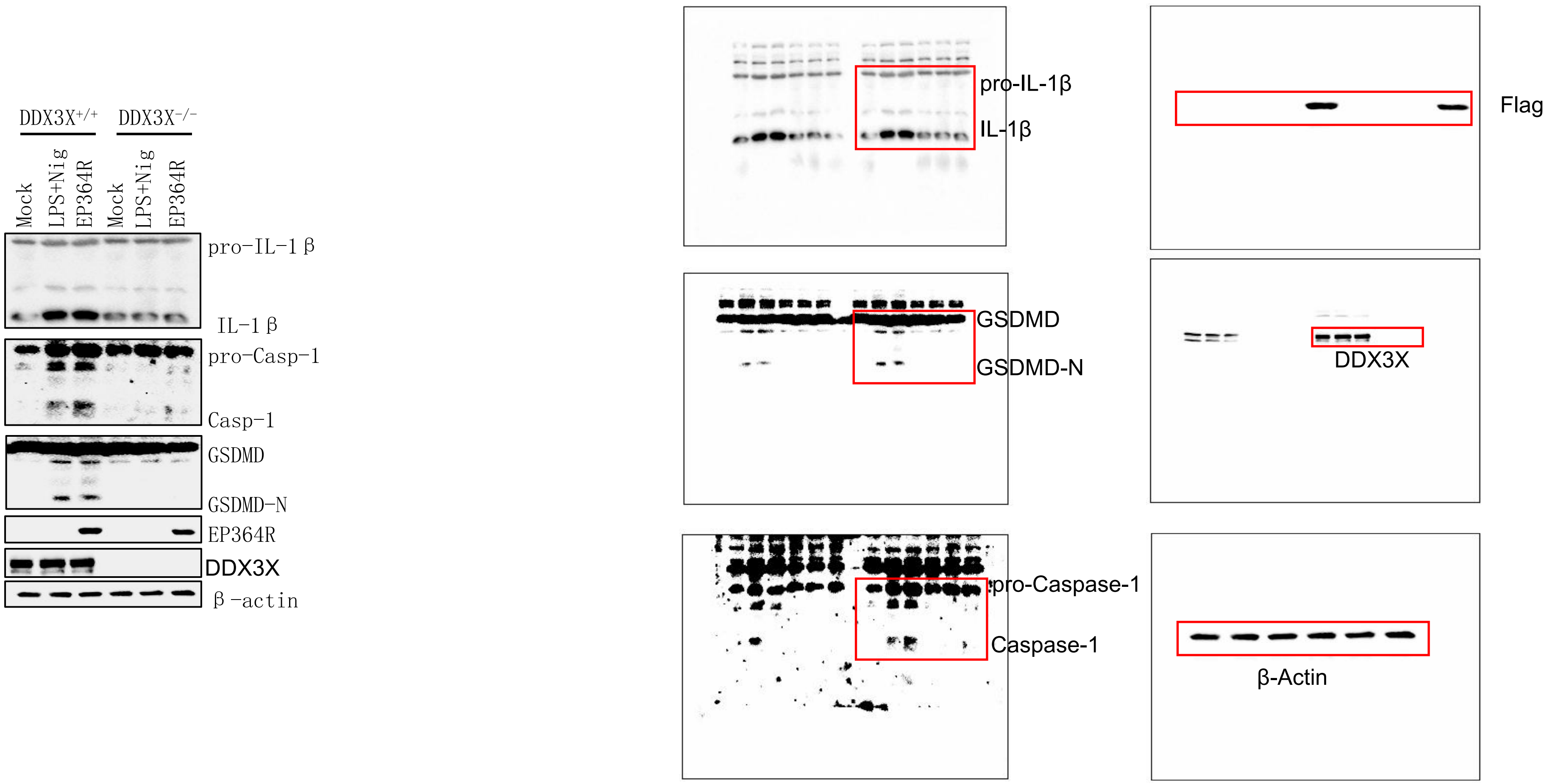
DDX3X+/+
DDX3X-/-
Mock
LPS+Nig
EP364R
Mock
LPS+Nig
EP364R
pro-IL-1β
IL-1β
pro-Casp-1
Casp-1
GSDMD
GSDMD-N
EP364R
DDX3X
β-actin
pro-IL-1β
IL-1β
Flag
GSDMD
GSDMD-N
DDX3X
pro-Caspase-1
Caspase-1
β-Actin

Fig10E

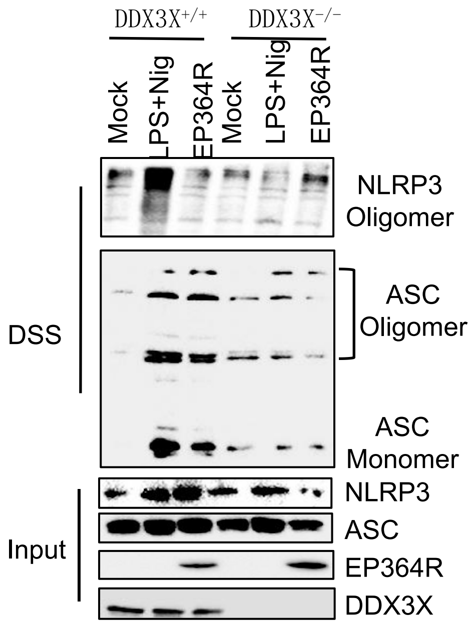

DDX3X+/+
DDX3X-/-
Mock
LPS+Nig
EP364R
Mock
LPS+Nig
EP364R
NLRP3 Oligomer
ASC Oligomer
ASC Monomer
DSS
NLRP3
ASC
EP364R
DDX3X
Input

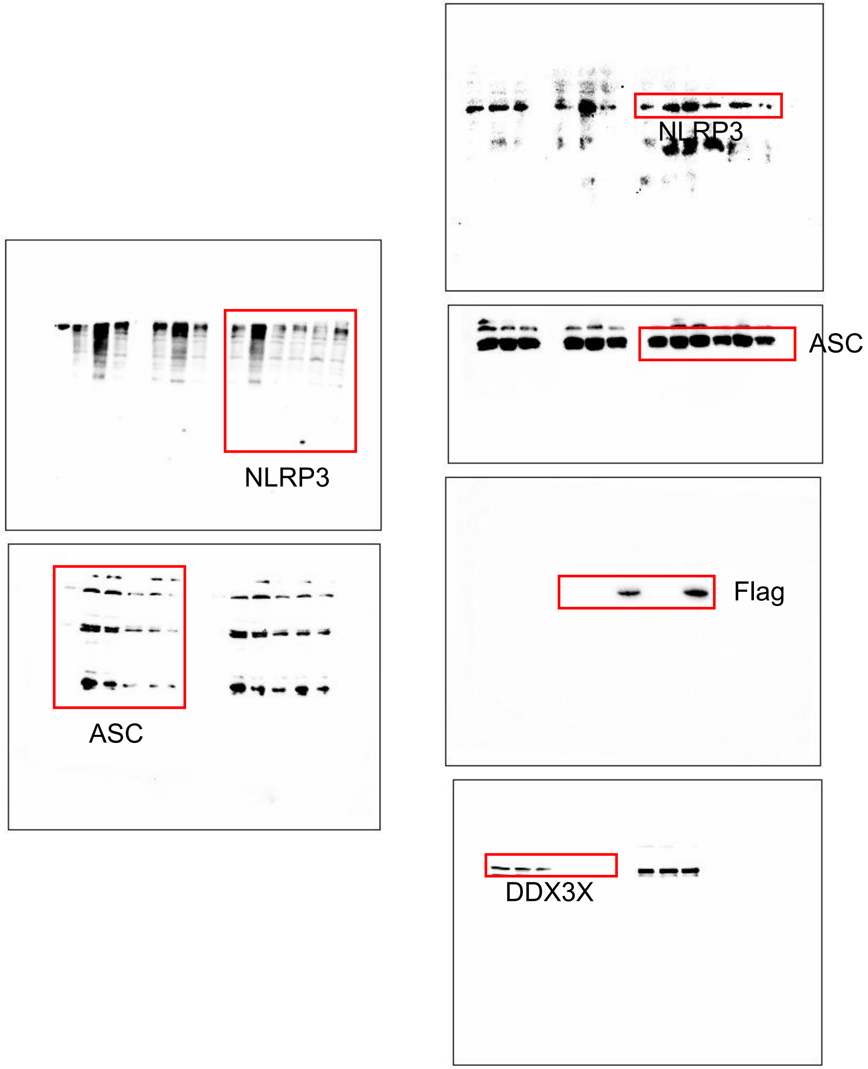

NLRP3
ASC
NLRP3
ASC
Flag
DDX3X

Fig10F

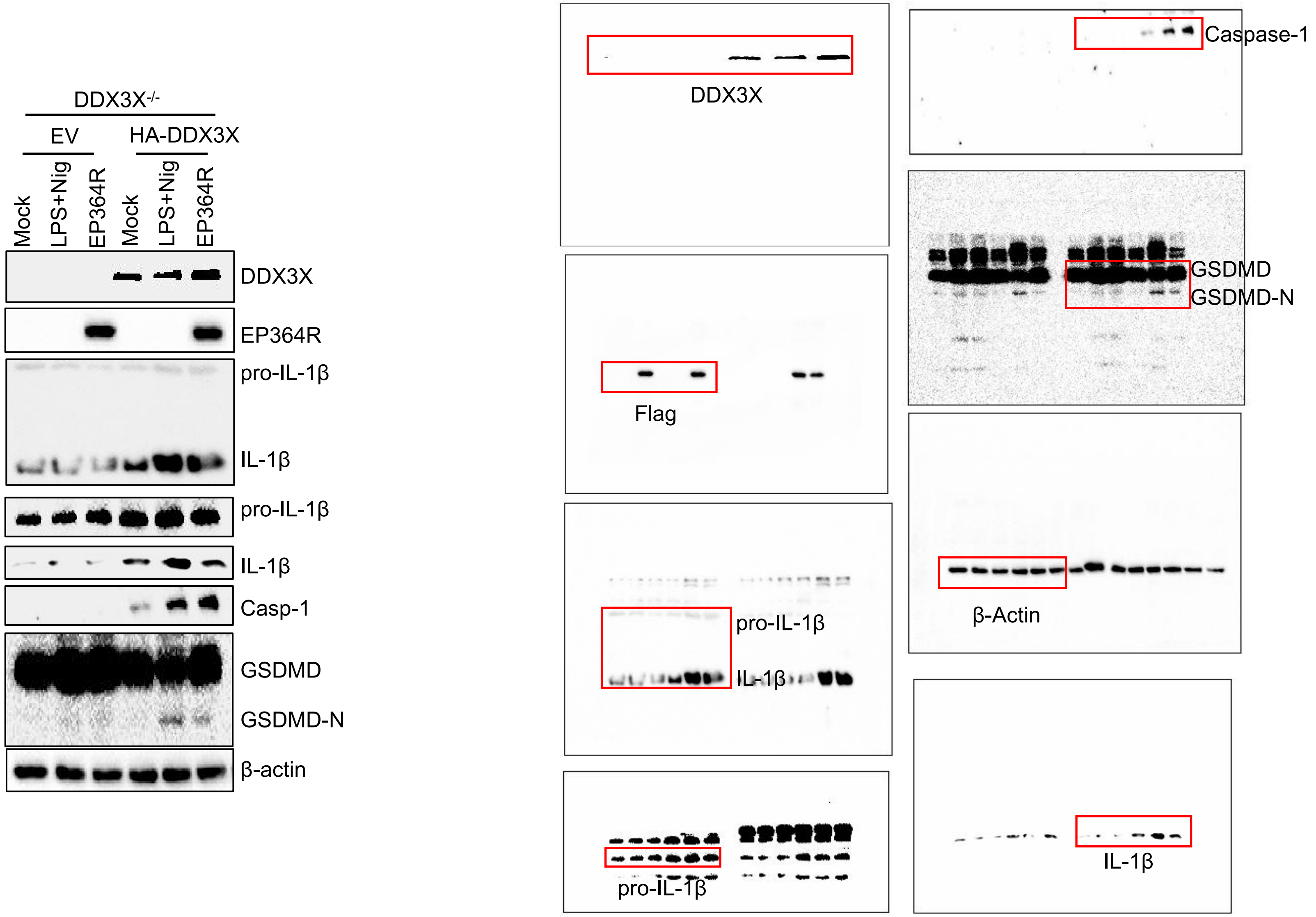
DDX3X-/-
EV
HA-DDX3X
Mock
LPS+Nig
EP364R
Mock
LPS+Nig
EP364R
DDX3X
EP364R
pro-IL-1β
IL-1β
pro-IL-1β
IL-1β
Casp-1
GSDMD
GSDMD-N
β-actin
DDX3X
Caspase-1
GSDMD
GSDMD-N
Flag
β-Actin
pro-IL-1β
IL-1β
pro-IL-1β
IL-1β

Fig10G

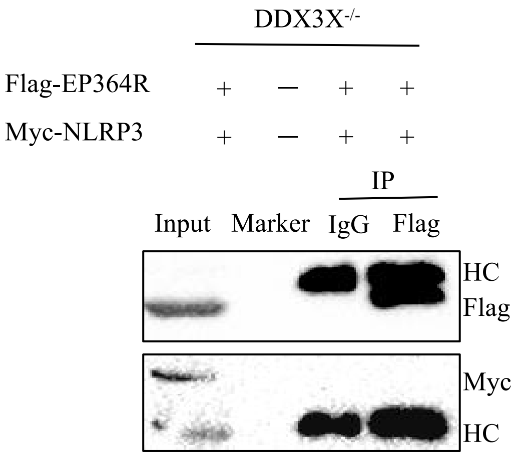
DDX3X-/-
Flag-EP364R + — + +
Myc-NLRP3 + — + +
IP
Input Marker IgG Flag
HC
Flag
Myc
HC

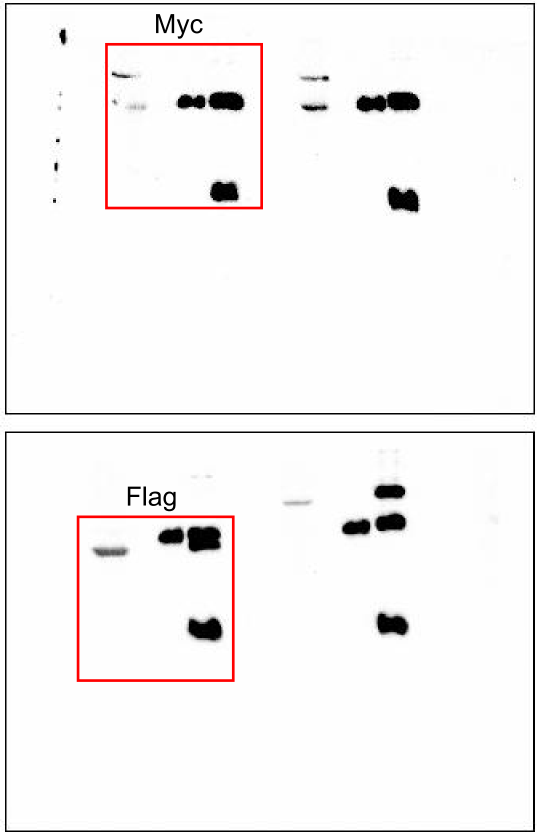
Myc
Flag

Fig11A

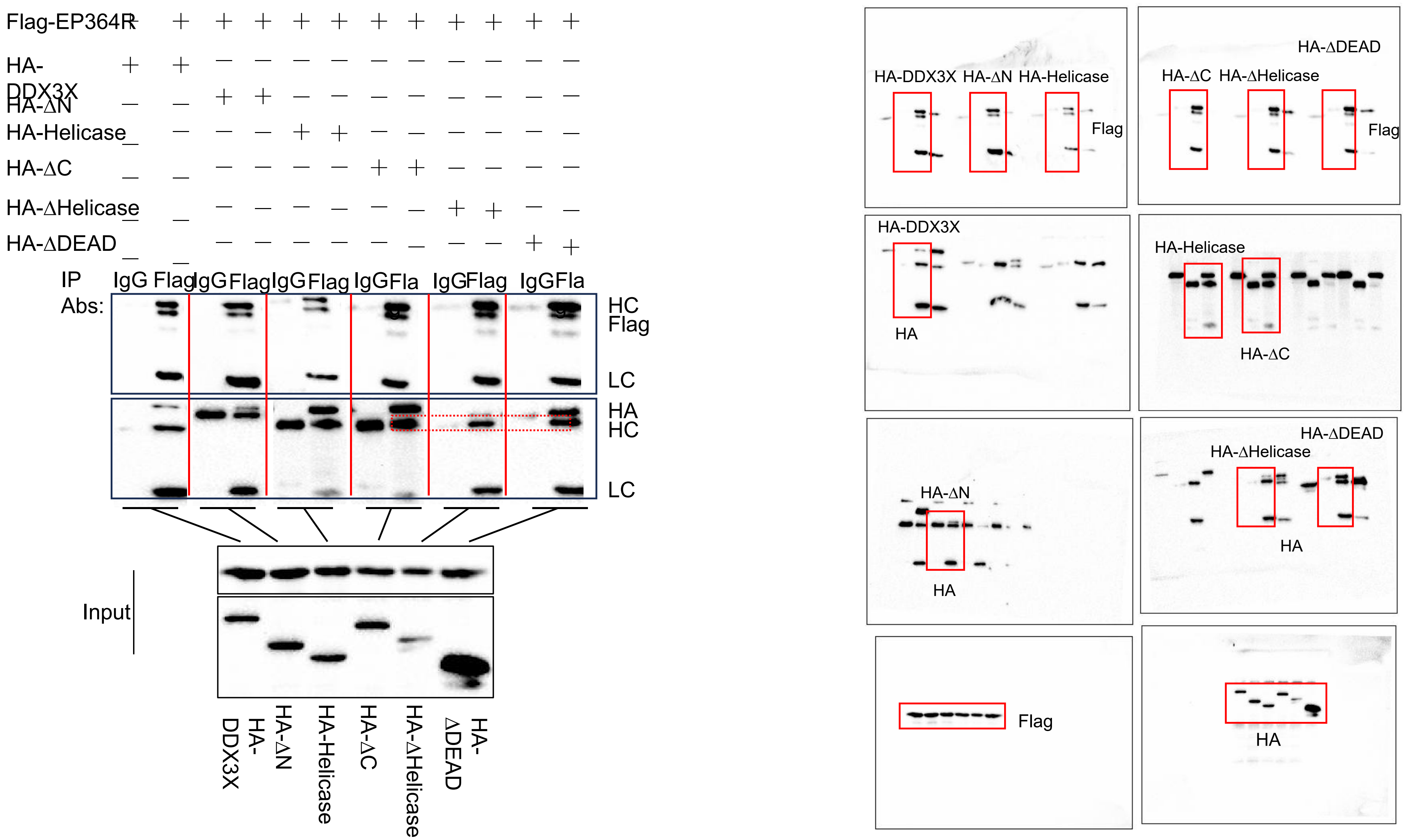
Flag-EP364R
HA-DDX3X
HA-ΔN
HA-Helicase
HA-ΔC
HA-ΔHelicase
HA-ΔDEAD
IP Abs:
IgG Flag IgG Flag IgG Flag IgG Fla IgG Flag IgG Fla
HC
Flag
LC
HA
HC
LC
Input
HA-DDX3X
HA-ΔN
HA-Helicase
HA-ΔC
HA-ΔHelicase
HA-ΔDEAD
HA-DDX3X HA-ΔN HA-Helicase
Flag
HA-ΔDEAD
HA-ΔC HA-ΔHelicase
Flag
HA-DDX3X
HA
HA-Helicase
HA-ΔC
HA-ΔN
HA
HA-ΔDEAD
HA-ΔHelicase
HA
Flag
HA

Fig11B

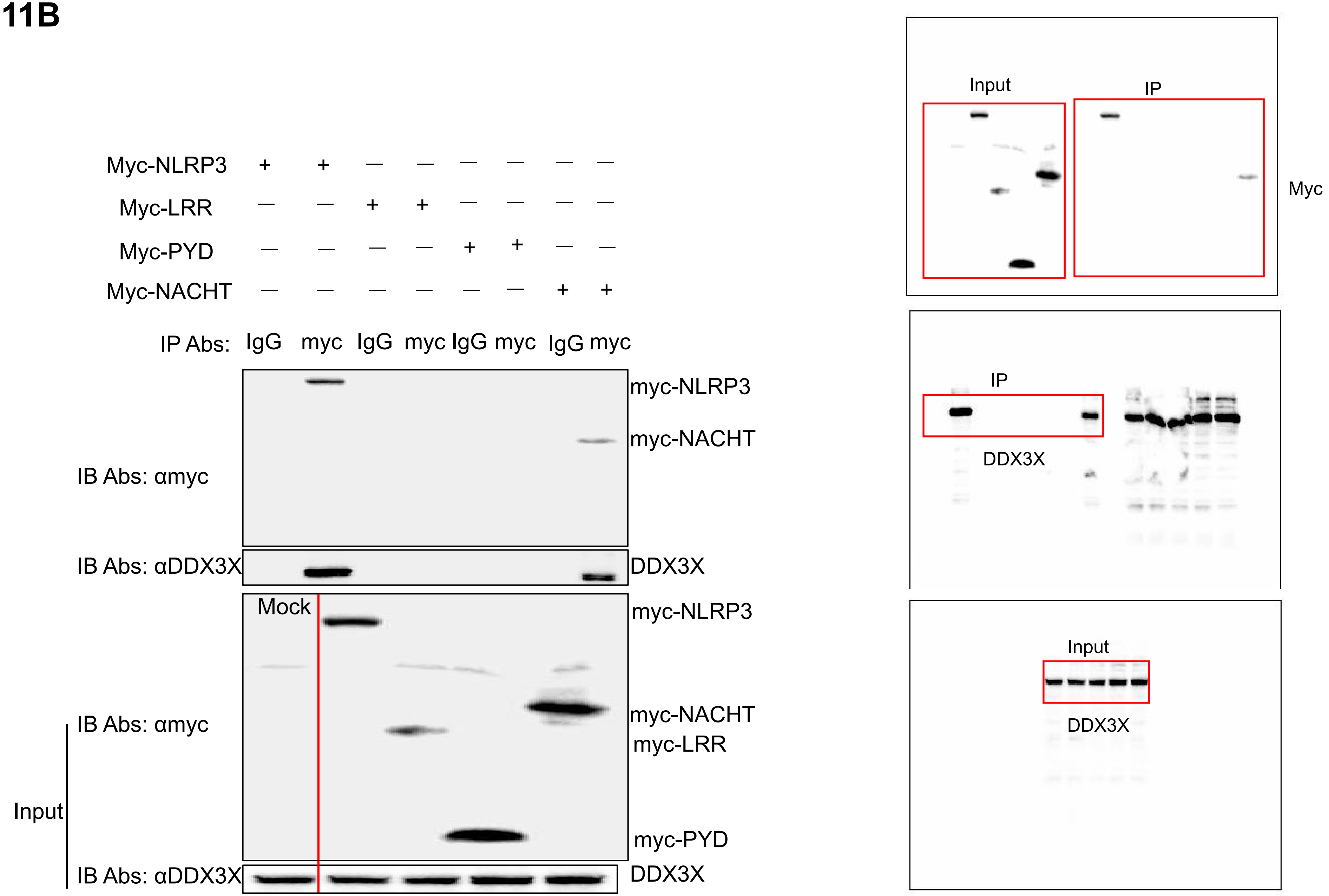

Myc-NLRP3 + + — — — — — —
Myc-LRR — — + + — — — —
Myc-PYD — — — — + + — —
Myc-NACHT — — — — — — + +
IP Abs: IgG myc IgG myc IgG myc IgG myc
myc-NLRP3
myc-NACHT
IB Abs: αmyc
IB Abs: αDDX3X
DDX3X
Mock
myc-NLRP3
IB Abs: αmyc
myc-NACHT
myc-LRR
Input
myc-PYD
IB Abs: αDDX3X
DDX3X
Input
IP
Myc
IP
DDX3X
Input
DDX3X

Fig11C

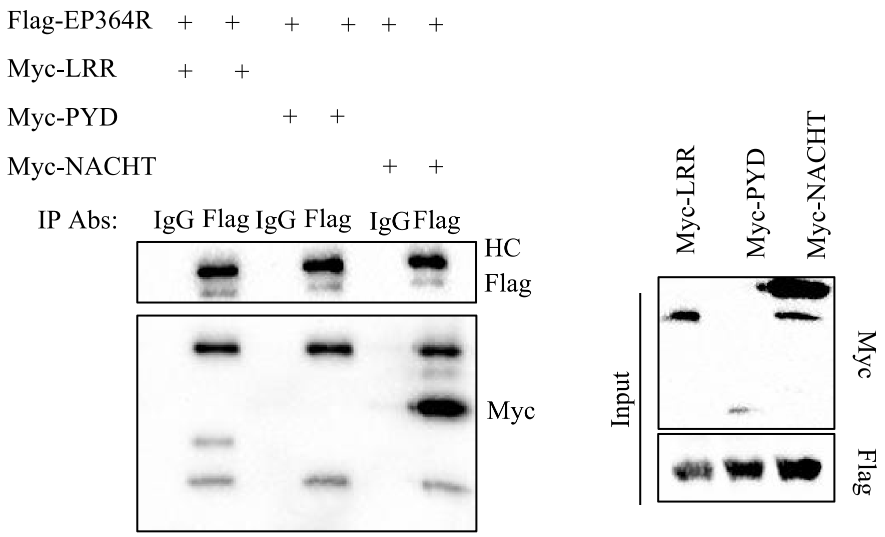

Flag-EP364R + + + + + +
Myc-LRR + +
Myc-PYD + +
Myc-NACHT + +
IP Abs: IgG Flag IgG Flag IgG Flag
HC
Flag
Myc
Myc-LRR
Myc-PYD
Myc-NACHT
Input
Myc
Flag

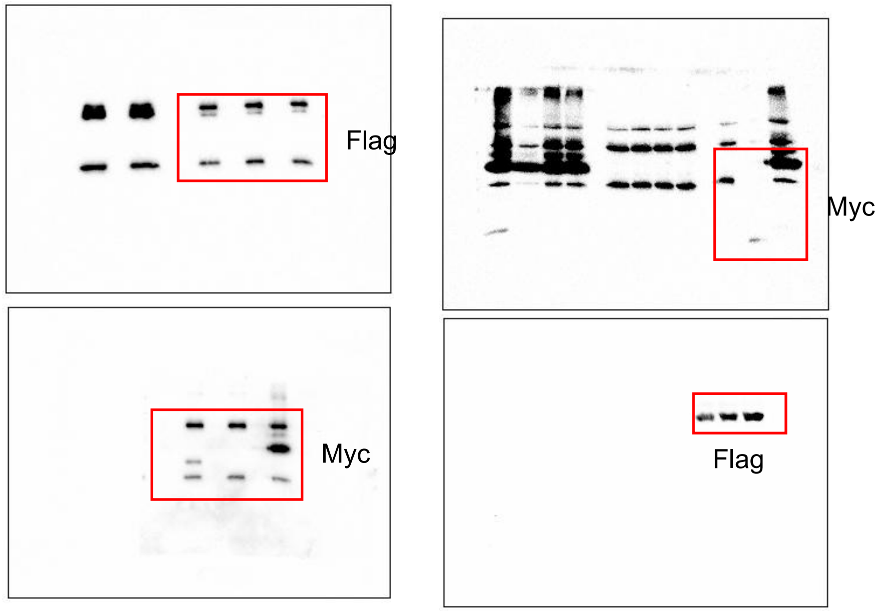

Flag
Myc
Myc
Flag

FigS1A

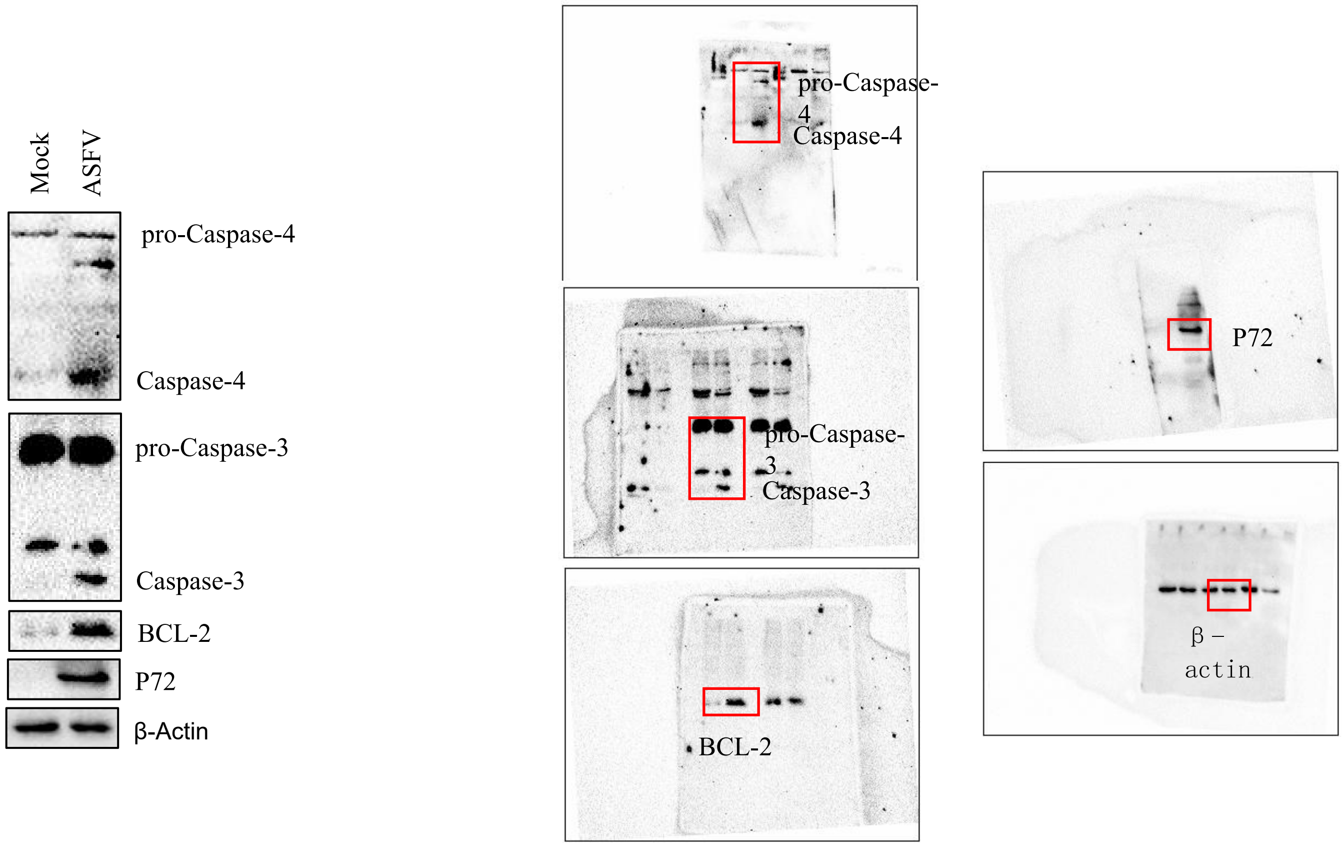
Mock
ASFV
pro-Caspase-4
Caspase-4
pro-Caspase-3
Caspase-3
BCL-2
P72
β-Actin
pro-Caspase-4
Caspase-4
pro-Caspase-3
Caspase-3
BCL-2
P72
β－actin

FigS1C

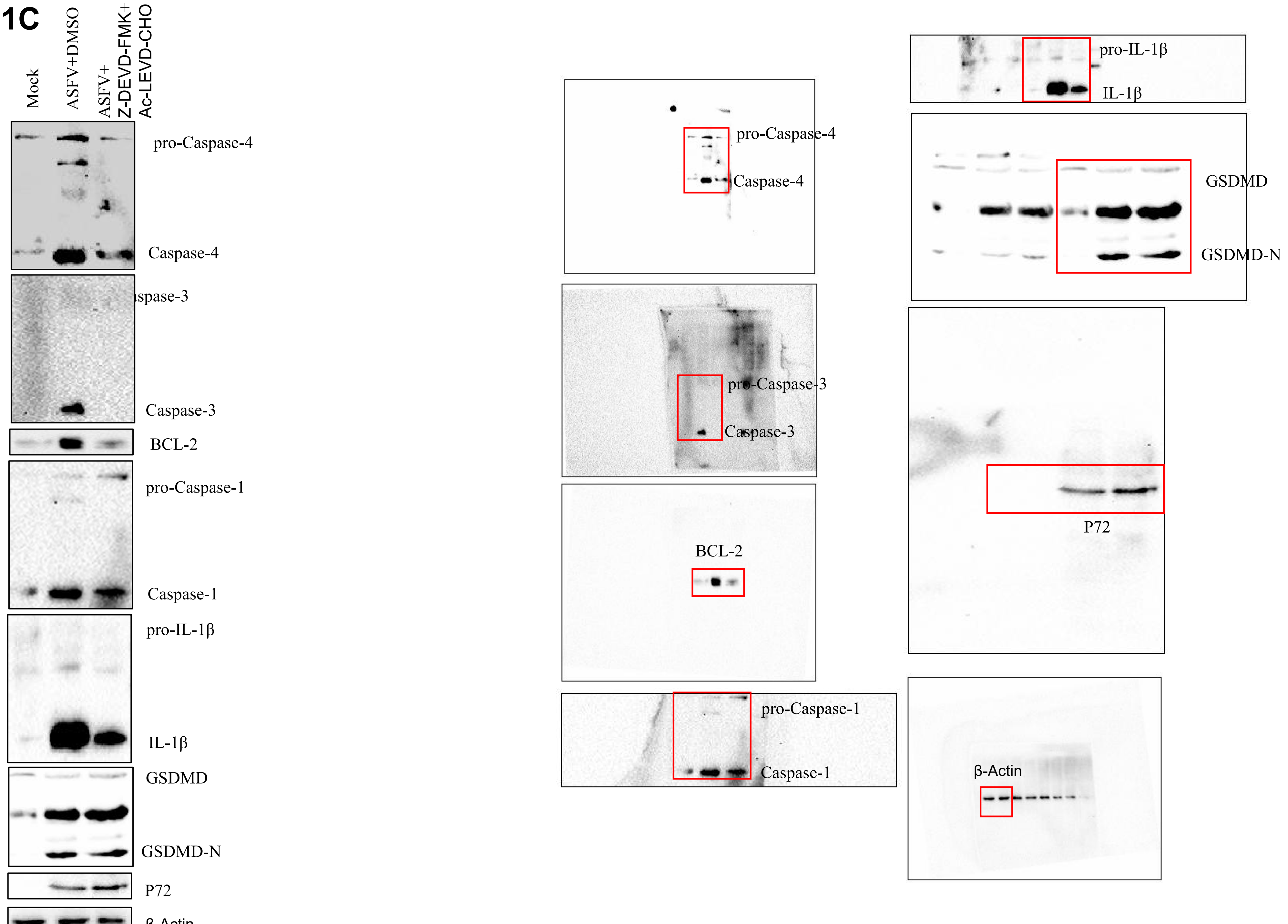
Mock
ASFV+DMSO
ASFV+
Z-DEVD-FMK+
Ac-LEVD-CHO
pro-Caspase-4
Caspase-4
Caspase-3
BCL-2
pro-Caspase-1
Caspase-1
pro-IL-1β
IL-1β
GSDMD
GSDMD-N
P72
pro-Caspase-4
Caspase-4
pro-Caspase-3
Caspase-3
BCL-2
pro-Caspase-1
Caspase-1
pro-IL-1β
IL-1β
GSDMD
GSDMD-N
P72
β-Actin

FigS2A

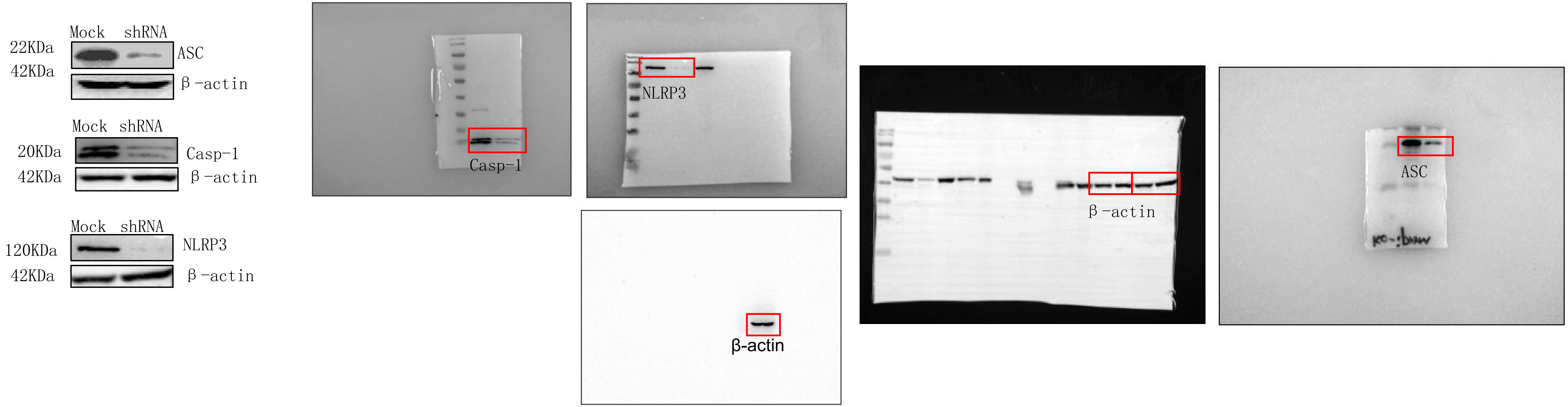
Mock
shRNA
22KDa
ASC
42KDa
β -actin
Mock
shRNA
20KDa
Casp-1
42KDa
β -actin
Mock
shRNA
120KDa
NLRP3
42KDa
β -actin
Casp-1
NLRP3
β-actin
β -actin
ASC

FigS3D

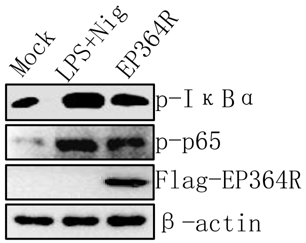
Mock
LPS+Nig
EP364R
p-IκBα
p-p65
Flag-EP364R
β-actin

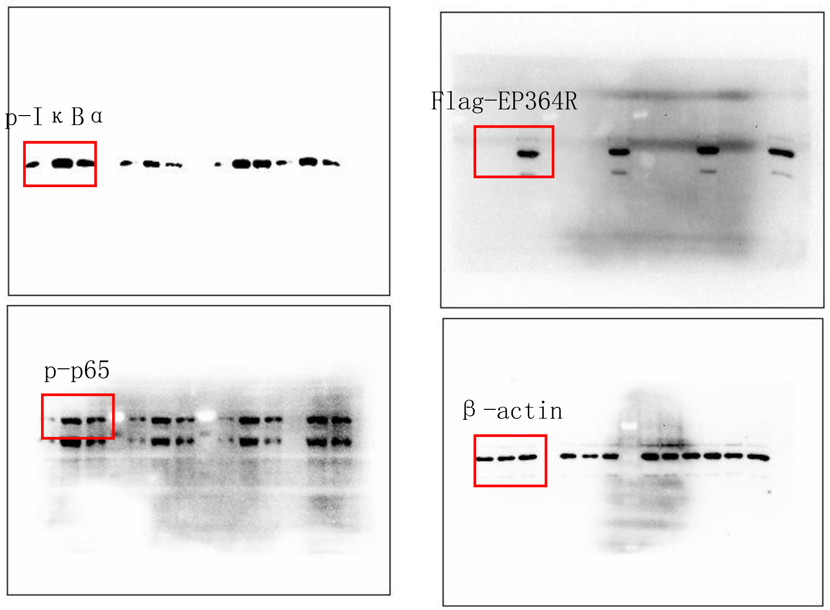
p-IκBα
Flag-EP364R
p-p65
β-actin

FigS5A

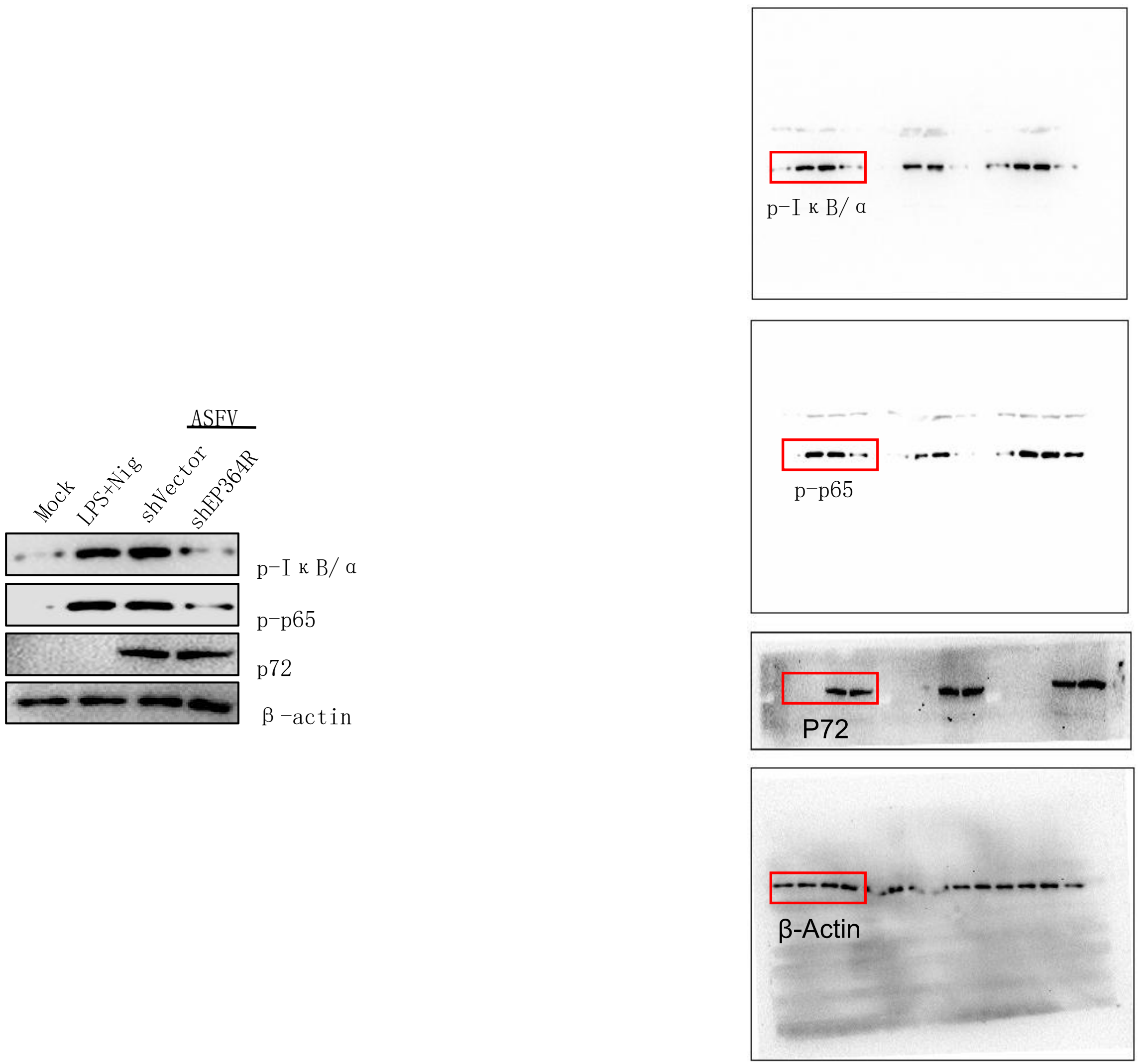
ASFV
Mock
LPS+Nig
shVector
shEP364R
p-IκB/α
p-p65
p72
β-actin
p-IκB/α
p-p65
P72
β-Actin

FigS7A

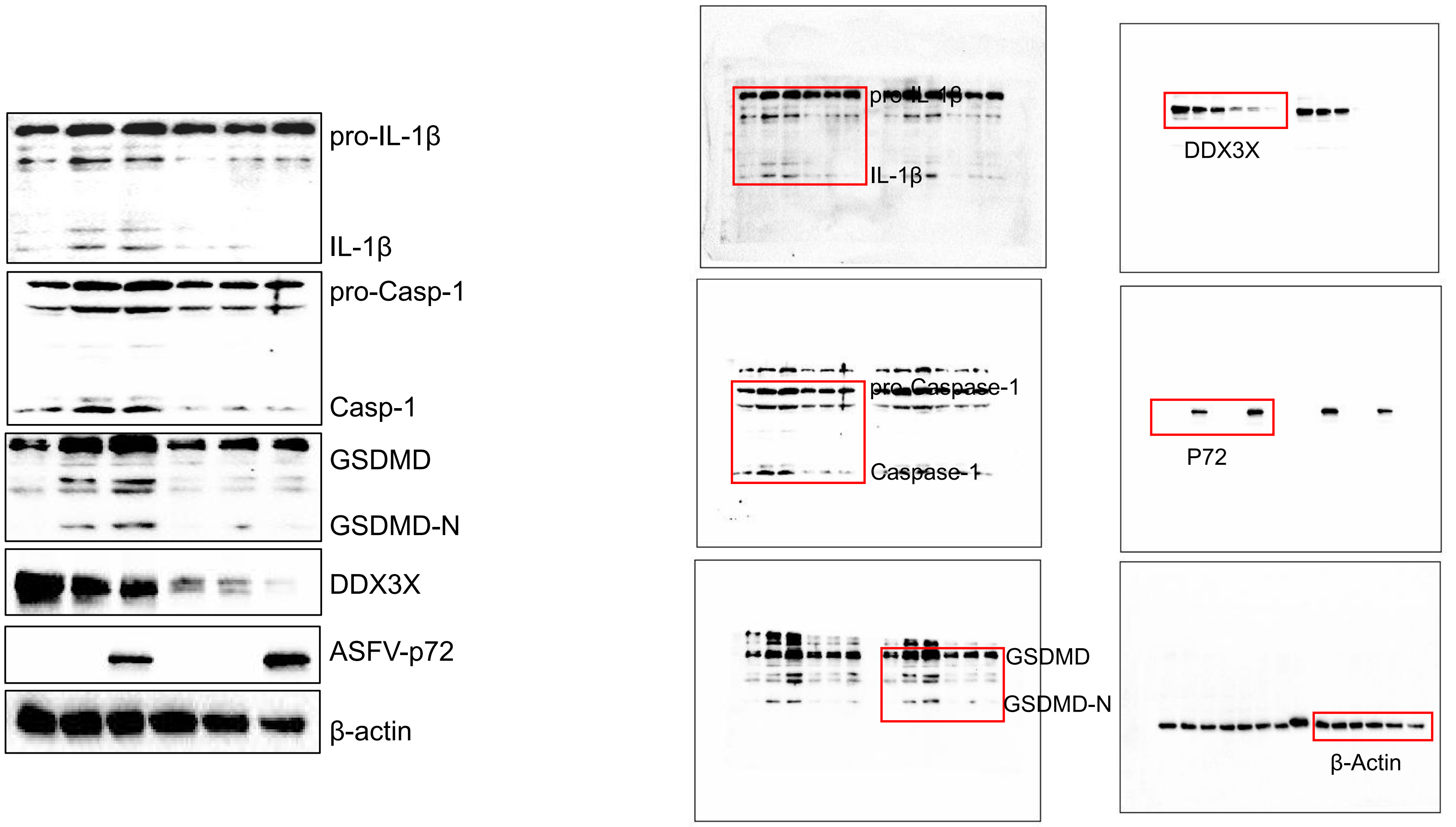
pro-IL-1β
IL-1β
pro-Casp-1
Casp-1
GSDMD
GSDMD-N
DDX3X
ASFV-p72
β-actin
pro-IL-1β
IL-1β
pro-Caspase-1
Caspase-1
GSDMD
GSDMD-N
DDX3X
P72
β-Actin

FigS8C

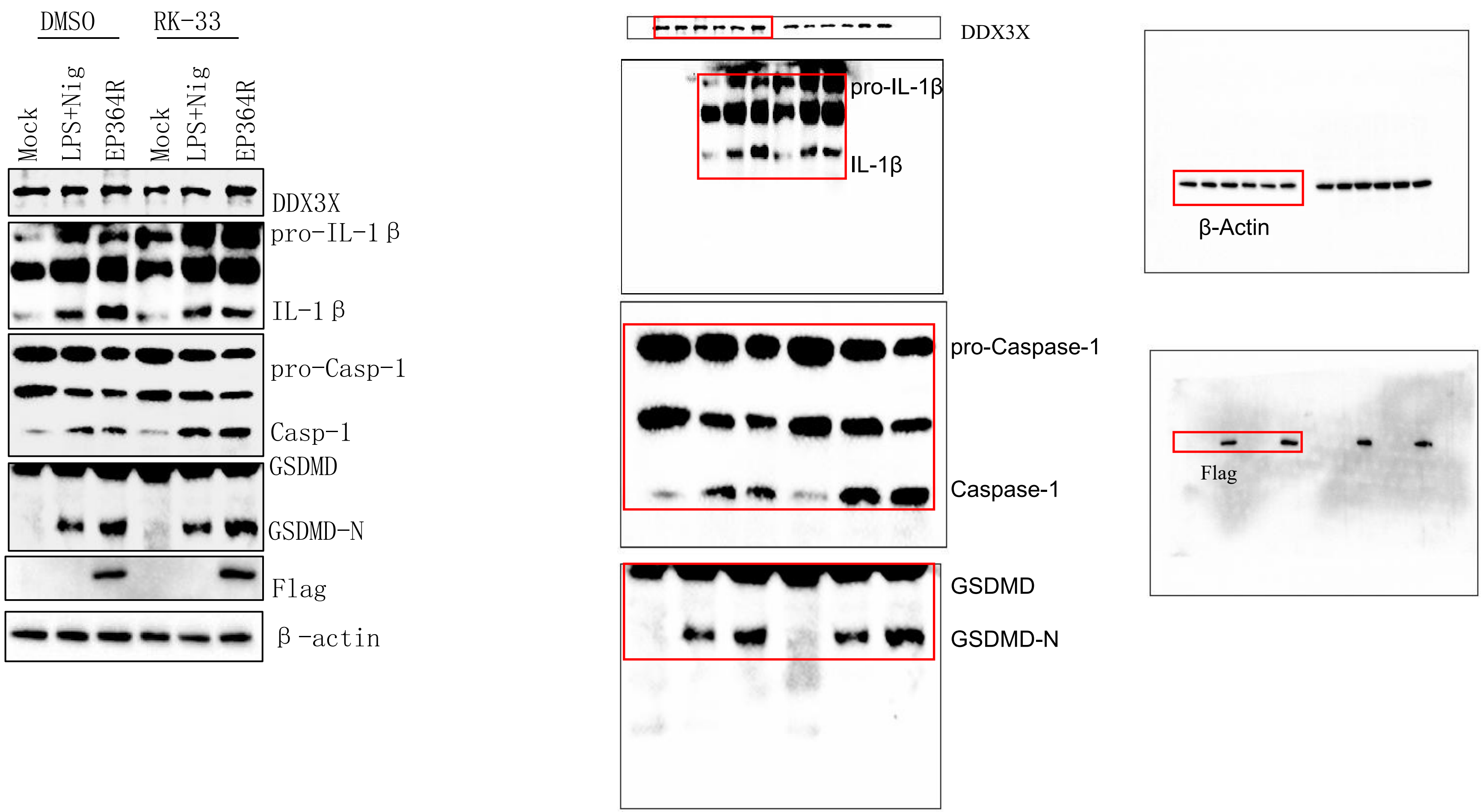
DMSO
RK-33
Mock
LPS+Nig
EP364R
Mock
LPS+Nig
EP364R
DDX3X
pro-IL-1β
IL-1β
pro-Casp-1
Casp-1
GSDMD
GSDMD-N
Flag
β-actin
DDX3X
pro-IL-1β
IL-1β
pro-Caspase-1
Caspase-1
GSDMD
GSDMD-N
β-Actin
Flag

FigS8D

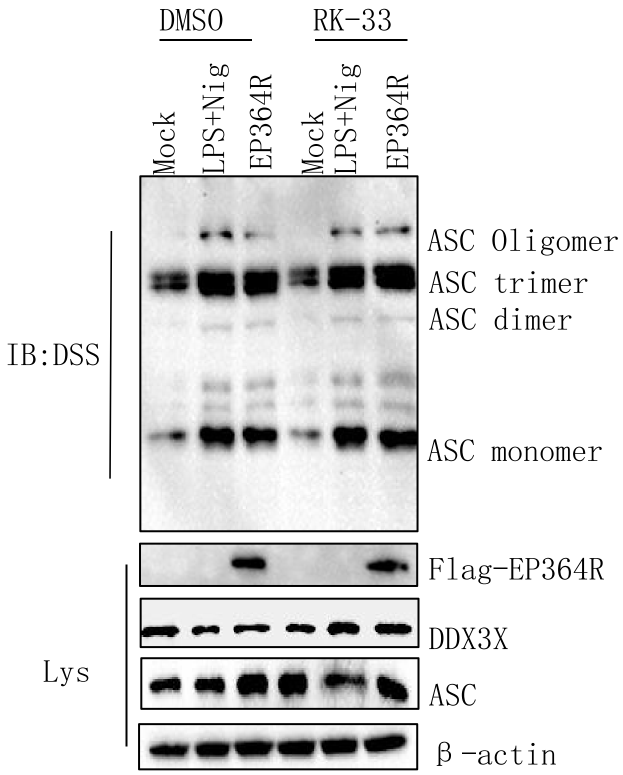
DMSO
RK-33
Mock
LPS+Nig
EP364R
Mock
LPS+Nig
EP364R
IB:DSS
ASC Oligomer
ASC trimer
ASC dimer
ASC monomer
Lys
Flag-EP364R
DDX3X
ASC
β-actin

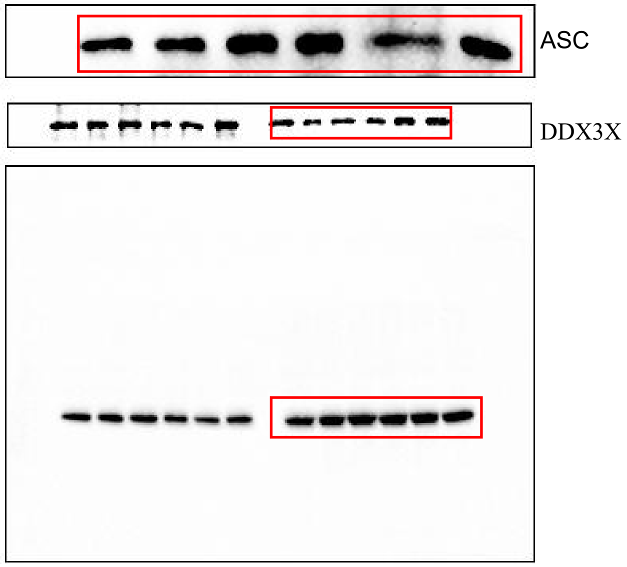
ASC
DDX3X

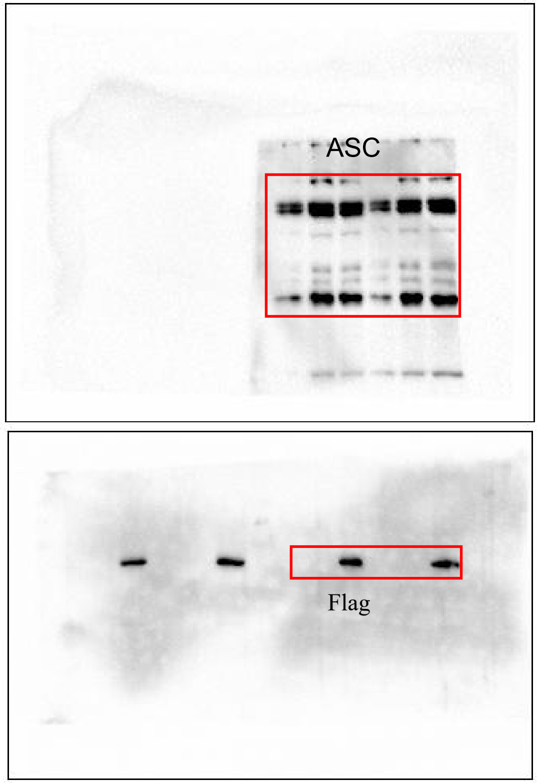
ASC
Flag

FigS10H,J

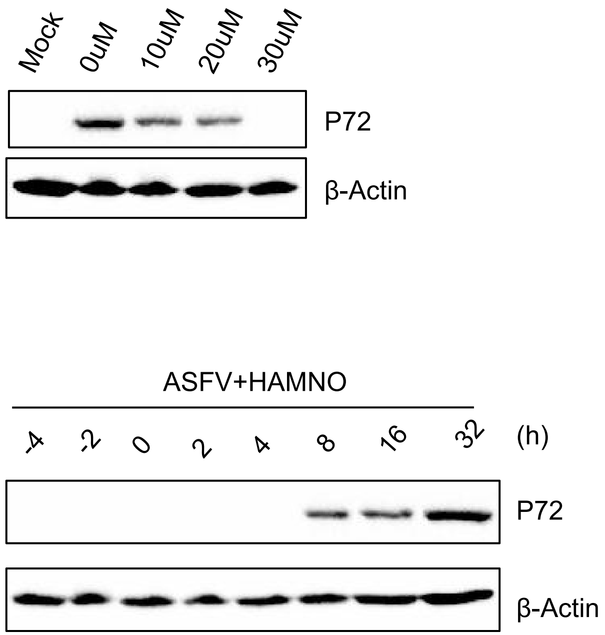

Mock
0uM
10uM
20uM
30uM
P72
β-Actin
ASFV+HAMNO
-4
-2
0
2
4
8
16
32
(h)
P72
β-Actin

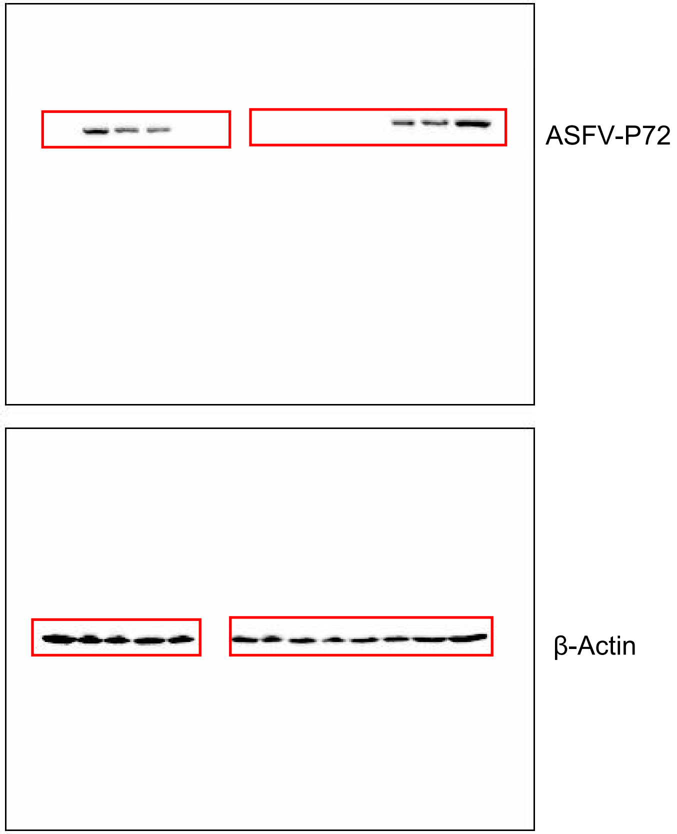

ASFV-P72
β-Actin

FigS10L

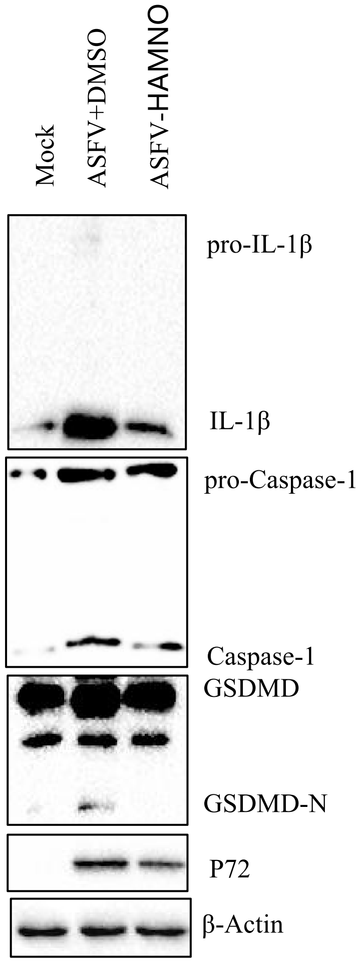

Mock
ASFV+DMSO
ASFV-HAMNO
pro-IL-1β
IL-1β
pro-Caspase-1
Caspase-1
GSDMD
GSDMD-N
P72
β-Actin

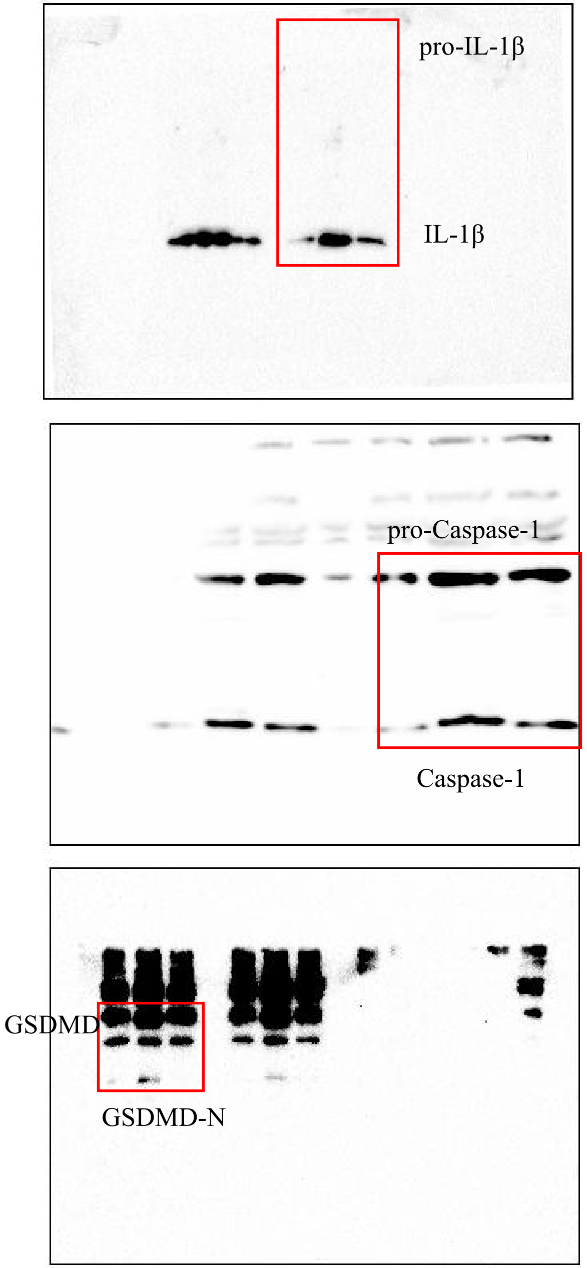

pro-IL-1β
IL-1β
pro-Caspase-1
Caspase-1
GSDMD
GSDMD-N

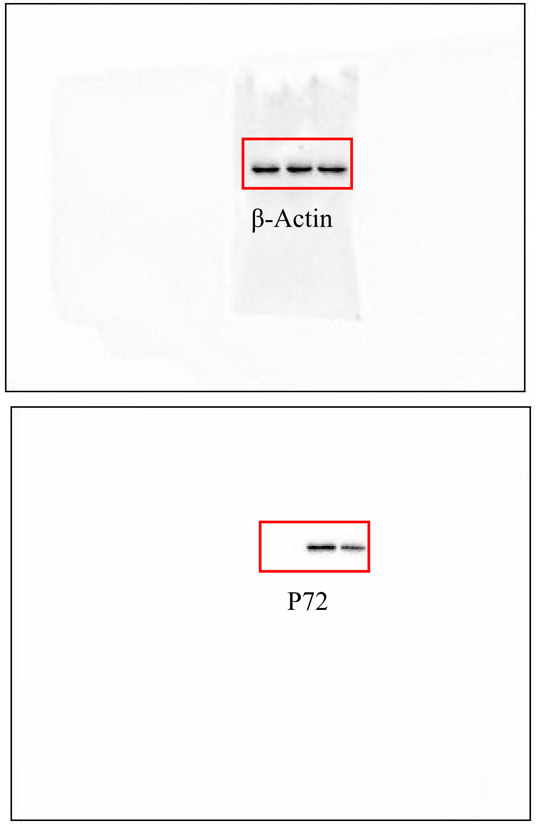

β-Actin
P72

FigS10M

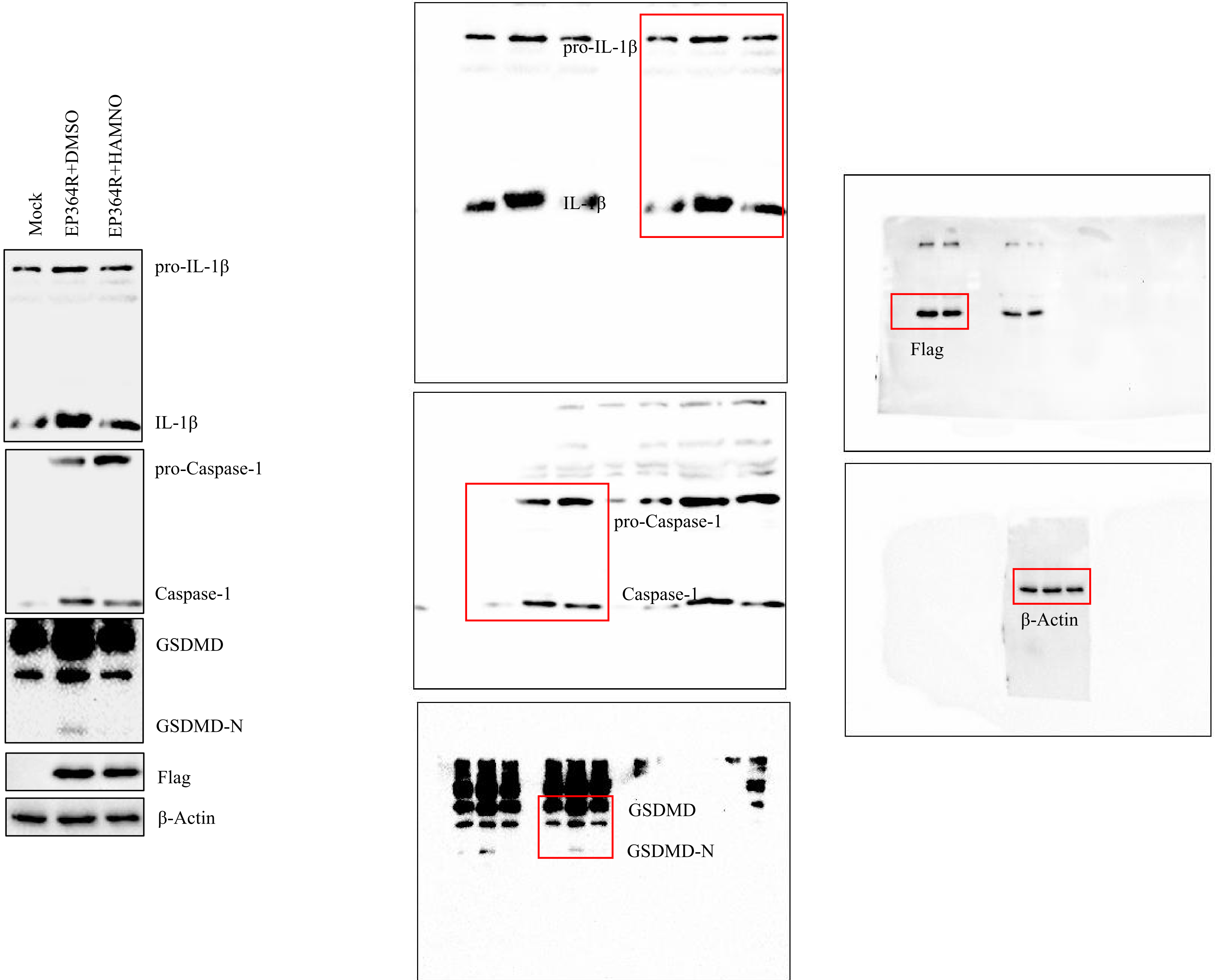
Mock
EP364R+DMSO
EP364R+HAMNO
pro-IL-1β
IL-1β
pro-Caspase-1
Caspase-1
GSDMD
GSDMD-N
Flag
β-Actin
pro-IL-1β
IL-1β
pro-Caspase-1
Caspase-1
GSDMD
GSDMD-N
Flag
β-Actin

FigS10O

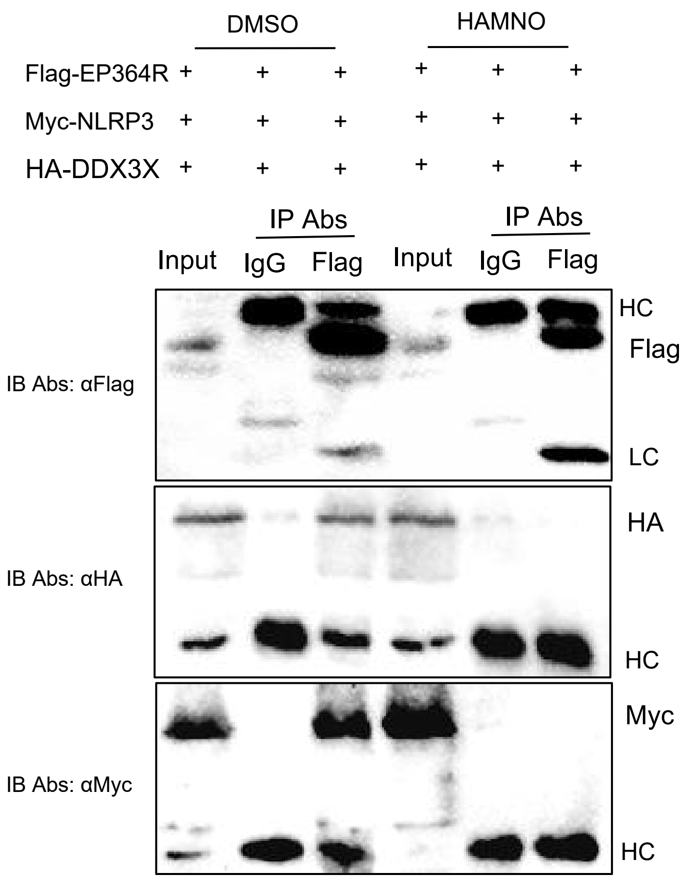
DMSO
HAMNO
Flag-EP364R + + + + + +
Myc-NLRP3 + + + + + +
HA-DDX3X + + + + + +
IP Abs
IP Abs
Input
IgG
Flag
Input
IgG
Flag
IB Abs: αFlag
IB Abs: αHA
IB Abs: αMyc
HC
Flag
LC
HA
HC
Myc
HC

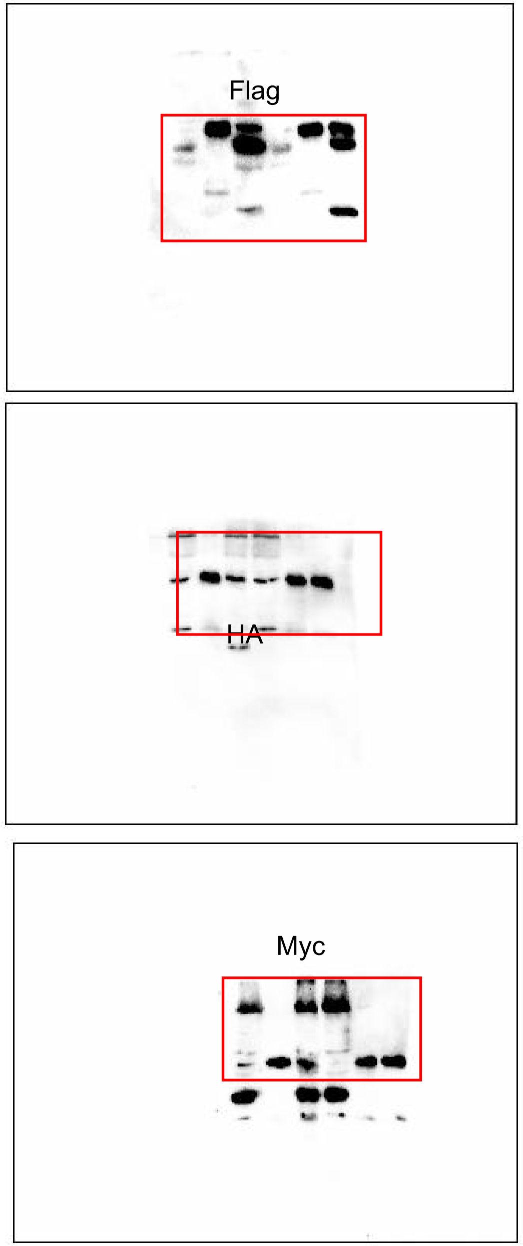
Flag
HA
Myc
