# Supplementary material for: African swine fever virus pEP364R acts as an important inflammatory-inducing factor to activate NLRP3 inflammasome-mediated pyroptosis by regulating DDX3X
Source: PLoS Pathog. 2026 Feb 25;22(2):e1013874. doi: 10.1371/journal.ppat.1013874 (PMC12952717; doi:10.1371/journal.ppat.1013874)
Supplement: S13 Data — (ZIP) [file ppat.1013874.s024.zip › S13_Data/Fig11/D/DDX3X.pdf]

Predictor of Natural Disordered Regions

PONDR® tutorial

New Prediction | DEPP prediction

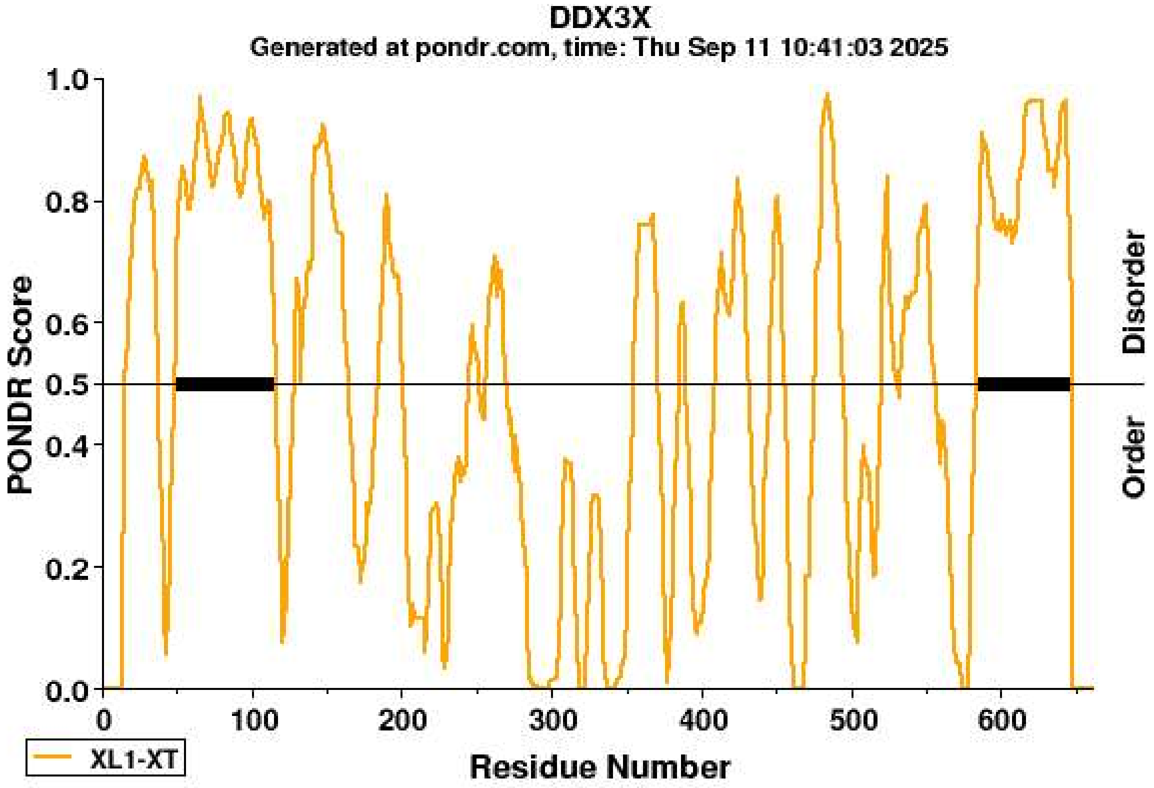

Note: Click on the picture to zoom in.

PONDR Protein Disorder Predictor
Developed by P. Romero, X. Li, A.K. Dunker, Z. Obradovic, E. Garner.

VL3 Predictor
Developed by P. Radivojac and A.K. Dunker

```
                DEPP Predictor
Developed by P. Radivojac

                VSL2 Predictor
Developped by K. Peng and Z. Obradovic

================================XL1_XT NNP STATISTICS================================
Predicted residues: 661                 Number Disordered Regions: 14
Number residues disordered: 337         Longest Disordered Region: 68
Overall percent disordered: 50.98       Average Prediction Score: 0.4834
Predicted disorder segment [15]-[37]    Average Strength= 0.7433
Predicted disorder segment [49]-[116]   Average Strength= 0.8421
Predicted disorder segment [128]-[164]  Average Strength= 0.7339
Predicted disorder segment [184]-[200]  Average Strength= 0.6714
Predicted disorder segment [245]-[252]  Average Strength= 0.5450
Predicted disorder segment [257]-[269]  Average Strength= 0.6353
Predicted disorder segment [355]-[371]  Average Strength= 0.7057
Predicted disorder segment [386]-[389]  Average Strength= 0.5943
Predicted disorder segment [409]-[431]  Average Strength= 0.6762
Predicted disorder segment [446]-[455]  Average Strength= 0.6902
Predicted disorder segment [476]-[494]  Average Strength= 0.8001
Predicted disorder segment [520]-[530]  Average Strength= 0.6419
Predicted disorder segment [534]-[555]  Average Strength= 0.6624
Predicted disorder segment [583]-[647]  Average Strength= 0.8413

================================PREDICTOR OUTPUT================================
           "D" = Disordered                        " " = Ordered
================================================================================

1        MSHVAVENAL GLDQQFAGLD LNSSDNQSGG STASKGRYIP PHLRNREATK
XL1_XT                  DDDDDD DDDDDDDDDD DDDDDDD            DD


51       GFYDKDSSRW SSNKDKDAYS SFGSRNDSRG KSSFFSDRGS GSRGRFDDRG
XL1_XT   DDDDDDDDDD DDDDDDDDDD DDDDDDDDDD DDDDDDDDDD DDDDDDDDDD


101      RSDYDGIGSR GDRGGFGKYE RGGHSRWCDK SDEDDWSKPL PPSERLEQEL
XL1_XT   DDDDDDDDDD DDDDDD            DDD DDDDDDDDDD DDDDDDDDDD


151      FSGGNTGINF EKYDDIPVEA TGNNCPPHIE SFSDVEMGEI IMGNIELTRY
XL1_XT   DDDDDDDDDD DDDD                     DDDDDDD DDDDDDDDDD


201      TRPTPVQKHA IPIIKEKRDL MACAQTGSGK TAAFLLPILS QIYSDGPGEA
XL1_XT                                                   DDDDDD


251      LRAMKENGRY GRRKQYPISL VLAPTRELAV QIYEEARKFS YRSRVRPCVV
XL1_XT   DD    DDDD DDDDDDDDD


301      YGGADIGQQI RDLERGCHLL VATPGRLVDM MERGKIGLDF CKYLVLDEAD
XL1_XT


351      RMLDMGFEPQ IRRIVEQDTM PPKGVRHTMM FSATFPKEIQ MLARDFLDEY
XL1_XT       DDDDDD DDDDDDDDDD D               DDDD


401      IFLAVGRVGS TSENITQKVV WVEESDKRSF LLDLLNATGK DSLTLVFVET
XL1_XT           DD DDDDDDDDDD DDDDDDDDDD D               DDDDD


451      KKGADSLEDF LYHEGYACTS IHGDRSQRDR EEALHQFRSG KSPILVATAV
XL1_XT   DDDDD                      DDDDD DDDDDDDDDD DDDD


501      AARGLDISNV KHVINFDLPS DIEEYVHRIG RTGRVGNLGL ATSFFNERNA
```

```
XL1_XT                       D DDDDDDDDDD    DDDDDDD DDDDDDDDDD

551      NITKDLLDLL VEAKQEVPSW LENMAYEHHY KGSSRGRSKS RFSGGFGARD
XL1_XT   DDDDD                           DDDDDDDD DDDDDDDDDD

601      YRQSSGASSS SFSSSRASSS RSGGGGHGSS RGFGGGGYGG FYNSDGYGGN
XL1_XT   DDDDDDDDDD DDDDDDDDDD DDDDDDDDDD DDDDDDDDDD DDDDDDD

651      YNSQGVDWWG N
XL1_XT
```
