# Supplementary material for: African swine fever virus pEP364R acts as an important inflammatory-inducing factor to activate NLRP3 inflammasome-mediated pyroptosis by regulating DDX3X
Source: PLoS Pathog. 2026 Feb 25;22(2):e1013874. doi: 10.1371/journal.ppat.1013874 (PMC12952717; doi:10.1371/journal.ppat.1013874)
Supplement: S13 Data — (ZIP) [file ppat.1013874.s024.zip › S13_Data/Fig11/D/EP364R.pdf]

Predictor of Natural Disordered Regions

PONDR® tutorial

New Prediction | DEPP prediction

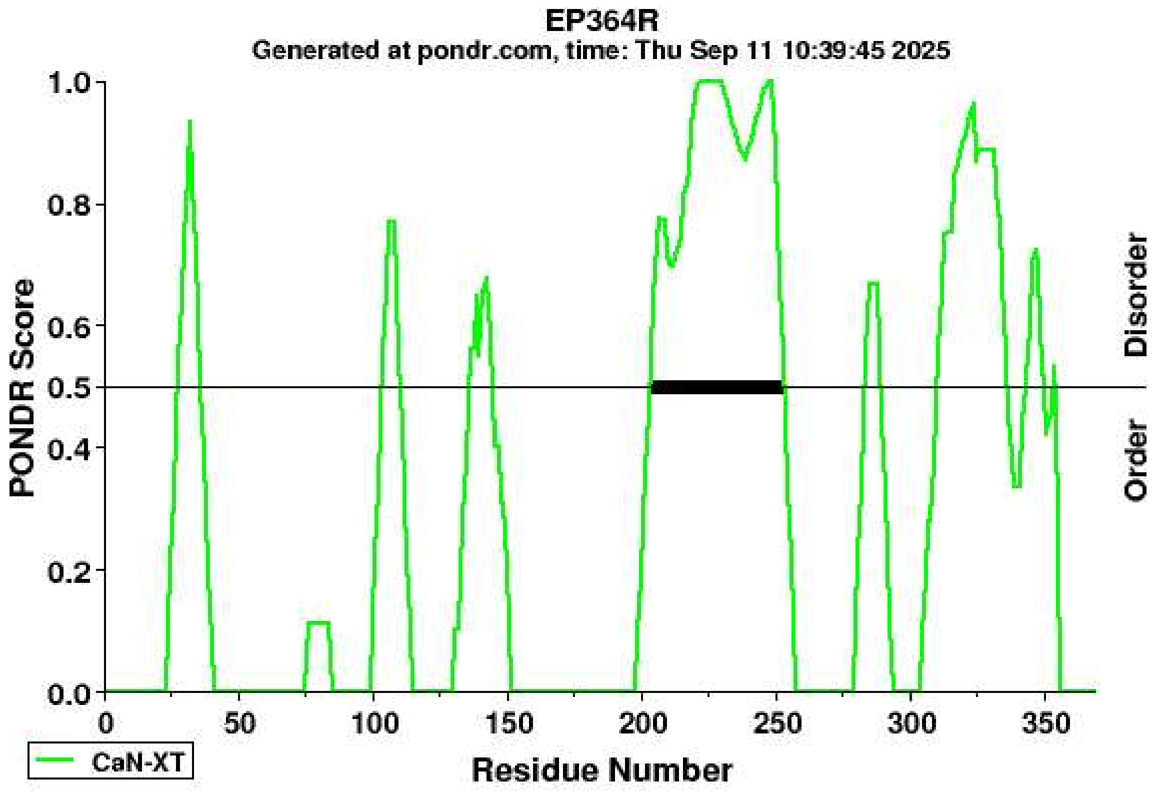

Note: Click on the picture to zoom in.

PONDR Protein Disorder Predictor
Developed by P. Romero, X. Li, A.K. Dunker, Z. Obradovic, E. Garner.

VL3 Predictor
Developed by P. Radivojac and A.K. Dunker

```
                DEPP Predictor
Developed by P. Radivojac

                VSL2 Predictor
Developped by K. Peng and Z. Obradovic

================================CAN_XT NNP STATISTICS================================
Predicted residues: 369                 Number Disordered Regions: 8
Number residues disordered: 113         Longest Disordered Region: 50
Overall percent disordered: 30.62       Average Prediction Score: 0.2968
Predicted disorder segment [28]-[36]    Average Strength= 0.7074
Predicted disorder segment [104]-[110]  Average Strength= 0.6745
Predicted disorder segment [137]-[144]  Average Strength= 0.6121
Predicted disorder segment [204]-[253]  Average Strength= 0.8726
Predicted disorder segment [284]-[289]  Average Strength= 0.6296
Predicted disorder segment [311]-[336]  Average Strength= 0.8200
Predicted disorder segment [344]-[349]  Average Strength= 0.6446
Predicted disorder segment [354]-[354]  Average Strength= 0.5332

================================PREDICTOR OUTPUT================================
           "D" = Disordered                         " " = Ordered
================================================================================

1        MYFLVADHRE HHVIPFLKTD FHHMHQNPIQ KNQALLEIKQ LFTGDYLICK
CAN_XT                                DDD DDDDDD


51       SPSTILACIE RKTYKDFAAS LKDGRYKNRQ KMLSLREQTN CQLYFFVEGP
CAN_XT


101      AFPNPQKKIN HVAYASIITA MTHLMVRDHI FVIQTKNEAH SSQKLVQLFY
CAN_XT      DDDDDDD                             DDDD DDDD


151      AFSKEMVCVV PTSLTPTDEE LCIKLWSSLS GISGVIGKIL ANTCSVAHLV
CAN_XT


201      HGKLSSQNID QLKTPSNRPF PKKVKRMLIS ISKGNKELEI KLLSGVPNIG
CAN_XT      DDDDDDD DDDDDDDDDD DDDDDDDDDD DDDDDDDDDD DDDDDDDDDD


251      KKLAAEILKD HALLFFLNQP VECLANIQIV QKTRTIKLGM KRAEAIHYFL
CAN_XT   DDD                                 DDDDDD


301      NWCGSAHVTD DSQNITEASR STMQVATQSA AIQPAATQPL HEVSDDASSD
CAN_XT              DDDDDDDDDD DDDDDDDDDD DDDDDD        DDDDDD


351      ASSPVGYQTL SKEMLLNTA
CAN_XT      D
```
