# Supplementary material for: African swine fever virus pEP364R acts as an important inflammatory-inducing factor to activate NLRP3 inflammasome-mediated pyroptosis by regulating DDX3X
Source: PLoS Pathog. 2026 Feb 25;22(2):e1013874. doi: 10.1371/journal.ppat.1013874 (PMC12952717; doi:10.1371/journal.ppat.1013874)
Supplement: S1 Text — (DOCX) [file ppat.1013874.s033.docx]

In ASFV-infected porcine tissues such as the lung, the viral protein EP364R activates the NLRP3 inflammasome via the host factor DDX3X, triggering pyroptosis and inflammatory responses. Cytokines and damage-associated molecular patterns (DAMPs) released during cell death induce a cytokine storm, ultimately leading to systemic physiological dysfunction. The natural small-molecule compound HAMNO, identified through screening, specifically targets EP364R and disrupts its interaction with DDX3X. This suppresses both EP364R- and ASFV-induced pyroptosis and inflammatory activation (thereby locking the inflammatory dragon), while also inhibiting ASFV replication. As a result, physiological homeostasis is restored.
